# Understanding medication safety involving patient transfer from intensive care to hospital ward: a qualitative sociotechnical factor study

**DOI:** 10.1136/bmjopen-2022-066757

**Published:** 2023-05-02

**Authors:** Richard S Bourne, Mark Jeffries, Denham L Phipps, Jennifer K Jennings, Emma Boxall, Franki Wilson, Helen March, Darren M Ashcroft

**Affiliations:** 1 Department of Pharmacy, Sheffield Teaching Hospitals NHS Foundation Trust, Sheffield, UK; 2 Division of Pharmacy and Optometry, School of Health Sciences, The University of Manchester, Manchester, UK; 3 NIHR Greater Manchester Patient Safety Translational Research Centre, Manchester, UK; 4 Department of Pharmacy, Salford Royal Hospital, Northern Care Alliance NHS Foundation Trust, Salford, UK; 5 Department of Pharmacy, Leeds Teaching Hospitals NHS Trust, Leeds, UK; 6 Department of Pharmacy, Royal Oldham Hospital, Northern Care Alliance NHS Foundation Trust, Oldham, UK

**Keywords:** adult intensive & critical care, health & safety, quality in health care, qualitative research

## Abstract

**Objective:**

To understand the sociotechnical factors affecting medication safety when intensive care patients are transferred to a hospital ward. Consideration of these medication safety factors would provide a theoretical basis, on which future interventions can be developed and evaluated to improve patient care.

**Design:**

Qualitative study using semistructured interviews of intensive care and hospital ward-based healthcare professionals. Transcripts were anonymised prior to thematic analysis using the London Protocol and Systems Engineering in Patient Safety V.3.0 model frameworks.

**Setting:**

Four north of England National Health Service hospitals. All hospitals used electronic prescribing in intensive care and hospital ward settings.

**Participants:**

Intensive care and hospital ward healthcare professionals (intensive care medical staff, advanced practitioners, pharmacists and outreach team members; ward-based medical staff and clinical pharmacists).

**Results:**

Twenty-two healthcare professionals were interviewed. We identified 13 factors within five broad themes, describing the interactions that most strongly influenced the performance of the intensive care to hospital ward system interface. The themes were: Complexity of process performance and interactions; Time pressures and considerations; Communication processes and challenges; Technology and systems and Beliefs about consequences for the patient and organisation.

**Conclusions:**

The complexity of the interactions on the system performance and time dependency was clear. We make several recommendations for policy change and further research based on improving: availability of hospital-wide integrated and functional electronic prescribing systems, patient flow systems, sufficient multiprofessional critical care staffing, knowledge and skills of staff, team performance, communication and collaboration and patient and family engagement.

**STRENGTHS AND LIMITATIONS OF THIS STUDY**

- Medication safety for intensive care patients transitioning to a hospital ward requires co-ordination of care within and across multiprofessional teams and environments with varying staff and technological resources.
- This is the first qualitative study focused on understanding the sociotechnical factors of medication safety in patients transitioning from intensive care to a hospital ward.
- Study strengths include multicentre views of intensive care and hospital ward UK healthcare professionals, representing teaching and district general hospitals, all using electronic prescribing systems.
- Thematic analysis using the Systems Engineering Initiative for Patient Safety V.3.0 model enabled evaluation of the sociotechnical factors impacting on medication safety during transition from intensive care to a hospital ward.
- We report on findings from four trusts from England, and findings can not be generalised to other settings outside the UK.

## Background

Patients recovering from a critical illness requiring intensive care unit (ICU) treatment are generally transferred to a hospital ward before eventual hospital discharge to the community. However, this simplified summary under-represents the complexity of the patient pathway, where full patient recovery may be protracted, traversing hospital and community care and involve several care transitions.^1 2^ Compared with patients discharged from standard hospital care, ICU patients have worse short and medium-term outcomes, requiring additional ongoing healthcare resources.^3^ Half of ICU patients require emergency hospital readmission within a year of hospital discharge.^4^ This hospital readmission risk is predicted by pre-existing patient multimorbidity, frailty and polypharmacy.^4 5^ There is a growing appreciation of the need to improve the recovery experience of ICU survivors, including reducing complications related to polypharmacy and care fragmentation.^1 6^ Transitions in care are known to increase patient risks, including those related to medicines usage and care continuity.^7^ The transition from ICU to a hospital ward is a marked interface in patient care, with contrasting resources in care team and technological support available in the respective clinical areas. Transition from ICU exposes patients to high rates of medication errors (MEs),^8–10^ and adverse events are common, including those that are medication related.^11^ Extensive and frequent medication changes are made during a patient’s ICU episode,^12 13^ increasing the risks for hazardous polypharmacy on care transitions.^14^ The most common MEs centre around failure to discontinue inappropriate acute medication without an ongoing indication and not restarting clinically important long-term medication when safe to do so.^8–10 15^ Such MEs may remain uncorrected beyond the acute hospital care episode^16–19^ and be potentiated by pre-existing polypharmacy.^20^ ICU care may, therefore, compound the known complexities and challenges for patient medication care created by care transitions.^7 21^

22 Organisational recommendations to support the continuity of care, including mitigation of these increased ME risks, have centred around structured staff handovers between ICU and the hospital ward to support the continuity of care including a focus on medication.^23^ Broader indicators of interventions that can improve medication safety on transitions in patient care are provided by the WHO and ICU expert views.^7 24^ To date, interventions to improve the safety and continuity of medication for ICU patients transitioning to a hospital ward have focused on education of staff, medication review, guidelines, electronic transfer/handover tool or letter and medicines reconciliation.^25^ Simple interventions, based on staff education and guidelines, have been demonstrated to reduce the risk of inappropriate continuation of acute medication no longer indicated.^25^ The more complex, and higher clinic risk, of failure to restart clinically important long-term medication, will require a more complex intervention package.^25–27^

For any intervention package to be effective in the ICU environment and improve patient care, it is important to understand how the ICU system works.^28^ Furthermore, the care interfaces need to be examined in terms of their systems, processes and outcomes.^29^ Error prevention in ICU care is dependent on effective inter and intramultidisciplinary team working and communication,^30^ which are particularly important on patient transition from ICU to the hospital ward.^31 32^ Despite ICUs being technology rich environments, an over-reliance on technology to mitigate against MEs underestimates the complexity of the systems and processes.^33 34^ Like other parts of healthcare, ICU and its care transitions involve a complex interplay between technology, tasks and people.^35^ Therefore, the importance of human factors engineering has been promoted to address the whole ICU medication management and patient safety pathway.^30 36 37^

This study aimed to understand the sociotechnical factors affecting medication safety when ICU patients are transferred to a hospital ward. Consideration of these medication safety factors would provide a theoretical basis, on which future interventions can be developed and evaluated to improve patient recovery.

## Methods

Our study used a qualitative design, intended to solicit adult, general ICU and ward-based healthcare professionals directly involved in the prescribing and medication review of ICU patients on the interface of transition to the hospital ward. We recruited from a purposive sample of healthcare professionals across four National Health Service (NHS) centres, chosen to represent both District General and Teaching Hospitals in the north of England with a representative range and combination of ICU and hospital ward e-prescribing systems (online supplemental table S1). The sampling frame was ICU (medical staff, advanced critical care practitioners (ACCPs), critical care pharmacists and ICU outreach team members) and ward-based (medical staff, clinical pharmacists) healthcare professionals. Principal investigators at each NHS hospital directed participant email invitations via departmental team leads or departmental email distribution lists. All participants provided informed written consent.

10.1136/bmjopen-2022-066757.supp1 Supplementary data

We undertook semistructured interviews facilitated by a topic guide adapted from the London Protocol^38^ and Systems Engineering Initiative for Patient Safety (SEIPS) V.3.0 model^29^ (online supplemental file, Topic guide). The London Protocol was developed from Reason’s ‘Swiss Cheese’ model, capturing the contributing factors and context around a specific patient safety incident.^38^ The SEIPS models are based on the System-Process-Outcome description of approaches to measuring and improving the quality of healthcare. V3.0 of the SEIPS framework includes an expanded process component, focusing on the patient pathway to describe patient interactions with multiple care settings over time.^29^ To prompt discussion, we selected three reported ICU ME cases as vignettes, each representing a type of human error from Reason’s (1990) generic error modelling system^39^ (see online supplemental table S2). The vignettes were shared with participants in advance of the scheduled interview. All interviews were undertaken via video telecommunication software (Microsoft Teams, Microsoft Corporation), recorded and transcribed verbatim. A qualitative researcher (MJ) experienced in investigation of medicines optimisation practices in NHS healthcare environments conducted the interviews. Transcripts were anonymised prior to deductive thematic analysis,^40^ using the London Protocol,^38^ and SEIPS V.3.0 model^29^ frameworks. Coding was supported through qualitative research software (NVivo, V.12, QSR International (UK) Limited) and undertaken in duplicate by the qualitative researcher (MJ) and chief investigator (RSB). All transcript contents were coded using the thematic analysis. The coding was completed in three rounds with discussions between the coders after each round, and with the wider research team at different intervals. Data saturation was considered to be achieved when we judged further interpretation of codes and themes would not provide further insights.^41^ Finally, through multiple discussions among the research team, we coalesced the main identified factors until they provided a stable and parsimonious account of the interactions that most strongly influence the performance of the ICU to hospital ward work system and communication pathways.

We have actively engaged with patient and public involvement (PPI) in this wider research intervention development programme, to improve medication safety for intensive care patients transitioning to a hospital ward. In this study, PPI colleagues consulted on and contributed to the development of the topic guide, interpretation of the thematic findings and dissemination of results.

### Ethics

Ethical (The University of Manchester, Ref: 2020-10852-17342) and NHS Health Research Authority (HRA) (IRAS 292456) approval was granted for the study. We have adhered to the Consolidated Criteria for Reporting Qualitative Studies (COREQ) in reporting our findings.^42^

## Results

We recruited 22 participants representing each professional group and clinical location (see table 1 for more details).^41^

**Table 1:** Healthcare professional participants (n=22)

Healthcare professional staff group	ICU medical staff	Advanced Critical Care Practitioners (ACCP)	Critical care pharmacists	Outreach team members	Ward medical staff	Ward clinical pharmacists
n	3*	4	4	4	3*	4
Time in current role (years, mean (SD))	2.7 (2.1)	6 (2.2)	11 (12.8)	12.3 (5.5)	2 (1)	1.5 (1.3)

*Participants from sites 1, 3 and 4 only.

ICU, intensive care unit.

**Figure 1:**
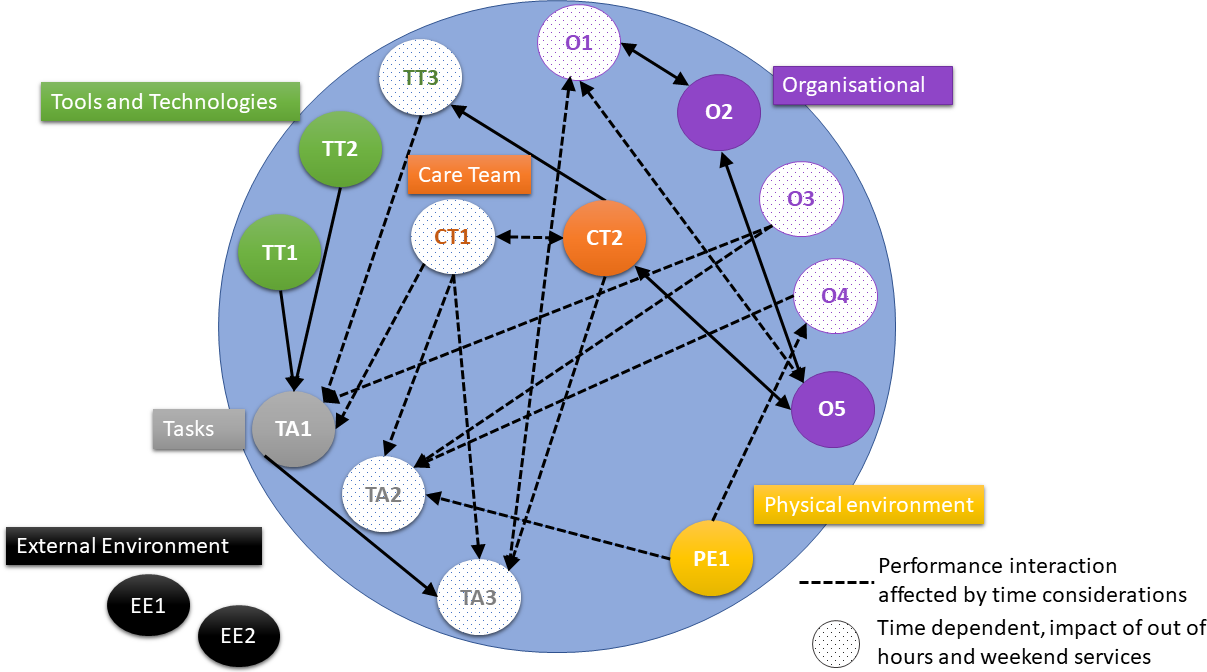
ICU patient transfer performance interaction. CT, care team factor; EE, external environment factor; ICU, intensive care unit; O, organisational factor; PE, physical environment factor; TA, task factor; TT, tools and technology factor.

**Figure 2:**
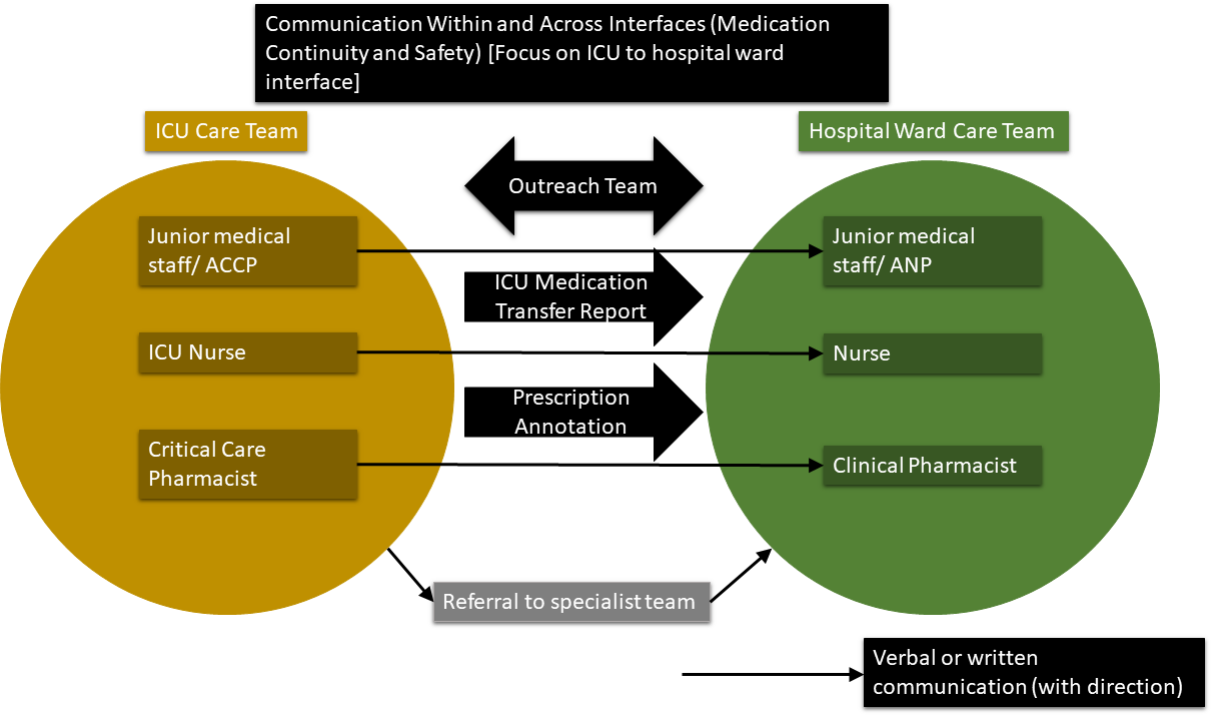
Medication continuity and safety communication across ICU to hospital ward interface. ACCP, advanced critical care practitioner; ANP, advanced nurse practitioner; ICU, intensive care unit.

We identified 13 factors within five broad themes, providing a detailed description of the ICU–hospital work–system interface. This identified the complexity and interactions within the system and accounted for the significant impact of time pressures and considerations (box 1, figure 1). The vulnerability of the system to time constraints was evident (figure 1). The communication pathways used are presented (figure 2).

Box 1 **ICU–hospital ward work–system interface**

*ICU active agents*: ICU consultant, ICU junior medical staff, advanced critical care practitioner, critical care pharmacist, ICU nurse, outreach team, microbiologist.

*Hospital ward active agents*: junior medical staff, specialist teams (eg, diabetes team, acute pain team), clinical pharmacist, nurse, outreach team.

**Performance is shaped most *strongly* by interactions of:**

*Care Team Factor(s*)

- CT1: Knowledge and skills of staff on medication review and safe prescribing for ICU patients on the interface of transition to the hospital ward* [(Effect dependent on status])
- CT2: Beliefs about consequences, understanding of staff to the frequency and clinical impact of MEs on patient recovery* [(Positive effect])

*Task Factor(s*)

- TA1: Task and patient complexity affected by the number of long-term and acute medicines used in patients with a critical illness with unpredictable recovery and responses to treatment including medication [(Negative effect])
- TA2: Task prioritisation, often focused on the new patient admission(s) or more unstable patient(s) in ICU, with medication use in ICU transfer patient deemed low priority* [(Negative effect])
- TA3: Task allocation and delegation among the care team* [(Effect dependent on status])

*Tools and Technologies Factor(s*)

- TT1: Electronic prescribing system integration and functionality** [(Effect dependent on status])
- TT2: Electronic patient notes accessibility and searchability** [(Effect dependent on status])
- TT3: Medication transfer checklist completion and adherence* [(Effect dependent on status])

*Organisational Factor(s*)

- O1: Team performance (organisation, leadership, collaboration and dependability)* [(Effect dependent on status])
- O2: Multiprofessional ward rounds including a medication review element* [(Positive effect])
- O3: Communication between ICU and Hospital Ward team members*/** [(Effect dependent on status])
- O4: Time pressures in the need to complete the ICU patient transfer to the ward, staff workload and out of hours transfers*/** [(Negative effect])
- O5: Safety culture in ICU informed by incident reviews, audit and feedback and follow upfollow-up clinic experiences* [(Positive effect])

*Physical Environment Factor(s*)

- PE1: ICU bed availability and pressure for beds** [(Effect dependent on status])

*External Environment Factor(s*)

- EE1: Bed availability on the hospital ward and transfer times** [(Effect dependent on status])
- EE2: Transfer time standards [(Negative effect])

On ICU, Patient and Family co-agents*, also Dietitian, Nutrition support team, Visiting teams and Pharmacy Technician. On the Hospital Ward, the Rehabilitation Team were co-agents.

*Potentially modifiable at a local ICU level; **potentially modifiable at an organisational level.

### Theme 1. Complexity of process performance and interactions

#### Care teams and collaboration

A professionally diverse range of ICU and hospital ward-based healthcare staff undertook important tasks and processes to support medication safety for ICU patients transferring to a hospital ward (box 1). In the ICU, participants emphasised the value of collaborative, multiprofessional team working that was driven by effective team leadership. ICU consultants led multiprofessional ward rounds, provided leadership and examples for more junior staff in the routine delivery of patient medication reviews. ACCP, medical and nursing staff had important roles in medication review. The role of the critical care pharmacist in medicines reconciliation on admission and undertaking medication reviews for patients, including during ward rounds and prior to hospital discharge, was highlighted.

the MDT ward round is really useful, as the […] consultant you’re trying to pull a broad picture of everything together for discharge, and medications is one part of that, and I think we’re very lucky in (name of hospital), the quality and the provision of our pharmacy support is excellent. […] I don’t see how we could do it without the pharmacists (P5 ICU consultant).

On the hospital ward, there was also evidence of multiprofessional team working in undertaking medication reviews in ICU patients transferred to the hospital ward. In some cases, these were facilitated by consultant-led ward rounds. While direct collaboration between the ICU and hospital ward team appeared less clear, the ICU outreach team did provide an important link between the two clinical areas.

So I follow patients up post-discharge from intensive care […] to look at them physiologically, psychologically, and just follow the plan that’s been put in place on a critical care area. […] Regarding medication, if there’s something specific in the discharge plan, then I would review that aspect (P1 outreach team).

On the hospital ward, referral to clinical pharmacists and specialist teams in undertaking medication reviews were highlighted. The diabetes team was seen as essential for patients receiving insulin therapy when transferred to a hospital ward and the acute pain team was often engaged with patients with ongoing pain issues.

But there’s also other specialists that we get involved […] we should automatically make a diabetic team referral. So, it’s also pulling in other specialist teams into the mix […] (P3 ACCP).

#### Tasks, processes and prioritisation

The key medication-related tasks undertaken by ICU healthcare professionals interviewed were medication review, prescribing and plan setting. These were supported by processes such as medicines reconciliation and, in some units, a transfer checklist that included medication elements. ICU transfer reports sometimes included medication information such as preadmission medicines, current medication and medication plans. Transcribing and transcription checks were also undertaken in ICUs without integrated ICU and hospital ward electronic prescribing systems, which also provided a medication review opportunity.

I would say [the transcription process is] vitally important. So, part of the problem is […] it shouldn’t really be seen as a transcription process, it should be more of a review process. […] it’s not simply passing straight from one system to another including all that information, it’s actually reviewing what’s appropriate (P2 critical care pharmacist).

The complexity of tasks undertaken was qualified within the context of ICU patient care and regarded as impacting on medication safety throughout the patient journey. The acuity of the patient condition, and increasing number of medicines they received, contributed to medication review complexity.

we’re now seeing patients who are more complex than ever before and on more medicines than ever before, and then, obviously, that becomes even more complex in itself (P9 ICU consultant).

Tasks were often prioritised, which sometimes meant that the medication-related tasks were seen as a low or lower priority and ‘probably the least important thing you would do that day (P13 ward junior doctor).

The seriousness of the patient’s condition determined the time spent on their care; more seriously ill patients were prioritised over the patient transferring from ICU.

And because it’s quite a fast-paced unit those patients could be gone before I see them again because then I could be reviewing the critical unwell patients, the Level 3 patients, the ones that are intubated, and things. So, those patients that are then getting discharged are in the back of my mind because I'm dealing with the sicker ones (P14 ACCP).

The priority for ward-based clinical pharmacists was often focused on undertaking medicines reconciliation for new hospital admissions and discharge prescriptions, over reviewing patients transferred from ICU, while ward medical staff focused on critical medicines.

So I think when you're on the ward round and you go to review the medicines, you mainly look at things that are critical to the patient at the time first. So antibiotics, blood thinners and that sort of thing. And looking back to admission meds to see what’s missing while they’re still in hospital is not always top of your list (P20 ward junior doctor).

#### Task allocation and delegation

Task allocation and task delegation were important considerations. Participants had differed in how they rated the importance of medication-related tasks. Among medical staff, transfer planning around medication was often allocated to a junior team member and this was seen as a ‘low-level’ task, whereas critical care pharmacists and ACCPs considered these more complex tasks.

A lot of them [junior doctors] will just write, see [electronic prescription], and that’s it. They aren't actually doing a formal review of the meds. […] it’s their first job in critical care, and they can just get lumbered to do these discharge summaries because they are a straightforward thing to do […] They maybe don’t go into the complexities that they should do in some of them (P14 ACCP).

Moreover, when it came to medication review on ICU patient transfer, staff groups did not always seem aware of the roles and responsibilities of other staff groups.

In terms of when they step down to the ward, I don't know what role our ICU pharmacists have there. I'm not sure I've actually known they've had any active role in that. I think they would flag if they're still on a medication that can't be given or they think their role is much more with new patients and new prescriptions in ICU. I'm sure ward pharmacists, when they go to the ward, …I'm much more imagining it’s their role to review the new prescriptions coming to the ward (P15 ICU junior doctor).

ICU participants suggested responsibility for medication review lay with the hospital ward team or general practitioner and that ‘*someone else will do it’* (*P9 ICU consultant*).

It was suggested that a specific staff group or groups needed to take ownership of the medication review prior to the patients’ ICU to hospital ward transfer. The ACCP or critical care pharmacist were considered the most appropriate staff groups to do this. Both these staff groups had the benefit of service stability over the frequent rotation of junior medical staff.

#### Knowledge, skills and professional diversity

Different staff groups were felt to have different knowledge and skills, for example, medical staff diagnostic and treatment planning skills versus pharmacist medication planning and prescribing skills.

I've got to say that the pharmacists and doctors, I would say the pharmacists within critical care are really good at identifying drugs that’s been withheld and putting it in their plan. And then the doctors have got good at looking at the pharmacy plan and pulling it through into the final discharge report (P1 outreach team).

ACCPs were thought to have knowledge and skills in medication reviews through having *done the process hundreds of times* (*P5 ICU consultant*), in comparison to consultants and junior doctors.

On the hospital ward, there appeared a hesitancy to review medication that had been commenced while the patient was in ICU. This seemed to stem from a lack of familiarity with ICU medication and a feeling that the ICU team knew what they were doing. This was compounded by lack of knowledge and skills of junior medical and pharmacy staff meaning that acute medications could continue longer term.

I think it’s… to get an effective medication review, I think it depends on the pharmacist and their experience, which has been the key thing. So I think if you’ve got really junior staff doing a medication review on a patient that’s stepped down from ICU, I would imagine they’d find it difficult, […]. And I feel like they would be more reluctant to make medication changes or recommendations to doctors (P19 ward clinical pharmacist).

### Theme 2. Time pressures and considerations

#### Time pressures

Time pressures and considerations affected the system performance (figure 1). Time pressures contributed to staff not always completing information such as the rationale for prescribing, pausing or deprescribing medication.

…a doctor changes a medicine or stops a medicine, then when you do it on the [e-prescribing system], you’re supposed to fill in a box to say what you’ve done […] (people are) basically doing this as quite a quick exercise, they’ll just go, click, click, click and then they bypass that very often. And we all do it …(P11 critical care pharmacist)

Time pressures around the ICU transfer process contributed to some patients being transferred without the opportunity to undertake a prior medication review. Time pressures could mean that staff handovers did not occur and transfer reports and medication plans were not completed.

I think there is often a lot of time pressure on the discharge planning process and that would definitely make a slip type error, because you’re so focused on getting the task done, you do what is necessary and then this is probably not absolutely necessary to get the patient discharged and complete your task it’s an additional thing isn’t it? What you’re trying to do is get the patient out onto the ward with everything they need, rather than what else? (P5 ICU consultant).

#### Workload and staffing levels

One barrier to completing medication reviews was in the healthcare staff-patient ratio particularly on the hospital ward.

It’s predominantly the number of patients to see. And also slightly lack of staff on the wards as well. Because if you just have one consultant and one junior, they'll be trying to look things up on the computer, get the meds up, talk to the consultant, see the patient, and then not always have time to then sit and do the prescription at the same time (P20 ward junior doctor).

The benefits of clinical pharmacist medication review were acknowledged to be hampered by reduced dependability borne out of insufficient staffing, lack of 7-day services and set daytime working patterns.

I think the biggest constraint in the system is provision of clinical pharmacy staff. […] actually having the pharmacists time available to undertake those medicines safety checks is the core part of business. And there’s only a few of them, […] there’s certain things that we as clinicians can do to mitigate that to a degree. […] well the root cause of the problem is […] insufficient pharmacy staff time (P9 ICU consultant).

#### Out of hours discharges

Out of hours transfer of patients from ICU to a hospital ward were regarded as particularly high risk for MEs.

…. particularly in out of hours, unfortunately, just because of bed availabilities and pressures, that can be quite challenging in terms of information being passed from one team to the next. So information is lost almost about new medications that are commenced for various reasons on the ICU (P21 ACCP).

Discharges out of hours were seen as more problematic because of reduced ICU and hospital ward staffing levels, particularly with the unavailability of wider specialist teams and fewer senior healthcare staff available. It was felt that daytime discharges were conducted better, allowed *the pharmacists (to) provide a review of the discharge* (*P5 ICU consultant*) and increased the likelihood of multiprofessional and senior medical staff review. Sometimes out of hours transfers were unavoidable when ICUs were full and acute admissions to the unit were required for patient flow.

There are a lot of our patients that are discharged out of hours, after 6:00 pm, and that shouldn’t be happening. Our patients should be out in a timely manner, sat on a ward, safety, so people have time to make sure everything’s documented and sorted for that patient before the nightshift […] (P14 ACCP).

#### Bed availability and service standards

Patient flow challenges created pressures on ICU and hospital bed availability, which in turn created issues for patient transfer planning and review. The pressure to get a patient discharged within specified service standards was identified. Transfers could be rushed with short notice of ward bed availability.

…it could be that a patient’s on ICU, they’ve been ready to go to the ward for four or five days, there’s not been a bed available for them. And then we’ve got say three poorly patients out on the wards in A&E that urgently need a bed. So it’s sort of a rapid discharge then to clear the beds (P7 outreach team).

### Theme 3. Communication processes and challenges

Effective communication was seen as a key safety component in transition of patient care *working together with good communication* (*P1 outreach team*). Conveyance of accurate and complete medication information (eg, medication changes) on patient transfer was felt important overall. Several challenges to effective communication were apparent. First, the considerable number of healthcare staff involved in information transfer was seen as a barrier to effective communication. Pertinent information could get lost in the sheer volume of exhaustive patient notes after an ICU care episode, creating an accessibility barrier. Barriers to information accessibility, and in the case of clinical pharmacists meant they reviewed fewer patients. Finally, information was not always communicated, and staff would sometimes forget to include key details. Several systems were employed to improve communication of information including medication, principally handovers, transfer summaries and prescription annotations.

#### Handovers (ICU to hospital ward)

Staff used handover processes to convey information on transitions of patient care. Handovers were verbal, written or combinations thereof (figure 2). ICU medical, nursing and pharmacy staff had handover processes to their respective hospital ward colleagues. Face-to-face handovers were seen by some as being the best method, while transfer of verbal information around shift changes or out of hours were considered higher risk for being lost in the system. Medical, nursing and pharmacy staff had limited awareness of what each other handed over and how this was undertaken. This contributed to assumptions or uncertainties that other staff groups were handing this information over. There was some uncertainty over what information to convey about medication on transitions of care. Medical staff tended to only handover information that they perceived to be important, that needed to be followed up related to the key patient problems, for example, a scan needs to be requested. It was often unclear if medication details would be included or not, however, this information was often considered of low interest for medical staff.

I don't think the doctors, […] have a verbal handover. […] everything’s documented on this transfer medication report and so it’s at their discretion for them to read it and to make sure that everything’s handed over. […] But in terms of, like, a verbal handover I know that doesn’t happen (P18 ward clinical pharmacist).

#### Transfer summary

The transfer summary was considered the communication focus, providing an effective means of highlighting and collating essential information and care plans and considered more dependable than verbal handovers. When completed well, the transfer summary was used to convey information and prompt medication reviews.

So within that there are fields specifying medication comments and actions, ongoing actions for the ward. So we try to put into that the information, so it doesn't get lost and that’s the way to try and improve that safety and that communication around medications. But if they become well to step out and then there’s bed pressures and they don't step for a day or two that information can almost get lost within all the notes (P21 ACCP).

Furthermore, having the transfer summary easily accessible electronically by all healthcare professional was deemed important since *everyone knows it’s there […] the receiving team can read that’* (P7 outreach team).

#### Prescription annotations

Annotations on the electronic prescription were seen as an effective way of maintaining focused, easily accessible information regarding medication. Prescribing important chronic medication and then holding or suspending it on the electronic prescribing system was seen as a prompt for medication review. However, annotated information was often omitted, particularly if transcription was required.

So, I think when it’s done properly and it’s suspended and… Our [e-prescribing system] is great, you can write all sorts of qualifiers, indicators, you can write all sorts on there as a note for future teams. And I think that’s when I think it makes most sense. And if I'm receiving a patient and someone’s written a note to me, it’s just pretty obvious what I've got to do. That all works well. I think when people just leave them off or stop them, but don't write reasons. When you stop any medication there is a comment to cease it and often that’s not filled in either (P15 ICU junior doctor).

### Theme 4. Technology and systems

Technology was used to prescribe medication, record and store information about patients’ medicines, prioritise workload and to facilitate communication with other healthcare professionals. Overall, electronic prescribing was felt to have safety benefits over paper prescription and charts with prescription defaults, order sets, improved decision support and prompts and accessibility to information such as medicines reconciliation. Electronic (e-)prescribing also made information retrieval about medication changes easier to undertake, aiding medication review.

Technology also supported the use of medication transfer checklists and had further potential application via automation of some processes such as information pooling and patient prioritisation.

There’s also an inbuilt checklist into the critical care system, so when a patient’s being discharged, the doctor is supposed to complete all the discharge information. And before they can do that there is a checklist which they are supposed to tick off which says, I have reviewed all the patient’s usual medicines, I have reviewed all the current medicines to make sure that nothing’s inappropriately being continued,… (P2 critical care pharmacist).

However, it was noted that over time checklists became less effective as staff fatigue with the system and find workarounds. Although all ICUs used e-prescribing, there was some requirement on specific ICUs to use paper prescriptions for some medicines (eg, intravenous medicines and fluids). One centre had separate ICU and ward-based systems that did not communicate directly, creating an electronic transcription phase to ICU patient transitions. Participants were clear on the importance of full integration of e-prescribing systems, so that all medications were fully visible and auditable in a single format and transcription was not required.

It would say be better if everything was electronic and go to the ward electronic, because things get misfiled, don’t they? […] Lost. If it was all electronic and got sent electronically, then that would be the safest way. I think often with prescribing problems that can be a problem, when some things are electronic and some things are paper, then it gets very confusing, doesn’t it? (P12 ACCP).

System functionality was emphasised in minimising the number of phases or menus required in the e-prescribing system to prescribe, hold and plan reviews of medication. Limitations in functionality disinclined staff to prescribe medication not immediately required, which compromised the identified communication benefits of prescribing and holding a potentially important long-term medication requiring regular review for restarting when clinically appropriate. The importance of accessibility was identified when it came to finding required information quickly, minimising loss of information within menu caches.

Over-reliance on the individual to undertake tasks increased the risk of them not being done.

I think, I think a lot of the errors occur because it’s down to individuals to do things. And you can put systems in place, and you can put protocols in place, but people will always find a way around them, when you’re busy you’ll always take the easiest route.[…] Whereas, if you had ways of connecting up systems so that it’s automatically done, then it’s less reliant on the individuals. The individuals would still need to be involved in it and can still up on errors and add to the system, but you would probably get fewer errors, certainly less, sort of, information loss if the systems talked to each other (P2 critical care pharmacist).

### Theme 5. Beliefs about consequences for the patient and organisation

#### MEs and adverse patient outcomes

MEs reported included inappropriate continuation of medication started in ICU without an ongoing indication, loss of important medication information (eg, review plan) and failure to restart potentially important chronic medication. Although participants acknowledged such MEs were common, the outcomes for patients and the impact on them were not something that participants seemed to universally appreciate or take into consideration.

I've never really thought about that [potential impact on patients]. I would hope not. I would hope by the point we get to discharge the wards have reviewed all the medications properly and done a decent [discharge prescription] […]. So, they're generally on medication short term to try and help psychosis, which I would hope gets stopped before they get discharged because it’s not something we ask to be on long term, but I don't know if I'm honest. It’s not something that we follow up on (P14 ACCP).

Participants identified that the implications would vary depending on certain factors such as the specific ME and timescale considered and that there was a *very wide range of possible outcomes of omitting medicines, unintentionally or intentionally* (*P10 ward clinical pharmacist*).

Some participants considered the potential for adverse effects as a longer term risk and would not be apparent until sometime after the hospital discharge (eg, unintentional discontinuation of secondary prevention medication postmyocardial infarction or in the case below of continuation of antipsychotic medication).

We had […] a case about ten years ago where a patient was prescribed three different medicines for delirium, different psychiatric medicines while they were on unit, then discharged to the ward, went home and then eventually they went back to their GP, was asked to sign their repeat prescription three months later and he looked at it and said, I didn’t know this patient was under psychiatric care. And then looked into it and they’d basically never been stopped, the patient went home on anti-psychotic medicines, nobody stopped him (P11 critical care pharmacist).

Units that had undertaken a review on incidents, clinical audits or operated follow-up clinics appeared more cognisant of potential medication issues after ICU patient transfer and implications for patient recovery. Some participants reported unit follow-up of clinical incidents related to adverse drug events had been undertaken. Others had undertaken clinical audits or service evaluations of medication continuity and safety, which increased staff awareness of the high rates of MEs on transition from ICU.

Yeah, ‘cause we have had a couple of incidents and we’ve learned from them, and in fact our pharmacist did an audit on it, and presented that audit to the MDT, and there was a lot of learning points from that, and it has brought to us the importance, ‘cause I think people just didn’t, it just wasn’t very high up in people’s concerns about a patient (P12 ACCP).

Feedback from follow-up clinics were another way staff learnt about MEs on transition and patient adverse drug events. These safety culture processes reduced the chance of siloed ICU perspectives on the patient care pathway.

#### Patient and family engagement

Participants identified that ICU patients frequently experienced delirium and memory loss, which was a challenge to potential engagement in medication discussions and information while in ICU. Concern about lack of patient recollection was a barrier to healthcare professional communication around medication handover on planned transfer to the hospital ward. It was unclear what perceived role family members had in this scenario. While in ICU, the care team tended to take over all control of patient medication.

…we take complete control of their medications, nines time out of ten in hospital, so we are, you know, we are basically putting it all in our hands, and responsibility therefore. (P9 ICU consultant).

Patient involvement in their medication through guiding discussions and questioning changes was highlighted when their condition improved. Consequently, the role of the patient and family in medication safety and continuity while in ICU appeared passive, rather than being active agents.

And usually you find that a patient who’s got long term illness, they're usually quite good at questioning medication. I can foresee that patients on intensive care, they’ve got poor concentration, attention, and they'll be coming to that remit of, the nurse […] gave me six tablets, I'll just take them without asking any questions, et cetera (P1 outreach team).

## Discussion

This is the first qualitative study focused on understanding the sociotechnical factors of medication safety in patients transitioning from ICU. We identified 13 factors within five broad themes, providing an interactive description of the factors that most strongly influenced the system performance. By highlighting the important system factors at an ICU and wider organisational levels, we provide a focus for service improvement efforts. Notably, time pressure considerations (workload, staffing, shift work, out of hours transfers, bed availability) have a marked negative effect on the system performance. We now discuss how these factors impacted on team and perceived system performance in relation to the key socio-organisational and work system components identified.^29^

### Care team factors

We saw diverse multiprofessional staff groups, working in ICU and hospital-ward teams, were agents in the delivery of medication continuity and safety on ICU patient transfer. The performance of care teams was affected by staff knowledge and skills in medication review and prescribing, being perceived to vary across healthcare professional groups. Here, the core stability of some ICU staff groups benefited their practice and experience, over staff with regular clinical rotations. Importantly, limitations in understanding of the potential impact of MEs on patient or organisation outcomes undermined the meaning of mitigating tasks or processes undertaken, particularly among rotational staff groups. These limitations in ICU and ward-based staff beliefs about consequences of MEs on this care interface present a challenge to the success of future behaviour change interventions.^43^

We identified that the patient and family were often considered coagents by ICU staff, reducing their engagement. Barriers to agent status included delirium and memory loss for patients. These findings may explain some of the limited patient awareness of medication changes identified on follow-up.^18^ Nevertheless, lack of communication and family support may also be factors.^2^ Engaging patients and family members around safety of medication in transitions in care is recommended,^7^ and there are opportunities, such as written communication,^44^ to support the resilience and adaptability exhibited by patients and family in recovery^2^ and care satisfaction.^32^

### Task factors

The logistical challenges of patient flow on the transition from ICU to the hospital ward are well known.^32 45^ We show that medication safety for ICU patients transitioning to a hospital ward is a complex process, involving complex patients, following an uncertain care pathway, involving diverse multiprofessional healthcare staff groups and personnel organised in multiple teams with varying resources. Process complexity was further increased by being time critical and dependent. Task prioritisation was focused on the more acutely ill patients in ICU, or new patients on the hospital ward, potentially affected by staff resources and physical environment factors (ICU bed availability and pressures for beds).^46^ We found some medical staff considered the medication elements were a low priority to include in ICU patient transfer and handover plans, as previously reported by others.^32^ In contrast, several medication-related elements (preadmission medicines, active medications on transfer, rationale for medication regime and intravenous infusions) were considered essential information elements by ICU and ward-based Canadian medical staff.^47^

### Tools and technologies factors

We report that the full integration of e-prescribing systems across ICU and the hospital ward was clearly important in reducing medication transcription errors. Progression from non-integrated to integrated ICU and hospital ward e-prescribing systems reduced the frequency and clinical severity of MEs on transition in ICU patient care in an Australian report.^48^ Although, it was also apparent that represcribing on transfer provided an opportunity to prompt a structured medication review, previously being associated with reduced risk of medication transfer errors.^10^ Communication of information was viewed as supported when it was easily accessible and searchable within the numerous and complicated electronic system menus. The structure and content of the medication-related communication was aided by transfer checklists and summary reports system functionality.

### Organisational factors

ICU leadership was provided by the ICU consultant and it was apparent from ICU participants that team performance, as exemplified in multiprofessional ward rounds, was supported by psychological safety. This is reassuring given that ward round perception on quality of leadership predicts open multiprofessional communication within ICU.^49^ Indeed, ICUs with a multiprofessional ward round are associated with a marked reduction in medication transfer errors.^10^ However, ICU team performance is also informed by the composition and interaction of the multiprofessional healthcare team, providing diversity of specialism working on a co-ordinated and goal orientated manner.^50^ Although we identified clear professional diversity, there were also indications of opportunities to further improve team effectiveness and performance by strengthening other components (dependability, structure and clarity, meaning, impact).^51^

Communication, particularly inter-team (ICU to Ward) communication, was considered a challenge to medication safety and continuity. These staff communication challenges around patient transfer from ICU to a hospital ward are well described elsewhere.^32 52 53^ Limitations in written communication content are common, lacking decision-making information such as the rationale for medications that were started or stopped.^53^ Lack of communication about medication may contribute to MEs in ICU survivors on the hospital ward,^52^ and inadequate handover can contribute to the distress patients experience on this transition in care interface.^52^ We identified several factors that facilitated communication of medication related information. These included multiprofessional handovers, prescription annotations, outreach teams following up patients and plans, and referrals to specialist teams. Previous systematic reviews^31 54^ have identified the importance of handover forms and liaison nurses, improving continuity or care but without any evidence of improved patient outcomes. However, it is not enough that a handover occurred, but that the right information is conveyed, acknowledged and actioned. Automation of facets of medication-related information via technological systems improves information transfer and delivery on ICU patient transitions.^55^

Time pressures and considerations affected organisational conditions with time constraints having a notable effect on system performance. The important impact of such time considerations as facilitators and barriers to care during discharge from the ICU have been recently summarised.^56^ Patients transferred out of hours from ICUs have worse outcomes,^57^ and it is conceivable that medication safety issues contribute to this increased risk.

Our results share consistent themes previously identified in ICU transfers and medication or care continuity. Lack of staff knowledge, unclear task allocation and staff collaboration are barriers to medication safety on transitions in patient care, as reported for medicines reconciliation processes.^58^ Variation and lack of clarity of roles around inpatient medication reviews have previously been identified as an additional risk factor for MEs, as reported for ward-based clinical pharmacists.^59^ Patient severity (complexity) of illness, provider experience (knowledge and skills) and resource constraints (workload and staff availability) have previously been identified as themes affecting ICU patient discharge and readmission risk.^60^

### Strengths and limitations

Our study had several strengths. Our findings are strengthened by the inclusion of views of UK healthcare professionals from both the ICU (transferring) and the hospital ward (receiving) multicentre teams, involved in the medication review and prescribing of ICU patients. Views represented teaching and district general hospitals, all of which use e-prescribing systems. Thematic analysis was framed on the SEIPS 3.0 model,^29^ which was developed specifically for evaluating the human factors of patient safety during transitions in care. We provide system interface interactions, including indications of modifiability to assist service improvement endeavours.

We did not include nursing staff as participants as the focus was medication review and prescribing activities, although ACCP and Outreach Teams are primarily from professional nursing backgrounds, making it likely we captured the main themes here as well. One interview of a hospital junior doctor failed to record properly. The potential impact was minimised by the use of interviewer field notes (main points summary), and we benefitted from other participant interviews from the same profession. RSB is a critical care pharmacist and there is potential for bias and positionality to influence the interpretation of the results. We are confident that any risk of these was minimised by a non-ICU healthcare professional (MJ) leading the interviews, frequent reflexive evaluation, undertaking of dual coding of all interviews and using an iterative process to identify the themes and sub-themes based on submersion in the data and continuous matching of extracts to the thematic analysis.

### Recommendations for policy change and further research

Hospital-wide integration of e-prescribing systems across ICU and the hospital ward areas is imperative across this interface in patient care. These e-prescribing systems must have the required functionality (including automation when applicable), to support delivery of medication safety, being informed by an understanding of the information and actions required to effectively undertake component tasks.^26^ Patient flow systems across the ICU to ward transfer interface should be reviewed and optimised, to identify and implement modifiable elements to maximise multiprofessional team processes and care continuity within existing time constraints. Inter team communication processes around medication-information need to be streamlined, automated (when possible), co-ordinated and accessible, with structured modes of delivery. Task organisation should include delegation to specific staff groups with the knowledge and skills to routinely undertake medication reviews prior to patient transfer to a hospital ward (eg, critical care pharmacists and ACCPs). However, for clinical pharmacists to be able to take on routine delivery of these additional roles, their availability needs to be dependable and ensure sufficient staff are available 7 days per week. In terms of team organisation, all staff need to understand their responsibilities and those of colleagues in this facet of patient care to improve team performance. Further research is required to confirm the relationship between medication transfer errors and adverse drug events,^8^ with ICU patient outcomes. Such evidence would inform staff beliefs about consequences, underpinning the education of staff on the clinical importance and how systems can be used to minimise MEs in this interface of care. Furthermore, this education needs to translate into ICU junior medical staff competency assessments to further support improvements in system effectiveness. Further research is required to understand how ICU patients and/or their family should be more engaged and involved in medication-related information, enabling more participation in their own treatment and recovery.

## Conclusions

We identified crucial factors within five key themes that ICU and ward-healthcare professionals viewed were important in the safety and continuity of medication for ICU patients transitioning to a hospital ward. The complexity of the interactions on the performance and time dependency was clear. We make recommendations for policy change and further research based on improving: availability of hospital-wide integrated and functional electronic prescribing systems, patient flow systems, sufficient multiprofessional critical care staffing, knowledge and skills of staff, team performance, communication and collaboration, and patient and family engagement.

## Supplementary Material

Reviewer comments

Author's manuscript

## Data Availability

Data are available upon reasonable request.
