# Supplementary material for: Understanding medication safety involving patient transfer from intensive care to hospital ward: a qualitative sociotechnical factor study
Source: BMJ Open. 2023 May 2;13(5):e066757. doi: 10.1136/bmjopen-2022-066757 (PMC10163459; doi:10.1136/bmjopen-2022-066757)
Supplement: Supplementary data [file bmjopen-2022-066757supp001.pdf]

Supplementary File

The multicentre study was conducted in 4 general intensive care units (ICUs) across 3 Trusts in the north of England (Sheffield Teaching Hospitals (Sheffield Teaching Hospitals NHS Foundation Trust), Leeds Teaching Hospitals (Leeds Teaching Hospitals NHS Trust), The Salford Royal Hospital and Royal Oldham Hospital Hospital (both Northern Care Alliance NHS Foundation Trust)).

Centre	Hospital Type	ICU	Ward	Comment
Sheffield Teaching Hospitals NHS Foundation Trust	Teaching	e-prescribing (MetaVision, iMDsoft)	e-prescribing (Lorenzo, DXC Technology)	No inter-system electronic communication. All ward-based infusion prescriptions on paper
Salford Royal Hospital	Teaching	e-prescribing (MedChart)	e-prescribing (MedChart)	Inter-system communication. Complex and variable rate infusions are prescribed on paper
Royal Oldham Hospital	District General	e-prescribing (MedChart)	e-prescribing (MedChart)	Inter-system communication. Complex and variable rate infusions are prescribed on paper
Leeds Teaching Hospitals NHS Trust	Teaching	e-prescribing (Allscripts)	e-prescribing (Allscripts)	Inter-system communication. Total parenteral nutrition (TPN) prescribed on paper

Table S1. Electronic prescribing system configurations in ICU and hospital ward at each site

**Case 1 (slip and lapse type mistake)**
<u>Error:</u> Missed prescription for an antihypertensive drug (losartan) on ICU and hospital discharge, with that patient being hypertensive on return to GP practice.
<u>Contextual factors:</u> The losartan had not been prescribed in ICU as the patient had initially been hypotensive. On ICU discharge the patient was normotensive. The ICU was busy and only the medicines the patient was currently receiving whilst on ICU were transferred to the ward electronic prescription system. Losartan was on the ICU pre-admission

Bourne RS, *et al. BMJ Open* 2023; 13:e066757. doi: 10.1136/bmjopen-2022-066757

medication record, but this list was not transferred to the ward electronic system and the medicines reconciliation paper report was not reviewed on the ward.
**Case 2 (rules-based mistake)**
Error: ICU patient with Type 1 diabetes had their blood glucose controlled by a variable-rate insulin infusion. Their long-acting basal insulin was not prescribed whilst they were on a high-dose metaraminol infusion (as per ICU guidelines). The metaraminol was weaned off and the patient transitioned to the ward still without their basal SC insulin.
Contextual factors: Hospital guidelines for all Type 1 diabetic patients on a variable-rate insulin infusion is to continue their usual subcutaneous basal insulin to reduce the risk of diabetic ketoacidosis if the infusion is interrupted. ICU medical and pharmacy staff did not start the basal insulin after the metaraminol infusion was discontinued and this error was not identified prior to the ward transition. Only active medicines were transferred to the ward prescribing system. The Diabetes Specialist Team identified the error when they reviewed the patient on the ward.
**Case 3 (knowledge-based mistake)**
Error: An ICU patient receiving TPN was discharged to a ward without TPN prescribed on the paper fluid chart and didn't receive any TPN over the weekend (2 days).
Contextual factors: The trainee medical staff didn't know they needed to transfer the electronic TPN prescription from the ICU system to the paper fluid chart for the ward. The discharge was at the weekend and wasn't checked by a clinical pharmacist. Ward staff did not identify the patient was on TPN until the Nutrition Support Team reviewed the patient on Monday.

Table S2. Behavioural ICU transition medication error case vignettes

Interview Topic Guide

Interview questions:

Designation

1. What is your specific role at the hospital?
- How long have you been in this post?

Prior understanding of ICU patient transfer medication errors and local systems

2. Can you tell me about any prior awareness/knowledge/experience you have of any ICU patient transfer medication errors?
- If so, were these due to an error with medication review, or prescribing or both?
- In your opinion, do such errors significantly impact on patient care or not?
- Is that in the hospital or the community or both?
  (Explore if this is primarily an ICU, or hospital word, or GP issue or does it traverse all of these areas?)
  How are these errors usually detected?
3. Can you describe any procedures or safety systems are in place in your hospital to reduce medication errors on the interface of ICU patient transfers to the ward?
   (Is there an ICU discharge procedure? If so, please tell me about that discharge procedure? Do outreach services routinely review all patient's medicines on the ward?
4. How well are these procedures and safety systems implemented?

Who is responsible for the delivering the various safety systems mentioned?

Is there clear task allocation by profession or group?

How well are they adhered to in practice?

Case vignette discussions

- Case 1 (slip and lapse type mistake). Error and Contextual factors

So Case 1 is an error in which somebody forgets to do something they should or accidentally do something they shouldn't have done

5. Is this type of error common in your opinion?

How common? *[Rare to very common etc?]*

What do you think contributes most to slips and lapse type errors?

- Can signpost – London Protocol contributory influencing factors e.g. communication, workload, knowledge or skills if asked)

6. What things would help ro reduce errors like these?

   Then, if need be, prompt the participant to talk specifically about procedural/system/staff role contributions as required.

- Case 2 (rules-based mistake). Error and Contextual factors

So Case 2 is an error in which somebody incorrectly applies or doesn't apply a rule to a situation

What do you think contributes most to rule-based errors?

- Can signpost – London Protocol contributory influencing factors e.g. communication, workload, knowledge or skills if asked)

7. Is this type of error common in your opinion?
8. How common? *[Rare to very common etc?]*
9. What things would help to reduce errors like these?"

   Then, if need be, prompt the participant to talk specifically about procedural/system/staff role contributions as required.

- Case 3 (knowledge-based mistake). Error and Contextual factors

So case 3 is an error in which somebody misunderstands or misinterprets what is happening

What do you think contributes most to knowledge-based errors?

- Can signpost – London Protocol contributory influencing factors e.g. communication, workload, knowledge or skills if asked)

10. Is this type of error common in your opinion?
11. How common? *[Rare to very common etc?]*
12. What things would help ro reduce errors like these?"

Then, if need be, prompt the participant to talk specifically about procedural/system/staff role contributions as required.

Which error(s) do you most frequently encounter?

(Thinking about these errors in general; what is your role in mitigating the risk of these happening?

What could your role be?)

Facilitators and barriers to medication review (ICU patients on ward transfer)

Thinking about medication review for ICU patients during transfer to a ward:

How well does this process work?

What helps you to do it effectively?

What problems do you encounter?"

[*You can use the SEIPS framework to probe the participant for specific areas as required*]

Facilitators and barriers to safe prescribing and monitoring (ICU patients on ward transfer)

Thinking about prescribing and monitoring of medicines on the interface of ICU ward transfer:

How well does this process work?

What helps you to do it effectively?

What problems do you encounter?"

[You can use the SEIPS framework to probe the participant for specific areas as required]

Reducing medication review and/or medication errors on the interface of ICU patient transfer to a ward

13. If you could change one thing to improve the safety and continuity of medication on the ICU to ward interface – what would that be?
14. Have you any other comments or observations on the topic of medication continuity or safety in ICU patients on the interface of ward transfer?
15. Thank you for your time and helpful views.

Factor Types	Contributory influencing factor
Patient factors	Condition (complexity and seriousness)<br>Language and communication<br>Personality and social factors
Task and<br>technology factors	Task design and clarity of structure<br>Availability and use of protocols<br>Availability and accuracy of test results<br>Decision-making aids
Individual (staff) factors	Knowledge and skills<br>Competence<br>Physical and mental health
Team factors	Verbal communication<br>Written communication<br>Supervision and seeking help<br>Team structure (congruence, consistency, leadership etc.)
Work environmental factors	Staffing levels and skills mix<br>Workload and shift patterns factors<br>Design, availability and maintenance of equipment<br>Administrative and managerial support<br>Environment<br>Physical
Organisation & management factors	Financial resources and constraints<br>Organisational structure factors<br>Policy, standards and goals<br>Safety culture and priorities
Institutional context factors	Economic and regulatory context<br>National health service executive<br>Links with external organisations

London Protocol: Framework of contributory factors influencing clinical practice

**Work System**	Team members
	Tasks
	Technologies and tools
	Organisation
	Physical environment
	External environment
**Process ("Medication Review & prescribing")**	Information gathering
	Information integration & Interpretation
	Medication plan
	Communication of the plan
	Treatment (Medication received & monitoring)
**Outcomes**	Patient (Adverse events, increased length of hospital stay, readmission to ICU or hospital, death)
	System (Effects on quality, safety, cost, efficiency, morale, public confidence in the health system)
	Learning from medication errors

SEIPS 3.0 Framework

Bourne RS, *et al. BMJ Open* 2023; 13:e066757. doi: 10.1136/bmjopen-2022-066757
