# Supplementary material for: Understanding medication safety involving patient transfer from intensive care to hospital ward: a qualitative sociotechnical factor study
Source: BMJ Open. 2023 May 2;13(5):e066757. doi: 10.1136/bmjopen-2022-066757 (PMC10163459; doi:10.1136/bmjopen-2022-066757)
Supplement: Author's manuscript [file bmjopen-2022-066757.draft_revisions.pdf]

BMJ Open is committed to open peer review. As part of this commitment we make the peer review history of every article we publish publicly available.

When an article is published we post the peer reviewers' comments and the authors' responses online. We also post the versions of the paper that were used during peer review. These are the versions that the peer review comments apply to.

The versions of the paper that follow are the versions that were submitted during the peer review process. They are not the versions of record or the final published versions. They should not be cited or distributed as the published version of this manuscript.

BMJ Open is an open access journal and the full, final, typeset and author-corrected version of record of the manuscript is available on our site with no access controls, subscription charges or pay-per-view fees (http://bmjopen.bmj.com).

If you have any questions on BMJ Open's open peer review process please email info.bmjopen@bmj.com

BMJ Open

Understanding medication safety involving patient transfer from intensive care to hospital ward: A qualitative socio-technical factors study

Journal:	*BMJ Open*
Manuscript ID	bmjopen-2022-066757
Article Type:	Original research
Date Submitted by the Author:	19-Jul-2022

Complete List of Authors:	Bourne, Richard; Sheffield Teaching Hospitals NHS Foundation Trust, Departments of Pharmacy and Critical Care; The University of Manchester, Division of Pharmacy and Optometry, School of Health Sciences<br>Jeffries, Mark; University of Manchester, Division of Pharmacy and Optometry, School of Health Sciences; NIHR Greater Manchester Patient Safety Translational Research Centre<br>Phipps, Denham; The University of Manchester, Division of Pharmacy and Optometry, School of Health Sciences<br>Jennings, Jennifer K; Sheffield Teaching Hospitals NHS Foundation Trust, Departments of Pharmacy and Critical Care<br>Boxall, Emma; Northern Care Alliance NHS Foundation Trust, Salford Royal Hospital, Pharmacy Department<br>Wilson, Franki; Leeds Teaching Hospitals NHS Trust, Pharmacy Department<br>March, Helen; Northern Care Alliance NHS Foundation Trust, Royal Oldham Hospital, Pharmacy Department<br>Ashcroft, Darren; The University of Manchester, Division of Pharmacy and Optometry, School of Health Sciences; NIHR Greater Manchester Patient Safety Translational Research Centre

Keywords:	Adult intensive & critical care < INTENSIVE & CRITICAL CARE, Health & safety < HEALTH SERVICES ADMINISTRATION & MANAGEMENT, Quality in health care < HEALTH SERVICES ADMINISTRATION & MANAGEMENT, QUALITATIVE RESEARCH

SCHOLARONE™
Manuscripts

I, the Submitting Author has the right to grant and does grant on behalf of all authors of the Work (as defined in the below author licence), an exclusive licence and/or a non-exclusive licence for contributions from authors who are: i) UK Crown employees; ii) where BMJ has agreed a CC-BY licence shall apply, and/or iii) in accordance with the terms applicable for US Federal Government officers or employees acting as part of their official duties; on a worldwide, perpetual, irrevocable, royalty-free basis to BMJ Publishing Group Ltd ("BMJ") its licensees and where the relevant Journal is co-owned by BMJ to the co-owners of the Journal, to publish the Work in this journal and any other BMJ products and to exploit all rights, as set out in our licence.

The Submitting Author accepts and understands that any supply made under these terms is made by BMJ to the Submitting Author unless you are acting as an employee on behalf of your employer or a postgraduate student of an affiliated institution which is paying any applicable article publishing charge ("APC") for Open Access articles. Where the Submitting Author wishes to make the Work available on an Open Access basis (and intends to pay the relevant APC), the terms of reuse of such Open Access shall be governed by a Creative Commons licence – details of these licences and which Creative Commons licence will apply to this Work are set out in our licence referred to above.

Other than as permitted in any relevant BMJ Author's Self Archiving Policies, I confirm this Work has not been accepted for publication elsewhere, is not being considered for publication elsewhere and does not duplicate material already published. I confirm all authors consent to publication of this Work and authorise the granting of this licence.

For peer review only

Understanding medication safety involving patient transfer from intensive care to hospital ward: A qualitative socio-technical factors study

Richard S Bourne[1,2*], Mark Jeffries[2,3], Denham L Phipps[2], Jennifer K Jennings[1], Emma Boxall[4], Franki Wilson[5], Helen March[6], Darren M Ashcroft[2,3]

[1] Departments of Pharmacy and Critical Care, Sheffield Teaching Hospitals NHS Foundation Trust, Sheffield, UK

[2] Division of Pharmacy and Optometry, School of Health Sciences, Faculty of Biology, Medicine and Health, The University of Manchester, Manchester, UK

[3] National Institute for Health Research (NIHR) Greater Manchester Patient Safety Translational Research Centre (PSTRC), School of Health Sciences, Faculty of Biology, Medicine and Health, The University of Manchester, Manchester, UK

[4] Pharmacy Department, Salford Royal Hospital, Northern Care Alliance NHS Group, Salford, UK

[5] Pharmacy Department, Leeds Teaching Hospitals NHS Foundation Trust, Leeds, UK

[6] Pharmacy Department, Royal Oldham Hospital, Northern Care Alliance NHS Group, Oldham, UK

*Corresponding author

E-mail: richard.bourne1@nhs.net

Word count : 7146

Keywords: Critical Care, Medication Errors, Patient Safety, Systems Analysis, Transitions

For peer review only

For peer review only - http://bmjopen.bmj.com/site/about/guidelines.xhtml

Abstract

Objective

To understand the socio-technical factors affecting medication safety when intensive care patients are transferred to a hospital ward. Consideration of these medication safety factors would provide a theoretical basis, on which future interventions can be developed and evaluated to improve patient recovery.

Design

Qualitative study using semi-structured interviews of intensive care and hospital ward-based healthcare professionals. Transcripts were anonymised prior to thematic analysis using the London Protocol and Systems Engineering in Patient Safety 3.0 model frameworks.

Setting

Four north of England National Health Service hospitals. All hospitals used electronic prescribing in intensive care and hospital ward settings.

Participants

Intensive care and hospital ward healthcare professionals (intensive care medical staff, advanced practitioners, pharmacists and outreach team members; ward-based medical staff and clinical pharmacists).

Results

Twenty-two healthcare professionals were interviewed. We identified thirteen factors within five broad themes, describing the interactions that most strongly influenced the performance of the intensive care to hospital ward system interface. Important factors at an intensive care and wider organisational level are highlighted. The themes were: Complexity of process performance and interactions; Time pressures and considerations; Communication processes and challenges;

For peer review only

For peer review only - http://bmjopen.bmj.com/site/about/guidelines.xhtml

Technology and systems; and Beliefs about consequences for the patient and organisation. Time pressure considerations have a marked negative impact on the system performance.

Conclusions

The complexity of the interactions on the system performance and time dependency was clear. We make several recommendations for policy change and further research based on improving: knowledge and skills of staff, sufficient critical care staffing, team performance, communication and collaboration, and patient and family engagement.

Strengths and limitations of this study

- Medication safety for intensive care patients transitioning to a hospital ward requires co-ordination of care within and across multiprofessional teams and environments with varying staff and technological resources.
- This is the first qualitative research study focused on understanding the socio-technical factors of medication safety in patients transitioning from intensive care to a hospital ward.
- Study strengths include multicentre views of intensive care and hospital ward UK healthcare professionals, representing teaching and district general hospitals, all using electronic prescribing systems
- Thematic analysis using the Systems Engineering Initiative for Patient Safety (SEIPS 3.0) model enabled evaluation the socio-technical factors of medication safety during transition from intensive care to a hospital ward.
- A potential limitation of our focus on medication review and prescribing activities was not including nursing staff as participants, although it is likely we captured the main themes here as well with inclusion of other staff from professional nursing backgrounds.

For peer review only - http://bmjopen.bmj.com/site/about/guidelines.xhtml

Background

Patients surviving an episode of critical illness requiring intensive care often experience a protracted recovery, requiring ongoing healthcare resources. For most patients, the first step in this recovery is transfer to hospital ward-level care. This is a notable transition in care, where patients can be exposed to increased safety and care continuity risks. Medication safety in this care transition requires co-ordination of care within and across multiprofessional teams and environments with varying staff and technological resources. This study aimed to understand the socio-technical factors affecting medication safety when ICU patients are transferred to a hospital ward. Consideration of these medication safety factors would provide a theoretical basis, on which future interventions can be developed and evaluated to improve patient recovery.

Methods

Multicentre qualitative study using semi-structured interviews of intensive care and hospital ward-based healthcare professionals from four centres in the north of England. All centres used electronic prescribing in intensive care and hospital ward settings. Transcripts were anonymised prior to thematic analysis using the London Protocol and Systems Engineering in Patient Safety 3.0 model frameworks. All coding was undertaken in duplicate and completed in three rounds.

Results

Twenty-two healthcare professionals were interviewed (intensive care medical staff, advanced practitioners, pharmacists and outreach team members; ward-based medical staff and clinical pharmacists). We identified thirteen factors within five broad themes, describing the interactions that most strongly influenced the performance of the intensive care to hospital ward system interface. Important factors at an intensive care and wider organisational level are highlighted. The themes were: Complexity of process performance and interactions; Time pressures and considerations; Communication processes and challenges; Technology and systems; and Beliefs

For peer review only

about consequences for the patient and organisation. Time pressure considerations have a marked negative impact on the system performance.

Conclusions

The complexity of the interactions on the system performance and time dependency was clear. We make several recommendations for policy change and further research based on improving: knowledge and skills of staff, sufficient critical care staffing, team performance, communication and collaboration, and patient and family engagement.

For peer review only

For peer review only - http://bmjopen.bmj.com/site/about/guidelines.xhtml

Background

Patients recovering from a critical illness requiring intensive care unit (ICU) treatment are generally transferred to a high-dependency area or hospital ward, before eventual hospital discharge to the community. However, this simplified summary of transition steps for ICU survivors fails to capture the patient pathway complexity, where full patient recovery may be protracted, traversing hospital and community care and involve several care transitions [1,2]. Compared to patients discharged from standard hospital care, ICU patients have worse short and medium-term outcomes, requiring additional ongoing healthcare resources [3]. After hospital discharge, the need for emergency hospital readmission is common for ICU patients, with half of patients readmitted within a year [4]. Pre-existing patient multimorbidity, frailty and polypharmacy contribute to this hospital re-admission risk [4,5]. There is a growing appreciation of the need for effective patient rehabilitation to enhance post-ICU recovery, the course of which can be complicated by polypharmacy and care fragmentation [1,6]. Indeed, medication changes are extensive during a patient's ICU episode [7,8], increasing the risks for hazardous polypharmacy [9]. Medication errors (MEs) occurring during an ICU stay may remain uncorrected beyond the acute hospital care episode [10–13], with pre-existing polypharmacy burden a risk predictor [14].

Transitions in care are known to increase patient risks, including those related to medicines usage and care continuity [15]. The transition from ICU to a hospital ward is a marked interface in patient care, with contrasting resources in care team and technological support available in the respective clinical areas. Transition from ICU exposes patients to high rates of MEs [16–18], and adverse events are common, including those that are medication-related [19]. ICU care may therefore compound the known complexities and challenges for patient medication care created by care transitions [15,20].

Whilst transition in patient care from ICU to the hospital ward is recognised as a high-risk step in the ICU patient care progress, it remains unclear how medication safety and continuity should be

For peer review only - http://bmjopen.bmj.com/site/about/guidelines.xhtml

assured [21], and how these impact on the trajectory of patient recovery. Structured staff handovers between ICU and the hospital ward are recommended to support the continuity of care including a focus on medication [22]. Whilst broader indicators of interventions that can improve medication safety on transitions in patient care are provided by the World Health Organization (WHO) and ICU expert views [15,23]. To date, interventions to improve the safety and continuity of medication for ICU patients transitioning to a hospital ward have focused on education of staff, medication review, guidelines, electronic transfer/ handover tool or letter and medicines reconciliation [24]. However, for any intervention or package of interventions to be effective in the ICU environment and improve patient care, it is important to understand how the ICU system works [25]. Furthermore, the care interfaces need to be examined in terms of their systems, processes and outcomes [26].

Care of the critically ill patients in ICUs requires effective and efficient co-ordination of diverse multiprofessional teams, working in a dynamic and demanding clinical environment. Safe and effective use of medicines in these vulnerable ICU patients requires frequent reassessment of treatment goals, medication review, monitoring and dose titration involving multiple ICU team members, supported by tools and technologies such as clinical information and electronic prescribing systems. Furthermore, error prevention in ICU care is dependent on effective inter and intra-multiprofessional team working and communication [27], that are particularly important on patient transition from ICU to the hospital ward [28,29]. Despite ICUs being technology rich environments, an over-reliance on technology to mitigate against medication errors underestimates the complexity of the systems and processes [30,31]. Like other parts of healthcare, ICU and its care transitions, involve a complex interplay between technology, tasks and people [32]. Therefore the importance of human factors engineering have been promoted to address the whole ICU medication management and patient safety pathway [27,33,34].

This study aimed to understand the socio-technical factors affecting medication safety when ICU patients are transferred to a hospital ward. Consideration of these medication safety factors would

For peer review only

For peer review only - http://bmjopen.bmj.com/site/about/guidelines.xhtml

provide a theoretical basis, on which future interventions can be developed and evaluated to improve patient recovery.

For peer review only

For peer review only - http://bmjopen.bmj.com/site/about/guidelines.xhtml

Methods

Ethical (The University of Manchester, Ref: 2020-10852-17342) and NHS Health Research Authority (HRA) (IRAS 292456) approval was granted for the study. Our study used a qualitative design, intended to solicit adult, general ICU and ward-based healthcare professionals directly involved in the prescribing and medication review of ICU patients on the interface of transition to the hospital ward. We recruited from a purposive sample of healthcare professionals across four NHS centres, chosen to represent both District General and Teaching Hospitals in the north of England with a representative range and combination of ICU and hospital ward e-prescribing systems (Supplementary File, Table S1). The sampling frame was ICU (medical staff, advanced critical care practitioners (ACCPs), critical care pharmacists and ICU outreach team members) and ward-based (medical staff, clinical pharmacists) healthcare professionals. Principal Investigators at each NHS hospital, directed participant email invitations via departmental team leads or departmental email distribution lists. All participants provided informed written consent. We recruited 22 participants representing each professional group and clinical location (see Table 1 for more details) [35].

Healthcare professional staff group	ICU Medical Staff	Advanced Critical Care Practitioners (ACCP)	Critical Care Pharmacists	Outreach Team Members	Ward Medical Staff	Ward Clinical Pharmacists
**n**	3*	4	4	4	3*	4
**Time in current role (years, mean (SD))**	2.7 (2.1)	6 (2.2)	11 (12.8)	12.3 (5.5)	2 (1)	1.5 (1.3)

Table 1: Healthcare professional participants (n=22)

* Participants from sites 1,3 and 4 only

We undertook semi-structured interviews facilitated by a topic guide adapted from the London Protocol [36] and Systems Engineering Initiative for Patient Safety (SEIPS) 3.0 model [26] (Supplementary File, Topic guide). The London Protocol was developed from Reason’s "Swiss Cheese" model, capturing the contributing factors and context around a specific patient safety incident [36]. The SEIPS models are based on the System-Process-Outcome description of

For peer review only

For peer review only - http://bmjopen.bmj.com/site/about/guidelines.xhtml

approaches to measuring and improving the quality of health care. Version 3.0 of the SEIPS framework includes an expanded process component, focusing on the patient pathway to describe patient interactions with multiple care settings over time [26]. In order to prompt discussion, we selected three reported ICU medication error cases as vignettes, each representing a type of human error from Reason's (1990) generic error modelling system [37] (see Supplementary File, Table S2). The vignettes were shared with participants in advance of the scheduled interview. All interviews were undertaken via video telecommunication software (Microsoft Teams, Microsoft Corporation, USA), recorded and transcribed verbatim. A qualitative researcher (BBB) experienced in investigation of medicines optimisation practices in NHS healthcare environments conducted the interviews. Transcripts were anonymised prior to thematic analysis using the London Protocol [36], and SEIPS 3.0 model [26] frameworks. Coding was supported through qualitative research software (NVivo, Version 12, QSR International (UK) Limited) and undertaken in duplicate by the qualitative researcher (BBB) and Chief Investigator (AAA). The coding was completed in three rounds with discussions between the coders after each round, and with the wider research team at different intervals. We understood data-saturation as being achieved when we judged further interpretation of codes and themes would not provide further insights [35]. Finally, through multiple discussions amongst the research team, we coalesced the identified factors until they provided a table and parsimonious account of the interactions that most strongly influence the performance of the ICU to hospital ward work system and communication pathways.

Patient and Public Involvement

We have actively engaged patient and public involvement (PPI) is this wider research intervention development programme to improve medication safety for intensive care patients transitioning to a hospital ward. In this study, PPI colleagues consulted on and contributed to the development of the topic guide, interpretation of the thematic findings and dissemination of results.

For peer review only - http://bmjopen.bmj.com/site/about/guidelines.xhtml

For peer review only

For peer review only - http://bmjopen.bmj.com/site/about/guidelines.xhtml

Results

We identified thirteen factors within five broad themes, providing a detailed description of the ICU-hospital work-system interface. This identified the complexity and interactions within the system and accounted for the significant impact of time pressures and considerations (Table 2, Fig 1). The communication pathways utilised are presented (Fig 2).

ICU-Hospital Ward Work System Interface
ICU Active Agents: ICU Consultant, ICU Junior medical staff, Advanced Critical Care Practitioner, Critical Care Pharmacist, ICU Nurse, Outreach Team, Microbiologist<br>*Hospital Ward Active Agents*: Junior medical staff, Specialist Teams (e.g. Diabetes team, Acute Pain Team), Clinical Pharmacist, Nurse, Outreach Team
**Performance is shaped most *strongly* by interactions of:**
Care Team Factor(s)<br>-CT1: Knowledge and skills of staff on medication review and safe prescribing for ICU patients on the interface of transition to the hospital ward*<br>-CT2: Beliefs about consequences, understanding of staff to the frequency and clinical impact of medication errors on patient recovery*
Task Factor(s)<br>-TA1: Task and patient complexity affected by the number of long-term and acute medicines used in patients with a critical illness with unpredictable recovery and responses to treatment including medication<br>-TA2: Task prioritisation, often focused on the new patient admission(s) or more unstable patient(s) in ICU, with medication use in ICU transfer patient deemed low priority*<br>-TA3: Task allocation and delegation amongst the care team*
Tools and Technologies Factor(s)<br>-TT1: E-Prescribing system integration and functionality**<br>-TT2: Electronic patient notes accessibility and searchability**<br>-TT3: Medication transfer checklist completion and adherence*
Organisational Factor(s)<br>-O1: Team performance (organisation, leadership, collaboration and dependability)*<br>-O2: Multiprofessional ward rounds including a medication review element*<br>-O3: Communication between ICU and Hospital Ward team members*/**<br>-O4: Time pressures in the need to complete the ICU patient transfer to the ward, staff workload and out of hours transfers*/**<br>-O5: Safety culture in ICU informed by incident reviews, audit and feedback and follow up clinic experiences*
Physical Environment Factor(s)<br>-PE1: ICU bed availability and pressure for beds**
External Environment Factor(s)<br>-EE1: Bed availability on the hospital ward and transfer times**<br>-EE2: Transfer time standards
On ICU, Patient and Family co-agents*, also Dietitian, Nutrition support team, Visiting teams and Pharmacy Technician. On the Hospital Ward, the Rehabilitation Team were co-agents.

Table 2: ICU-Hospital Ward Work System Interface

*Potentially modifiable at a local ICU level; ** potentially modifiable at an organisational level

For peer review only - http://bmjopen.bmj.com/site/about/guidelines.xhtml

Fig 1: ICU patient transfer performance interaction

Fig 2: Medication continuity and safety communication across ICU to hospital ward interfaces

For peer review only

Theme 1. Complexity of process performance and interactions

Care Teams and Collaboration

A professionally diverse range of ICU and hospital ward-based healthcare staff undertook important tasks and processes to support medication safety for ICU patients transferring to a hospital ward (Table 2). In the ICU, participants emphasised the value of collaborative, multiprofessional team working that was driven by strong team leadership. ICU consultants led multiprofessional ward rounds, provided leadership and examples for more junior staff in the routine delivery of patient medication reviews. ACCP, medical and nursing staff had important roles in medication review. The role of the critical care pharmacist in medicines reconciliation on admission and undertaking medication reviews for patients, including during ward rounds and prior to hospital discharge was highlighted.

the MDT ward round is really useful, as the [..] consultant you're trying to pull a broad picture of everything together for discharge, and medications is one part of that, and I think we're very lucky in (name of hospital), the quality and the provision of our pharmacy support is excellent. [..] I don't see how we could do it without the pharmacists. **(P5 ICU Consultant)**

On the hospital ward, there was also evidence of multiprofessional team working in undertaking medication reviews in ICU patients transferred to the hospital ward. In some cases, these were facilitated by consultant-led ward rounds. Whilst direct collaboration between the ICU and hospital ward team appeared less clear, the ICU outreach team did provide an important link between the two clinical areas.

So I follow patients up post-discharge from intensive care [...] to look at them physiologically, psychologically, and just follow the plan that's been put in place on a critical care area. [...] Regarding medication, if there's something specific in the discharge plan, then I would review that aspect. **(P1 Outreach Team)**

On the hospital ward, referral to clinical pharmacists and specialist teams in undertaking medication reviews were highlighted. The diabetes team were seen as essential for patients receiving insulin therapy when transferred to a hospital ward and the acute pain team were often engaged with patients with ongoing pain issues.

But there's also other specialists that we get involved [...] we should automatically make a diabetic team referral. So, it's also pulling in other specialist teams into the mix [...] the diabetic team can give the ward team a plan of action ... **(P3 ACCP)**

For peer review only - http://bmjopen.bmj.com/site/about/guidelines.xhtml

Tasks, processes and prioritisation

The key medication-related tasks undertaken by ICU healthcare professionals interviewed were medication review, prescribing and plan setting. These were supported by processes such as medicines reconciliation and, in some units, a transfer checklist that included medication elements. ICU transfer reports sometimes included medication information such as pre-admission medicines, current medication and medication plans. Transcribing and transcription checks were also undertaken in ICUs without integrated ICU and hospital ward electronic prescribing systems, which also provided a medication review opportunity.

I would say [the transcription process is] vitally important. So, part of the problem is […] it shouldn't really be seen as a transcription process, it should be more of a review process. […] it's not simply passing straight from one system to another including all that information, it's actually reviewing what's appropriate. **(P2 Critical Care Pharmacist)**

The complexity of tasks undertaken was qualified within the context of ICU patient care and regarded as impacting on medication safety throughout the patient journey. The acuity of the patient condition, and increasing number of medicines they received, contributed to medication review complexity.

we're now seeing patients who are more complex than ever before and on more medicines that even before, and then, obviously, that becomes even more complex in itself. **(P9 ICU Consultant)**

Tasks were often prioritised, which sometimes meant that the medication-related tasks were seen as a low or lower priority and '*probably the least important thing you would do that day.'* **(P13 Ward Junior Doctor)**. The seriousness of the patient's condition determined the time spent on their care; more seriously ill patients were prioritised over the patient transferring from ICU.

And because it's quite a fast-paced unit those patients could be gone before I see them again because then I could be reviewing the critical unwell patients, the Level 3 patients, the ones that are intubated, and things. So, those patients that are then getting discharged are in the back of my mind because I'm dealing with the sicker ones. **(P14 ACCP)**

The priority for ward-based clinical pharmacists was often focused on undertaking medicines reconciliation for new hospital admissions and discharge prescriptions, over reviewing patients transferred from ICU. Whilst ward medical staff focused on critical medicines.

So I think when you're on the ward round and you go to review the medicines, you mainly look at things that are critical to the patient at the time first. So antibiotics, blood thinners and that sort of thing. And

For peer review only - http://bmjopen.bmj.com/site/about/guidelines.xhtml

looking back to admission meds to see what's missing while they're still in hospital is not always top of your list. **(P20 Ward Junior Doctor)**

Task allocation and delegation

Task allocation and task delegation were important considerations. Participants had differed in how they rated the importance of a medication-related tasks. Amongst medical staff, transfer planning around medication was often allocated to a junior team member and this was seen as a "low-level" task. Whereas critical care pharmacists and ACCPs considered these more complex tasks.

A lot of them [junior doctors] will just write, see [electronic prescription], and that's it. I notice that more and more recently. They aren't actually doing a formal review of the meds. […] it's their first job in critical care, and they can just get lumbered to do these discharge summaries because they are a straightforward thing to do […] They maybe don't go into the complexities that they should do in some of them. **(P14 ACCP)**

Moreover, when it came to medication review on ICU patient transfer, staff groups didn't always seem aware of the roles and responsibilities of other staff groups.

In terms of when they step down to the ward, I don't know what role our ICU pharmacists have there. I'm not sure I've actually known they've had any active role in that. I think they would flag if they're still on a medication that can't be given or they think their role is much more with new patients and new prescriptions in ICU. I'm sure ward pharmacists, when they go to the ward, I'm sure then they…I'm much more imagining it's their role to review the new prescriptions coming to the ward. **(P15 ICU Junior Doctor)**

ICU participants suggested responsibility for medication review lay with the hospital ward team or general practitioner and that '*someone else will do it.'* **(P9 ICU Consultant)**

It was suggested that a specific staff group or groups needed to take ownership of the medication review prior to the patients ICU to ward transfer. The ACCP or critical care pharmacist were considered the most appropriate staff groups to do this. Both these staff groups had the benefit of service stability over the frequent rotation of junior medical staff.

Knowledge, Skills and professional diversity

Different staff groups were felt to have different knowledge and skills, for example medical staff diagnostic and treatment planning skills versus pharmacist medication planning and prescribing skills.

For peer review only - http://bmjopen.bmj.com/site/about/guidelines.xhtml

I've got to say that the pharmacists and doctors, I would say the pharmacists within critical care are really good at identifying drugs that's been withheld and putting it in their plan. And then the doctors have got good at looking at the pharmacy plan and pulling it through into the final discharge report. **(P1 Outreach Team)**

ACCPs were thought to have knowledge and skills in medication reviews through having *'done the process hundreds of times'* **(P5 ICU Consultant)**, in comparison to consultants and junior doctors.

On the hospital ward, there appeared a hesitancy to review medication that had been commenced whilst the patient was in ICU. This seemed to stem from a lack of familiarity with ICU medication and a feeling that the ICU team knew what they were doing. This was compounded by lack of knowledge and skills of junior medical and pharmacy staff meant that acute medications could continue longer term.

I think it's… to get an effective medication review, I think it depends on the pharmacist and their experience, which has been the key thing. So I think if you've got really junior staff doing a medication review on a patient that's stepped down from ICU, I would imagine they'd find it difficult, […]. And I feel like they would be more reluctant to make medication changes or recommendations to doctors. **(P19 Ward Clinical Pharmacist)**

Theme 2. Time pressures and considerations

Time pressures

Time pressures and considerations affected the system performance (Fig 1). Time pressures contributed to staff not always completing information such as the rationale for prescribing, pausing or de-prescribing medication.

…a doctor changes a medicine or stops a medicine, then when you do it on the [e-prescribing system], you're supposed to fill in a box to say what you've done […] (people are) basically doing this as quite a quick exercise, they'll just go, click, click, click and then they bypass that very often. And we all do it … **(P11 Critical Care Pharmacist)**

Time pressures around the ICU transfer process contributed to some patients being transferred without the opportunity to undertake a prior medication review. Time pressures could mean that staff handovers did not occur and transfer reports and medication plans were not completed.

I think there is often a lot of time pressure on the discharge planning process and that would definitely make a slip type error, because you're so focused on getting the task done, you do what is necessary and then this is probably not absolutely necessary to get the patient discharged and complete your task

For peer review only

it's an additional thing isn't it? What you're trying to do is get the patient out onto the ward with everything they need, rather than what else? **(P5 ICU Consultant)**

Workload & staffing levels

One barrier to completing medication reviews was in the healthcare staff-patient ratio particularly on the hospital ward.

It's predominantly the number of patients to see. And also slightly lack of staff on the wards as well. Because if you just have one consultant and one junior, they'll be trying to look things up on the computer, get the meds up, talk to the consultant, see the patient, and then not always have time to then sit and do the prescription at the same time. If there is two, one can be looking stuff up and the other can be doing the prescribing and requesting at the same time. **(P20 Ward Junior Doctor)**

In particular the benefits of clinical pharmacist medication review were acknowledged to be hampered by reduced dependability borne out of insufficient staffing, lack of 7-day services and set daytime working patterns.

I think the biggest constraint in the system is provision of clinical pharmacy staff. […] actually having the pharmacists time available to undertake those medicines safety checks is the core part of business. And there's only a few of them, […] there's certain things that we as clinicians can do to mitigate that to a degree. […] well the root cause of the problem is […] insufficient pharmacy staff time. **(P9 ICU Consultant)**

Out of hours discharges

Out of hours transfer of patients from ICU to a hospital ward were regarded as particularly high-risk for medication errors.

…. particularly in out of hours, unfortunately, just because of bed availabilities and pressures, that can be quite challenging in terms of information being passed from one team to the next. So information is lost almost about new medications that are commenced for various reasons on the ICU. **(P21 ACCP)**

Discharges out of hours were seen as more problematic because of reduced ICU and hospital ward staffing levels, particularly with the unavailability of wider specialist teams and fewer senior healthcare staff available. It was felt that daytime discharges were conducted better, allowed '*the pharmacists (to) provide a review of the discharge*' **(P5 ICU Consultant)** and increased the likelihood of multiprofessional and senior medical staff review. Sometimes out of hours transfers were unavoidable when ICUs were full and acute admission to the unit were required for patient flow.

For peer review only

There are a lot of our patients that are discharged out of hours, after 6:00 pm, and that shouldn't be happening. Our patients should be out in a timely manner, sat on a ward, safety, so people have time to make sure everything's documented and sorted for that patient before the nightshift [...]. **(P14 ACCP)**

Bed availability and service standards

Patient flow challenges created pressures on ICU and hospital bed availability, which in turn created issues for patient transfer planning and review. The pressure to get a patient discharged within specified service standards was identified. Transfers could be rushed with short notice of ward bed availability.

...it could be that a patient's on ICU, they've been ready to go to the ward for four or five days, there's not been a bed available for them. And then we've got say three poorly patients out on the wards in A&E that urgently need a bed. So it's sort of a rapid discharge then to clear the beds. **(P7 Outreach Team)**

Theme 3. Communication processes and challenges

Effective communication was seen as a key safety component in transition of patient care '*working together with good communication'* **(P1 Outreach Team)**. Conveyance of accurate and complete medication information (e.g. medication changes) on patient transfer was felt important overall. Several challenges to effective communication were apparent. Firstly, the relatively large number of healthcare staff involved in information transfer was seen as a barrier to effective communication. Pertinent information could get lost in the sheer volume of exhaustive patient notes after an ICU care episode, creating an accessibility barrier. Barriers to information accessibility, and in the case of clinical pharmacists meant they reviewed fewer patients. Finally, information was not always communicated and staff would sometimes forget to include key details. Several systems were employed to improve communication of information including medication, principally handovers, transfer summaries and prescription annotations.

Handovers (ICU to hospital ward)

Staff utilised handover processes to convey information on transitions of patient care. Handovers were verbal, written or combinations thereof (Fig 2). ICU medical, nursing and pharmacy staff had handover processes to their respective hospital ward colleagues. Face-to-face handovers were seen by some as being the best method, whilst transfer of verbal information around shift changes or out of hours were considered higher risk for being lost in the system. Medical, nursing and pharmacy staff had limited awareness of what each other handed over and how this was undertaken. This

For peer review only - http://bmjopen.bmj.com/site/about/guidelines.xhtml

For peer review only

contributed to assumptions or uncertainties that other staff groups were handing this information over. There was some uncertainty over what information to convey about medication on transitions of care. Medical staff tended to only handover information that they perceived to be important, that needed to be followed up related to the key patient problems e.g., a scan needs to be requested. It was often unclear if medication details would be included or not, however, this information was often considered of low interest for medical staff.

I don't think the doctors, […] have a verbal handover. […] everything's documented on this transfer medication report and so it's at their discretion for them to read it and to make sure that everything's handed over. [..] But in terms of, like, a verbal handover I know that doesn't happen. ***(P18 Ward Clinical Pharmacist)***

Transfer summary

The transfer summary was considered the communication focus, providing an effective means of highlighting and collating important information and care plans, and considered more dependable than verbal handovers. When completed well, the transfer summary was used to convey information and prompt medication reviews.

So within that there are fields specifying medication comments and actions, ongoing actions for the ward. So we try to put into that the information, so it doesn't get lost and that's the way to try and improve that safety and that communication around medications. But if they become well to step out and then there's bed pressures and they don't step for a day or two that information can almost get lost within all the notes. **(P21 ACCP)**

Furthermore, having the transfer summary easily accessible electronically by all healthcare professional was deemed important since *'everyone knows it's there [..] the receiving team can read that'.* **(P7 Outreach Team)**

Prescription annotations

Annotations on the electronic prescription was seen as an effective way of maintaining focused, easily accessible information regarding medication. Prescribing important chronic medication and then holding or suspending it on the electronic prescribing system was seen as a prompt for medication review. However, annotated information was often omitted, particularly if transcription was required.

So, I think when it's done properly and it's suspended and… Our [e-prescribing system] is great, you can write all sorts of qualifiers, indicators, you can write all sorts on there as a note for future teams. And I

For peer review only

think that's when I think it makes most sense. And if I'm receiving a patient and someone's written a note to me, it's just pretty obvious what I've got to do. That all works well. I think when people just leave them off or stop them, but don't write reasons. When you stop any medication there is a comment to cease it and often that's not filled in either. **(P15 ICU Junior Doctor)**

Theme 4. Technology and systems

Technology was utilised to prescribe medication, record and store information about patients' medicines, prioritise workload and to facilitate communication with other healthcare professionals. Overall, electronic prescribing was felt to have safety benefits over paper prescription and charts with prescription defaults, order sets, improved decision support and prompts and accessibility to information such as medicines reconciliation. Electronic prescribing also made information retrieval about medication changes easier to undertake, aiding medication review.

Technology also supported the use of medication transfer checklists and had further potential application via automation of some processes such as information pooling and patient prioritisation.

There's also an inbuilt checklist into the critical care system, so when a patient's being discharged, the doctor is supposed to complete all the discharge information. And before they can do that there is a checklist which they are supposed to tick off which says, I have reviewed all the patient's usual medicines, I have reviewed all the current medicines to make sure that nothing's inappropriately being continued,... **(P2 Critical Care Pharmacist)**

However, it was noted that over time checklists became less effective as staff fatigue with the system and find workarounds. Although all ICUs used electronic prescribing, there was some requirement on specific ICUs to use paper prescriptions for some medicines (e.g. intravenous medicines and fluids). One centre had separate ICU and ward-based systems that did not communicate directly, creating an electronic transcription phase to ICU patient transitions. Participants were very clear on the importance of full integration of e-prescribing systems, so that all medication were fully visible and auditable in a single format and transcription was not required.

It would say be better if everything was electronic and go to the ward electronic, because things get misfiled, don't they? […] Lost. If it was all electronic and got sent electronically, then that would be the safest way. I think often with prescribing problems that can be a problem, when some things are electronic and some things are paper, then it gets very confusing, doesn't it? **(P12 ACCP)**

System functionality was emphasised in minimising the number of phases or menus required in the e-prescribing system to prescribe, hold, and plan reviews of medication. Limitations in functionality

For peer review only - http://bmjopen.bmj.com/site/about/guidelines.xhtml

disinclined staff to prescribe medication not immediately required, which compromised the identified communication benefits of prescribing and holding a potentially important long-term medication requiring regular review for restarting when clinically appropriate. The importance of accessibility was identified when it came to finding required information quickly, minimising loss of information within menu caches.

Over-reliance on the individual to undertake tasks increased the risk of them not being done.

I think, I think a lot of the errors occur because it's down to individuals to do things. And you can put systems in place, and you can put protocols in place, but people will always find a way around them, when you're busy you'll always take the easiest route.[…] Whereas, if you had ways of connecting up systems so that it's automatically done, then it's less reliant on the individuals. The individuals would still need to be involved in it and can still up on errors and add to the system, but you would probably get fewer errors, certainly less, sort of, information loss if the systems talked to each other. **(P2 Critical Care Pharmacist)**

Theme 5. Beliefs about consequences for the patient and organisation

Medication errors and adverse patient outcomes

Medication errors reported included inappropriate continuation of medication started in ICU without an ongoing indication, loss of important medication information (e.g. review plan), and failure to restart potentially important chronic medication. Although participants acknowledged such medication errors were very common, the outcomes for patients and the impact upon them was not something that participants seemed to universally appreciate or take into consideration.

I've never really thought about that [potential impact on patients]. *I would hope not. I would hope by the point we get to discharge the wards have reviewed all the medications properly and done a decent* [discharge prescription] *[…]. So, they're generally on medication short term to try and help psychosis, which I would hope gets stopped before they get discharged because it's not something we ask to be on long term, but I don't know if I'm honest. It's not something that we follow up on.* (**P14 ACCP**)

Participants identified that the implications would vary depending on certain factors such as the specific medication error and time-scale considered and that there was a *'very wide range of possible outcomes of omitting medicines, unintentionally or intentionally*'. **(P10 Ward Clinical Pharmacist)**

Some participants considered the potential for adverse effects as a longer-term risk and would not be apparent until sometime after the hospital discharge (e.g. unintentional discontinuation of

For peer review only - http://bmjopen.bmj.com/site/about/guidelines.xhtml

secondary prevention medication post-myocardial infarction or in the case below of continuation of anti-psychotic medication).

We had […] a case about ten years ago where a patient was prescribed three different medicines for delirium, different psychiatric medicines while they were on unit, then discharged to the ward, went home and then eventually they went back to their GP, was asked to sign their repeat prescription three months later and he looked at it and said, I didn't know this patient was under psychiatric care. And then looked into it and they'd basically never been stopped, the patient went home on anti-psychotic medicines, nobody stopped him. **(P11 Critical Care Pharmacist)**

Units that had undertaken a review on incidents, clinical audits or operated follow up clinics appeared more cognisant of potential medication issues after ICU patient transfer and implications for patient recovery. Some participants reported unit follow up of clinical incidents related to adverse drug events had been undertaken. Others had undertaken clinical audits or service evaluations of medication continuity and safety, which increased staff awareness of the high rates of medication errors on transition from ICU.

Yeah, 'cause we have had a couple of incidents and we've learned from them, and in fact our pharmacist did an audit on it, and presented that audit to the MDT, and there was a lot of learning points from that, and it has brought to us the importance, 'cause I think people just didn't, it just wasn't very high up in people's concerns about a patient. **(P12 ACCP)**

Feedback from follow up clinics were another way staff learnt about medication errors on transition and patient adverse drug events. These safety culture processes reduced the chance of siloed ICU perspectives on the patient care pathway.

Patient and family engagement

Participants identified that ICU patients frequently experienced delirium and memory loss which was a challenge to potential engagement in medication discussions and information whilst in ICU. Concerns about lack of patient recollection was a barrier to healthcare professional communication around medication handover on planned transfer to the hospital ward. It was unclear what perceived role family members had in this scenario. Whilst in ICU, the care team tended to take over all control of patient medication.

..we take complete control of their medications, nines time out of ten in hospital, so we are, you know, we are basically putting it all in our hands, and responsibility therefore. **(P9 ICU Consultant)**

For peer review only - http://bmjopen.bmj.com/site/about/guidelines.xhtml

Patient involvement in their medication through guiding discussions and questioning changes, was highlighted when their condition improved. Consequently, the role of the patient and family in medication safety and continuity whilst in ICU appeared passive, rather than being active agents.

And usually you find that a patient who's got long term illness, they're usually quite good at questioning medication. I can foresee that patients on intensive care, they've got poor concentration, attention, and they'll be coming to that remit of, the nurse [...] gave me six tablets, I'll just take them without asking any questions, et cetera. **(P1 Outreach Team)**

For peer review only

Discussion

This is the first qualitative research study focused on understanding the socio-technical factors of medication safety in patients transitioning from ICU. We identified thirteen factors within five broad themes, providing an interactive description of the factors that most strongly influenced the system performance. By highlighting the important system factors at an ICU and wider organisational levels, we provide a focus for service improvement efforts. Notably, time pressure considerations (workload, staffing, shift-work, out of hours transfers, bed availability) have a marked negative effect on the system performance. We now discuss how these factors impacted on team and perceived system performance in relation to the key socio-organisational and work system components identified [26].

Care team factors. We saw diverse multiprofessional staff groups, working in ICU and hospital-ward teams, were agents in the delivery of medication continuity and safety on ICU patient transfer. The performance of care teams was affected by staff knowledge and skills in medication review and prescribing, being perceived to vary across healthcare professional groups. Here, the core stability of some ICU staff groups, benefitted their practice and experience, over staff with regular clinical rotations. Importantly, limitations in understanding of the potential impact of medication errors on patient or organisation outcomes undermined the meaning of mitigating tasks or processes undertaken, particularly amongst rotational staff groups. Ultimately these limitations in ICU and ward-based staff beliefs about consequences of medication errors on this care interface present a challenge to the success of future behaviour change interventions [38].

We identified that the patient and family were often considered co-agents by ICU staff, reducing their engagement. Barriers to agent status included delirium and memory loss for patients. These findings may explain some of the limited patient awareness of medication changes identified on follow up [12]. Nevertheless, lack of communication and family support may also be factors [2]. Engaging patients and family members around safety of medication in transitions in care is recommended [15], and there are opportunities, such as written information communication [39], to support the resilience and adaptability exhibited by patients and family in recovery [2], and care satisfaction [29].

Task factors. The logistical challenges of patient flow on the transition from ICU to the hospital ward are well known [29,40]. We show that medication safety for ICU patients transitioning to a hospital ward is a complex process, involving complex patients, following an uncertain care pathway, involving diverse multiprofessional healthcare staff groups and personnel organised in multiple teams with varying resources. Process complexity was further increased by being time critical and

For peer review only - http://bmjopen.bmj.com/site/about/guidelines.xhtml

dependent. Task prioritisation was focused on the more acutely ill patients in ICU, or new patients on the hospital ward, potentially affected by staff resources and physical environment factors (ICU bed availability and pressures for beds) [41]. We found some medical staff considered the medication elements were a low priority to include in ICU patient transfer and handover plans, as previously reported by others [29]. In contrast, several medication-related elements (pre-admission medicines, active medications on transfer, rationale for medication regime and intravenous infusions) were considered essential information elements by ICU and ward-based Canadian medical staff [42].

Tools and technologies factors. We report that the full integration of e-prescribing systems across ICU and the hospital ward was clearly important in reducing medication transcription errors. Progression from non-integrated to integrated ICU and hospital ward e-prescribing systems, reduced the frequency and clinical severity of medication errors on transition in ICU patient care in an Australian report [43]. Although, it was also apparent that re-prescribing on transfer provided an opportunity to prompt a structured medication review, previously being associated with reduced risk of medication transfer errors [18]. Communication of information was viewed as supported when it was easily accessible and searchable within the numerous and complicated electronic system menus. The structure and content of the medication-related communication was aided by transfer checklists and summary reports system functionality.

Organisational factors. ICU leadership was provided by the ICU consultant and it was apparent from ICU participants that team performance, as exemplified in multiprofessional ward rounds was supported by psychological safety. This is reassuring given that ward round perception on quality of leadership predicts open multiprofessional communication within ICU [44]. Indeed, ICUs with a multiprofessional ward round are associated with a marked reduction in medication transfer errors [18]. However, ICU team performance is also informed by the composition and interaction of the multiprofessional healthcare team, providing diversity of specialism working on a co-ordinated and goal orientated manner [45]. Although we identified clear professional diversity, there were also indications of opportunities to further improve team effectiveness and performance by strengthening other components (dependability, structure and clarity, meaning, impact) [46].

Communication, particularly inter-team (ICU to Ward) communication, was considered a challenge to medication safety and continuity. These staff communication challenges around patient transfer from ICU to a hospital ward are well described elsewhere [29,47,48]. Limitations in written communication content are common, lacking such as decision-making information such as the rationale for medications that were started or stopped [48]. Lack of communication about

For peer review only

For peer review only - http://bmjopen.bmj.com/site/about/guidelines.xhtml

medication may contribute to medication errors in ICU survivors on the hospital ward [47], and inadequate handover can contribute to the distress patients experience on this transition in care interface [47]. We identified several factors that facilitated communication of medication related information. These included multiprofessional handovers, prescription annotations, outreach teams following up patients and plans, and referrals to specialist teams. Previous systematic reviews [28,49], have identified the importance of handover forms and liaison nurses, improving continuity or care but without any evidence of improved patient outcomes. However, it is not enough that a handover occurred, but that the right information is conveyed, acknowledged and actioned. Automation of facets of medication-related information via technological systems improve information transfer and delivery on ICU patient transitions [50].

Time pressures and considerations affected organisational conditions with time constraints having a notable effect on system performance. The important impact of such time considerations as facilitators and barriers to care during discharge from the ICU have been recently summarised [51]. Patients transferred out of hours from ICUs have worse outcomes, [52] and it is conceivable that medication safety issues contribute to this increased risk.

Our results share consistent themes previously identified in ICU transfers and medication or care continuity. Lack of staff knowledge, unclear task allocation and staff collaboration are a barrier to medication safety on transitions in patient care, as reported for medicines reconciliation processes [53]. Variation and lack of clarity of roles around inpatient medication reviews has previously been identified as an additional risk factor for medication errors, as reported for ward-based clinical pharmacists [54]. Patient severity (complexity) of illness, provider experience (knowledge and skills) and resource constraints (workload and staff availability), have previously been identified as themes affecting ICU patient discharge and readmission risk [55].

Strengths and Limitations

Our study had several strengths. Our findings are strengthened by the inclusion of views of UK healthcare professionals from both the ICU (transferring) and the hospital ward (receiving) multicentre teams, involved in the medication review and prescribing of ICU patients. Views represented teaching and district general hospitals, all of which use electronic prescribing systems. Thematic analysis was framed on the SEIPS 3.0 model, [26] which was developed specifically for evaluating the human factors of patient safety during transitions in care. We provide system interface interactions, including indications of modifiability to assist service improvement endeavours.

For peer review only

We did not include nursing staff as participants as the focus was medication review and prescribing activities, although ACCP and Outreach Teams are primarily from professional nursing backgrounds, making it likely we captured the main themes here as well. One interview of a hospital junior doctor failed to record properly. The potential impact was minimised by use of interviewer field notes (main points summary), and we benefitted from other participant interviews from the same profession. AAA is a critical care pharmacist and there is potential for bias and positionality to influence the interpretation of the results. We are confident that any risk of these were minimised by a non-ICU healthcare professional (BBB) leading the interviews, frequent reflexive evaluation, undertaking of dual coding of all interviews, and using an iterative process to identify the themes and sub-themes based on submersion in the data and continuous matching of extracts to the thematic analysis.

Recommendations for policy change and further research

Further research is required to confirm the relationship between medication transfer errors and adverse drug events [16], with ICU patient outcomes. Such evidence would inform staff beliefs about consequences, underpinning the education of staff on the clinical importance and how systems can be used to minimise human factor errors in this interface of care. Education should target ICU and ward-based healthcare staff on the common errors that occur with continuation of inappropriate medication, loss of medication information and failure to restart clinically important long-term medication when clinically appropriate. Furthermore, this education needs to translate into ICU junior doctor competency assessments. Specific staff groups (critical care pharmacists and ACCPs), should be delegated medication review responsibilities for ICU patients prior to hospital ward transition. All staff need to understand their responsibilities and those of colleagues. However, for clinical pharmacists to be able to take on routine delivery of these additional roles, they need to be dependable and ensure sufficient staff are available 7-days per week. Inter team communication processes around medication-information need to be streamlined, co-ordinated and accessible, with structured modes of delivery. ICU patients and/ or their family should be more engaged and involved in medication-related information, enabling more participation in their own treatment and recovery.

Conclusions

We identified important factors within five key themes that ICU and ward-healthcare professionals viewed were important in the safety and continuity of medication for ICU patients transitioning to a hospital ward. The complexity of the interactions on the performance and time dependency was clear. We make recommendations for policy change and further research based on improving: knowledge and skills of staff, sufficient critical care staffing, team performance, communication and collaboration, and patient and family engagement.

For peer review only - http://bmjopen.bmj.com/site/about/guidelines.xhtml

Acknowledgements

The authors would like to thank all the participants for taking the time to provide their insightful views that enriched the findings of this research.

Author Contributions

AAA, CCC and GGG were responsible for conception of this qualitative study. BBB undertook the participant interviews. AAA and BBB coded the transcripts and undertook the thematic analysis. AAA, CCC and GGG contributed to interpretating the results. AAA, BBB, CCC, DDD, EEE and FFF developed the representations of the work-system performance interactions. AAA & BBB wrote the manuscript. All authors read and approved the final manuscript. AAA acts as the guarantor for the intellectual integrity of the data.

Ethics Approval

Ethical (The University of Manchester, Ref: 2020-10852-17342) and NHS Health Research Authority (HRA) (IRAS 292456) approval was granted for the study.

Funding

This report is independent research supported by the National Institute for Health and Care Research NIHR300444. BBB and GGG are funded by the National Institute for Health and Care Research XXXXXX. The views expressed in this publication are those of the author(s) and not necessarily those of the NHS, the National Institute for Health and Care Research or the Department of Health and Social Care.

Competing interests

The authors declare that they have no competing interests.

Patient consent

Not applicable

Provenance and peer review

Not commissioned; externally peer reviewed

Data availability statement

The datasets used and/or analysed during the current study are available from the corresponding author on reasonable request.

Supporting Information

Supplementary file

For peer review only

For peer review only - http://bmjopen.bmj.com/site/about/guidelines.xhtml

Table S1. Electronic prescribing system configurations in ICU and hospital ward at each site

Table S2. Behavioural ICU transition medication error case vignettes

Interview Topic Guide

For peer review only - http://bmjopen.bmj.com/site/about/guidelines.xhtml
For peer review only

References

1 Rousseau AF, Prescott HC, Brett SJ, *et al.* Long-term outcomes after critical illness: recent insights. *Crit Care* 2021;**25**:1–7. doi:10.1186/s13054-021-03535-3

2 Haines KJ, Hibbert E, Leggett N, *et al.* Transitions of Care After Critical Illness-Challenges to Recovery and Adaptive Problem Solving. *Crit Care Med* 2021;**49**:1923–31. doi:10.1097/CCM.0000000000005095

3 Lone NI, Gillies MA, Haddow C, *et al.* Five-year mortality and hospital costs associated with surviving intensive care. *Am J Respir Crit Care Med* 2016;**194**:198–208. doi:10.1164/rccm.201511-2234OC

4 McPeake J, Bateson M, Christie F, *et al.* Hospital re-admission after critical care survival: a systematic review and meta-analysis. *Anaesthesia* 2022;**77**:475–85. doi:10.1111/anae.15644

5 Turnbull AJ, Donaghy E, Salisbury L, *et al.* Polypharmacy and emergency readmission to hospital after critical illness: a population-level cohort study. *Br J Anaesth* 2021;**126**:415–22. doi:10.1016/j.bja.2020.09.035

6 Brown SM, Bose S, Banner-Goodspeed V, *et al.* Approaches to addressing post-intensive care syndrome among intensive care unit survivors A Narrative Review. *Ann Am Thorac Soc* 2019;**16**:947–56. doi:10.1513/AnnalsATS.201812-913FR

7 Campbell AJ, Bloomfield R, Noble DW. An observational study of changes to long-term medication after admission to an intensive care unit. *Anaesthesia* 2006;**61**:1087–92. doi:10.1111/j.1365-2044.2006.04831.x

8 Bourne RS, Ioannides CP, Gillies CS, *et al.* Clinical frailty and polypharmacy in older emergency critical care patients: A single-centre retrospective case series. *Eur J Hosp Pharm* 2021;:ejhpharm-2020-002618. doi:10.1136/ejhpharm-2020-002618

9 Royal Pharmaceutical Society. Polypharmacy: Getting our medicines right. London: 2019. https://www.rpharms.com/recognition/setting-professional-standards/polypharmacy-getting-our-medicines-right

10 Scales DC, Fischer HD, Li P, *et al.* Unintentional Continuation of Medications Intended for Acute Illness After Hospital Discharge: A population-based cohort study. *J Gen Intern Med* 2016;**31**:196–202.

11 Bell CM, Brener SS, Gunraj N, *et al.* Association of ICU or hospital admission with unintentional discontinuation of medications for chronic diseases. *JAMA J Am Med Assoc* 2011;**306**:840–7.

12 MacTavish P, Quasim T, Purdie C, *et al.* Medication-related Problems in Intensive Care Unit Survivors: Learning from a Multicenter Program. *Ann Am Thorac Soc* 2020;**17**:1326–9. doi:10.1513/annalsats.202005-444rl

13 Stollings JL, Bloom SL, Wang L, *et al.* Critical Care Pharmacists and Medication Management in an ICU Recovery Center. *Ann Pharmacother* 2018;**52**:713–23. doi:10.1177/1060028018759343

14 Bosma LBE, van Rein N, Hunfeld NGM, *et al.* Development of a multivariable prediction model for identification of patients at risk for medication transfer errors at ICU discharge. *PLoS One* 2019;**14**:e0215459. doi:10.1371/journal.pone.0215459

15 World Health Organization. Medication Safety in Transitions of Care. 2019.

For peer review only - http://bmjopen.bmj.com/site/about/guidelines.xhtml

https://www.who.int/publications/i/item/WHO-UHC-SDS-2019.9

16 Bosma LBE, Hunfeld NGM, Quax RAM, *et al.* The effect of a medication reconciliation program in two intensive care units in the Netherlands: a prospective intervention study with a before and after design. *Ann Intensive Care* 2018;**8**:19. doi:10.1186/s13613-018-0361-2

17 Heselmans A, Van Krieken J, Cootjans S, *et al.* Medication review by a clinical pharmacist at the transfer point from ICU to ward: A randomized controlled trial. *J Clin Pharm Ther* 2015;**40**:578–83. doi:10.1111/jcpt.12314

18 Tully AP, Hammond DA, Li C, *et al.* Evaluation of Medication Errors at the Transition of Care From an ICU to Non-ICU Location. *Crit Care Med* 2019;**47**:543–9. doi:10.1097/CCM.0000000000003633

19 Sauro KM, Soo A, de Grood C, *et al.* Adverse Events After Transition From ICU to Hospital Ward. *Crit Care Med* 2020;**48**:946–53. doi:10.1097/CCM.0000000000004327

20 Fray M, Waterson P, Carman E. Care transitions in healthcare: The 'high-hanging fruit'. *Appl Ergon* 2021;**95**:103437. doi:10.1016/j.apergo.2021.103437

21 Co I, Hyzy RC. Lost in Transition: A Call to Arms for Better Transition From ICU to Hospital Ward. *Crit Care Med* 2020;**48**:1075–6. doi:10.1097/CCM.0000000000004381

22 Rhodes A, Moreno RP, Azoulay E, *et al.* Prospectively defined indicators to improve the safety and quality of care for critically ill patients: a report from the Task Force on Safety and Quality of the European Society of Intensive Care Medicine (ESICM). *Intensive Care Med* 2012;**38**:598–605.

23 Prescott HC, Angus DC. Enhancing recovery from sepsis: A review. *JAMA - J Am Med Assoc* 2018;**319**:62–75. doi:10.1001/jama.2017.17687

24 Bourne RS, Jennings JK, Panagioti M, *et al.* Medication-related interventions to improve medication safety and patient outcomes on transition from adult intensive care settings: a systematic review and meta-analysis. *BMJ Qual Saf* 2022;:bmjqs-2021-013760. doi:10.1136/bmjqs-2021-013760

25 Sevdalis N, Brett SJ. Improving care by understanding the way we work: human factors and behavioural science in the context of intensive care. *Crit Care* 2009;**13**:139. doi:10.1186/cc7787

26 Carayon P, Wooldridge A, Hoonakker P, *et al.* SEIPS 3.0: Human-centered design of the patient journey for patient safety. *Appl Ergon* 2020;**84**:103033. doi:10.1016/j.apergo.2019.103033

27 Donchin Y, Gopher D, Olin M, *et al.* A look into the nature and causes of human errors in the intensive care unit. *Qual Saf Health Care* 2003;**12**:294–300. doi:10.1136/qhc.12.2.143

28 van Sluisveld N, Hesselink G, van der Hoeven JG, *et al.* Improving clinical handover between intensive care unit and general ward professionals at intensive care unit discharge. *Intensive Care Med* 2015;**41**:589–604. doi:10.1007/s00134-015-3666-8

29 Stelfox HT, Leigh JP, Dodek PM, *et al.* A multi-center prospective cohort study of patient transfers from the intensive care unit to the hospital ward. *Intensive Care Med* 2017;**43**:1485–94. doi:10.1007/s00134-017-4910-1

30 Manias E, Williams A, Liew D. Interventions to reduce medication errors in adult intensive care: a systematic review. *Br J Clin Pharmacol* 2012;**74**:411–23.

For peer review only

For peer review only - http://bmjopen.bmj.com/site/about/guidelines.xhtml

31 Bueno WP, Saurin TA, Wachs P, *et al.* Coping with complexity in intensive care units: A systematic literature review of improvement interventions. *Saf Sci* 2019;**118**:814–25. doi:10.1016/j.ssci.2019.06.023

32 McNab D, McKay J, Shorrock S, *et al.* Development and application of systems thinking' principles for quality improvement. *BMJ Open Qual* 2020;**9**:e000714. doi:10.1136/bmjoq-2019-000714

33 Scanlon MC, Karsh BT. Value of human factors to medication and patient safety in the intensive care unit. *Crit Care Med* 2010;**38**:90–6. doi:10.1097/CCM.0b013e3181dd8de2

34 Carayon P, Wetterneck TB, Cartmill R, *et al.* Characterising the complexity of medication safety using a human factors approach: An observational study in two intensive care units. *BMJ Qual Saf* 2014;**23**:56–65. doi:10.1136/bmjqs-2013-001828

35 Braun V, Clarke V. To saturate or not to saturate? Questioning data saturation as a useful concept for thematic analysis and sample-size rationales. *Qual Res Sport Exerc Heal* 2021;**13**:201–16. doi:10.1080/2159676X.2019.1704846

36 Taylor-Adams S, Vincent C. Systems analysis of clinical incidents: the London protocol. *Clin Risk* 2004;**10**:211–20. doi:10.1258/1356262042368255

37 Reason J. *Human error*. Cambridge: : Cambridge University Press 1990. doi:doi:10.1017/CBO9781139062367

38 Carey RN, Connell LE, Johnston M, *et al.* Behavior Change Techniques and Their Mechanisms of Action: A Synthesis of Links Described in Published Intervention Literature. *Ann Behav Med* 2019;**53**:693–707. doi:10.1093/abm/kay078

39 Tobiano G, Chaboyer W, Teasdale T, *et al.* Older patient and family discharge medication communication: A mixed-methods study. *J Eval Clin Pract* 2021;**27**:898–906. doi:10.1111/jep.13494

40 Hua M, Wunsch H. The complexities of ICU discharge. *Chest* 2015;**147**:281–2. doi:10.1378/chest.14-2377

41 Batchelor A. Adult Critical Care GIRFT Programme National Specialty Report. 2021. https://www.gettingitrightfirsttime.co.uk/wp-content/uploads/2021/08/Adult-Critical-Care-Aug21L.pdf

42 de Grood C, Job McIntosh C, Boyd JM, *et al.* Identifying essential elements to include in Intensive Care Unit to hospital ward transfer summaries: A consensus methodology. *J Crit Care* 2019;**49**:27–32. doi:10.1016/j.jcrc.2018.10.001

43 Dabliz R, Poon SK, Fairbrother G, *et al.* Medication safety improvements during care transitions in an Australian intensive care unit following implementation of an electronic medication management system. *Int J Med Inform* 2021;**145**:104325. doi:10.1016/j.ijmedinf.2020.104325

44 Reader TW, Flin R, Mearns K, *et al.* Interdisciplinary communication in the intensive care unit. *Br J Anaesth* 2007;**98**:347–52. doi:10.1093/bja/ael372

45 Ervin JN, Kahn JM, Cohen TR, *et al.* Teamwork in the intensive care unit. *Am Psychol* 2018;**73**:468–77. doi:10.1037/amp0000247

46 Google. Project aristotle. https://rework.withgoogle.com/print/guides/5721312655835136/

47 Field K, Prinjha S, Rowan K. 'One patient amongst many': A qualitative analysis of intensive

For peer review only

For peer review only - http://bmjopen.bmj.com/site/about/guidelines.xhtml

care unit patients' experiences of transferring to the general ward. *Crit Care* 2008;**12**:21. doi:10.1186/cc6795

48 Brown KN, Leigh JP, Kamran H, *et al.* Transfers from intensive care unit to hospital ward: A multicentre textual analysis of physician progress notes. *Crit Care* 2018;**22**. doi:10.1186/s13054-018-1941-0

49 Wibrandt I, Lippert A. Improving Patient Safety in Handover From Intensive Care Unit to General Ward: A Systematic Review. *J Patient Saf* 2020;**16**:199–210. doi:10.1097/PTS.0000000000000266

50 Parsons Leigh J, Brundin-Mather R, Zjadewicz K, *et al.* Improving transitions in care from intensive care units: Development and pilot testing of an electronic communication tool for healthcare providers. *J Crit Care* 2020;**56**:265–72. doi:10.1016/j.jcrc.2020.01.019

51 Plotnikoff KM, Krewulak KD, Hernández L, *et al.* Patient discharge from intensive care: an updated scoping review to identify tools and practices to inform high-quality care. *Crit Care* 2021;**25**:1–13. doi:10.1186/s13054-021-03857-2

52 Vollam S, Dutton S, Lamb S, *et al.* Out-of-hours discharge from intensive care, in-hospital mortality and intensive care readmission rates: a systematic review and meta-analysis. *Intensive Care Med* 2018;**44**:1115–29. doi:10.1007/s00134-018-5245-2

53 van Sluisveld N, Zegers M, Natsch S, *et al.* Medication reconciliation at hospital admission and discharge: insufficient knowledge, unclear task reallocation and lack of collaboration as major barriers to medication safety. *BMC Health Serv Res* 2012;**12**:170. doi:10.1186/1472-6963-12-170

54 The role of clinical pharmacy services in helping to identify and reduce high-risk prescribing errors in hospital. 2020. https://www.hsib.org.uk/documents/249/hsib-report-high-risk-prescribing-errors-hospital.pdf

55 Ofoma UR, Dong Y, Gajic O, *et al.* A qualitative exploration of the discharge process and factors predisposing to readmissions to the intensive care unit. *BMC Health Serv Res* 2018;**18**:6. doi:10.1186/s12913-017-2821-z

For peer review only

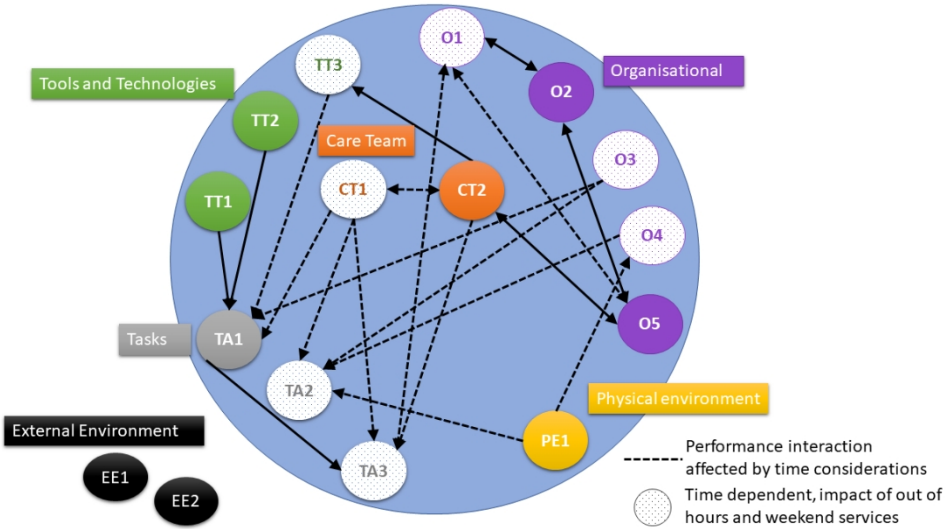

Fig 1: ICU patient transfer performance interaction

338x190mm (96 x 96 DPI)

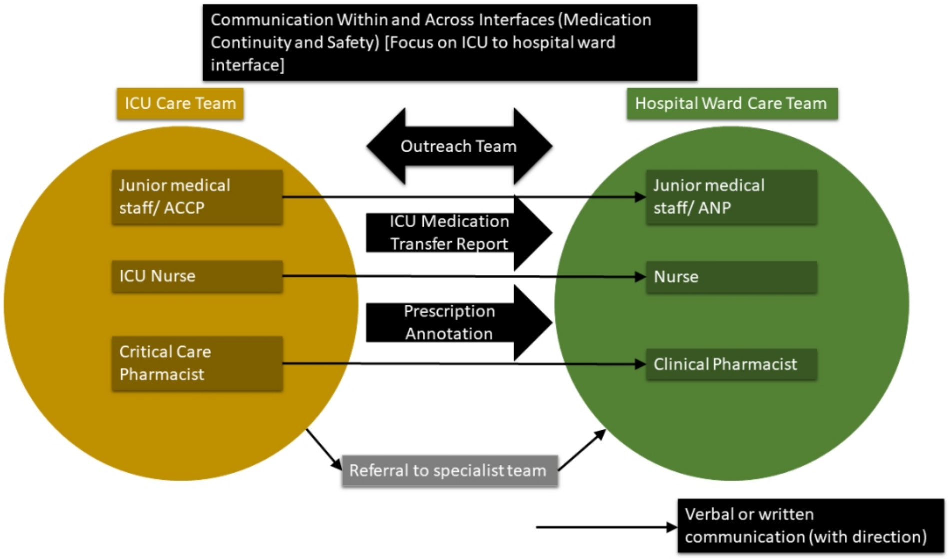

Fig 2: Medication continuity and safety communication across ICU to hospital ward interfaces

338x190mm (96 x 96 DPI)

Supplementary File

The multicentre study was conducted in 4 general intensive care units (ICUs) across 3 Trusts in the north of England (XXX (XXX NHSFT), YYY (YYY NHST), The AAA and BBB Hospital (both ZZZ NHS Trust)).

Centre	Hospital Type	ICU	Ward	Comment
XXX	Teaching	e-prescribing (MetaVision, iMDsoft)	e-prescribing (Lorenzo, DXC Technology)	No inter-system electronic communication. All ward-based infusion prescriptions on paper
YYY	Teaching	e-prescribing (MedChart)	e-prescribing (MedChart)	Inter-system communication. Complex and variable rate infusions are prescribed on paper
AAA	District General	e-prescribing (MedChart)	e-prescribing (MedChart)	Inter-system communication. Complex and variable rate infusions are prescribed on paper
BBB	Teaching	e-prescribing (Allscripts)	e-prescribing (Allscripts)	Inter-system communication. Total parenteral nutrition (TPN) prescribed on paper

Table S1. Electronic prescribing system configurations in ICU and hospital ward at each site

**Case 1 (slip and lapse type mistake)**
Error: Missed prescription for an antihypertensive drug (losartan) on ICU and hospital discharge, with that patient being hypertensive on return to GP practice.
Contextual factors: The losartan had not been prescribed in ICU as the patient had initially been hypotensive. On ICU discharge the patient was normotensive. The ICU was busy and only the medicines the patient was currently receiving whilst on ICU were transferred to the ward electronic prescription system. Losartan was on the ICU pre-admission

For peer review only - http://bmjopen.bmj.com/site/about/guidelines.xhtml

medication record, but this list was not transferred to the ward electronic system and the medicines reconciliation paper report was not reviewed on the ward.
**Case 2 (rules-based mistake)**
Error: ICU patient with Type 1 diabetes had their blood glucose controlled by a variable-rate insulin infusion. Their long-acting basal insulin was not prescribed whilst they were on a high-dose metaraminol infusion (as per ICU guidelines). The metaraminol was weaned off and the patient transitioned to the ward still without their basal SC insulin.
Contextual factors: Hospital guidelines for all Type 1 diabetic patients on a variable-rate insulin infusion is to continue their usual subcutaneous basal insulin to reduce the risk of diabetic ketoacidosis if the infusion is interrupted. ICU medical and pharmacy staff did not start the basal insulin after the metaraminol infusion was discontinued and this error was not identified prior to the ward transition. Only active medicines were transferred to the ward prescribing system. The Diabetes Specialist Team identified the error when they reviewed the patient on the ward.
**Case 3 (knowledge-based mistake)**
Error: An ICU patient receiving TPN was discharged to a ward without TPN prescribed on the paper fluid chart and didn't receive any TPN over the weekend (2 days).
Contextual factors: The trainee medical staff didn't know they needed to transfer the electronic TPN prescription from the ICU system to the paper fluid chart for the ward. The discharge was at the weekend and wasn't checked by a clinical pharmacist. Ward staff did not identify the patient was on TPN until the Nutrition Support Team reviewed the patient on Monday.

Table S2. Behavioural ICU transition medication error case vignettes

For peer review only

Interview Topic Guide

Interview questions:

Designation

1. What is your specific role at the hospital?

- How long have you been in this post?

Prior understanding of ICU patient transfer medication errors and local systems

2. Can you tell me about any prior awareness/knowledge/experience you have of any ICU patient transfer medication errors?

- If so, were these due to an error with medication review, or prescribing or both?
- In your opinion, do such errors significantly impact on patient care or not?
- Is that in the hospital or the community or both?

(Explore if this is primarily an ICU, or hospital word, or GP issue or does it traverse all of these areas?)

How are these errors usually detected?

3. Can you describe any procedures or safety systems are in place in your hospital to reduce medication errors on the interface of ICU patient transfers to the ward? *(*Is there an ICU discharge procedure? If so, please tell me about that discharge procedure? Do outreach services routinely review all patient's medicines on the ward?

4. How well are these procedures and safety systems implemented?

Who is responsible for the delivering the various safety systems mentioned?

Is there clear task allocation by profession or group?

How well are they adhered to in practice?

For peer review only

Case vignette discussions

- Case 1 (slip and lapse type mistake). Error and Contextual factors

So Case 1 is an error in which somebody forgets to do something they should or accidentally do something they shouldn't have done

5. Is this type of error common in your opinion?

How common? *[Rare to very common etc?]*

What do you think contributes most to slips and lapse type errors?

- Can signpost – London Protocol contributory influencing factors e.g. communication, workload, knowledge or skills if asked)

6. What things would help ro reduce errors like these?

   Then, if need be, prompt the participant to talk specifically about procedural/system/staff role contributions as required.

- Case 2 (rules-based mistake). Error and Contextual factors

So Case 2 is an error in which somebody incorrectly applies or doesn't apply a rule to a situation

What do you think contributes most to rule-based errors?

- Can signpost – London Protocol contributory influencing factors e.g. communication, workload, knowledge or skills if asked)

7. Is this type of error common in your opinion?
8. How common? *[Rare to very common etc?]*
9. What things would help to reduce errors like these?"

   Then, if need be, prompt the participant to talk specifically about procedural/system/staff role contributions as required.

- Case 3 (knowledge-based mistake). Error and Contextual factors

So case 3 is an error in which somebody misunderstands or misinterprets what is happening

What do you think contributes most to knowledge-based errors?

- Can signpost – London Protocol contributory influencing factors e.g. communication, workload, knowledge or skills if asked)

10. Is this type of error common in your opinion?
11. How common? *[Rare to very common etc?]*
12. What things would help ro reduce errors like these?"

For peer review only - http://bmjopen.bmj.com/site/about/guidelines.xhtml

Then, if need be, prompt the participant to talk specifically about procedural/system/staff role contributions as required.

Which error(s) do you most frequently encounter?

(Thinking about these errors in general; what is your role in mitigating the risk of these happening?

What could your role be?)

Facilitators and barriers to medication review (ICU patients on ward transfer)

Thinking about medication review for ICU patients during transfer to a ward:

How well does this process work?

What helps you to do it effectively?

What problems do you encounter?"

[*You can use the SEIPS framework to probe the participant for specific areas as required*]

Facilitators and barriers to safe prescribing and monitoring (ICU patients on ward transfer)

Thinking about prescribing and monitoring of medicines on the interface of ICU ward transfer:

How well does this process work?

What helps you to do it effectively?

What problems do you encounter?"

[You can use the SEIPS framework to probe the participant for specific areas as required]

Reducing medication review and/or medication errors on the interface of ICU patient transfer to a ward

13. If you could change one thing to improve the safety and continuity of medication on the ICU to ward interface – what would that be?
14. Have you any other comments or observations on the topic of medication continuity or safety in ICU patients on the interface of ward transfer?
15. Thank you for your time and helpful views.

For peer review only - http://bmjopen.bmj.com/site/about/guidelines.xhtml

Factor Types	Contributory influencing factor
Patient factors	Condition (complexity and seriousness)<br>Language and communication<br>Personality and social factors
Task and<br>technology factors	Task design and clarity of structure<br>Availability and use of protocols<br>Availability and accuracy of test results<br>Decision-making aids
Individual (staff) factors	Knowledge and skills<br>Competence<br>Physical and mental health
Team factors	Verbal communication<br>Written communication<br>Supervision and seeking help<br>Team structure (congruence, consistency, leadership etc.)
Work environmental factors	Staffing levels and skills mix<br>Workload and shift patterns factors<br>Design, availability and maintenance of equipment<br>Administrative and managerial support<br>Environment<br>Physical
Organisation & management factors	Financial resources and constraints<br>Organisational structure factors<br>Policy, standards and goals<br>Safety culture and priorities
Institutional context factors	Economic and regulatory context<br>National health service executive<br>Links with external organisations

London Protocol: Framework of contributory factors influencing clinical practice

For peer review only

**Work System**	Team members
	Tasks
	Technologies and tools
	Organisation
	Physical environment
	External environment
**Process ("Medication Review & prescribing")**	Information gathering
	Information integration & Interpretation
	Medication plan
	Communication of the plan
	Treatment (Medication received & monitoring)
**Outcomes**	Patient (Adverse events, increased length of hospital stay, readmission to ICU or hospital, death)
	System (Effects on quality, safety, cost, efficiency, morale, public confidence in the health system)
	Learning from medication errors

SEIPS 3.0 Framework

For peer review only

For peer review only - http://bmjopen.bmj.com/site/about/guidelines.xhtml

Understanding medication safety involving patient transfer from intensive care to hospital ward: A qualitative socio-technical factors study

COREQ checklist

Note: in order to minimize the length of the manuscript, some of the details on the checklist (marked '*') are not included in the manuscript.

For peer review only

Guide question		*Response*	*Page number in manuscript*
1	Interviewer/facilitator	MJ conducted the interviews.	Methods Page 5
2	Credentials	MJ holds an PhD in Pharmacy Practice	Title page
3	Occupation	MJ: Research Associate in medication safety; RSB is a consultant pharmacist	Title page
4	Experience and training	MJ has extensive previous experience of undertaking qualitative research related to medication optimisation and safety in NHS healthcare environments	Methods Page 5
5	Relationship established	MJ was not known to the participants prior to the study. RB knew some of the researchers through professional networks or as co-workers	*
6	Participant knowledge of the research	Participants were made aware of the reasons for doing the research via the information which was given to the participant prior to the interview	Methods Page 4
7	Research characteristics	The researchers had identified the study topic based on a systematic review and meta-analysis they had undertaken of medication-related interventions to impact on medication safety for ICU patients transitioning to the hospital ward. The results of the systematic review identified the lack of theory informed interventions. The human factors interviews were part of a series of studies designed to identify and develop a theory informed intervention package suitable for testing in a future randomised controlled trial.	*
8	Methodological orientation and theory	London Protocol; Systems Engineering for Patient Safety (SEIPS) 3.0 Model	Methods Page 4
9	Sampling	We recruited from a purposive sample of healthcare professionals across four NHS centres chosen to represent both District General and Teaching Hospitals in the north of England. The sampling frame was Intensive Care Units (ICU) and ward-based healthcare professionals. These included medical staff, advanced critical care practitioners (ACCP), critical care	Methods Page 4

		pharmacists, ward-based clinical pharmacists and ICU outreach staff.	
10	Method of approach	Potential participants were identified via the Principal Investigators at each NHS hospital. Potential participants were invited to participate by email. Emails were directed by departmental leads or departmental email distribution lists.	Methods Page 4
12	Sample size	22 participants.	Results page 5 and Table 1
13	Non-participation	A small number of possible participants were approached by the PIs but declined to participate. This was predominantly this was for reasons of time, workload or lack of use of the system. Twenty-four participants consented, of which it was not possible to arrange an interview for 2 of these in a timely manner. Twenty-two participants were interviewed.	*
14	Setting of data collection	All interviews were conducted by MJ and took place via video telecommunication software (Microsoft Teams, Microsoft Corporation, USA)	Methods Page 4
15	Presence of non-participants	No non-participants were present	*
16	Description of sample	Twenty-two ICU and ward-based healthcare professionals were interviewed (3 ICU medical staff, 4 ACCP, 4 Critical Care Pharmacists, 4 Outreach Team Members, 3 Ward Medical Staff, 4 Ward Clinical Pharmacists.	Results Page 5 and Table 1
17	Interview guide	The semi-structured interview schedule was informed by London Protocol and Systems Engineering for Patient Safety (SEIPS) 3.0 Model. These facilitated capture the process, contributing factors and context of medication safety at ICU to ward transition. In order to prompt discussion, we created three medication safety error case vignettes each representing a type of error. The vignettes were shared with participants in advance of the interview.	Methods Page 4
18	Repeat interviews	No follow-up interviews were conducted	
19	Audio/visual recording	Interviews took place via video telecommunication software (Microsoft Teams, Microsoft Corporation, USA) with consent from the participant.	Methods Page 4
20	Field notes	None	Strengths and Limitations Page 21
21	Duration	Interviews ranged in duration from 30-67 minutes with a mean length of 49 minutes.	*
22	Data saturation	Data collection continued until saturation was reached and no new themes emerged from the interviews.	*
23	Transcripts returned	No transcripts were returned to participants	*
24	Number of data coders	Coding was undertaken in duplicate by the qualitative researcher (MJ) and Chief Investigator (RSB)	Methods Page 5
25	Description of the coding tree	Coding was informed by London Protocol and SEIPS 3.0 model frameworks. Coding was completed in three rounds with discussions between the coders after each round	Methods Page 5
26	Derivation of themes	We identified 13 factors in five broad themes. The thematic analysis was then developed to provide a detailed description of the ICU-hospital work-system interface	Results Page 5 Results page 16, Table 2,

For peer review only

			Figures 1 &2
27	Software	QSRNvivo 12 software was utilised to manage the data	Methods Page 5
28	Participant checking	No	*
29	Quotations presented	Please see the results section of the manuscript	Results Page 5-16, Table 2, Figures 1 &2
30	Data and findings consistent		
31	Clarity of major themes		
32	Clarity of minor themes		

For peer review only

BMJ Open

Understanding medication safety involving patient transfer from intensive care to hospital ward: A qualitative socio-technical factors study

Journal:	*BMJ Open*
Manuscript ID	bmjopen-2022-066757.R1
Article Type:	Original research
Date Submitted by the Author:	28-Feb-2023
Complete List of Authors:	Bourne, Richard; Sheffield Teaching Hospitals NHS Foundation Trust, Departments of Pharmacy and Critical Care; The University of Manchester, Division of Pharmacy and Optometry, School of Health Sciences<br>Jeffries, Mark; University of Manchester, Division of Pharmacy and Optometry, School of Health Sciences; NIHR Greater Manchester Patient Safety Translational Research Centre<br>Phipps, Denham; The University of Manchester, Division of Pharmacy and Optometry, School of Health Sciences<br>Jennings, Jennifer K; Sheffield Teaching Hospitals NHS Foundation Trust, Departments of Pharmacy and Critical Care<br>Boxall, Emma; Northern Care Alliance NHS Foundation Trust, Salford Royal Hospital, Pharmacy Department<br>Wilson, Franki; Leeds Teaching Hospitals NHS Trust, Pharmacy Department<br>March, Helen; Northern Care Alliance NHS Foundation Trust, Royal Oldham Hospital, Pharmacy Department<br>Ashcroft, Darren; The University of Manchester, Division of Pharmacy and Optometry, School of Health Sciences; NIHR Greater Manchester Patient Safety Translational Research Centre
**Primary Subject Heading**:	Qualitative research
Secondary Subject Heading:	Intensive care, Health services research, Evidence based practice
Keywords:	Adult intensive & critical care < INTENSIVE & CRITICAL CARE, Health & safety < HEALTH SERVICES ADMINISTRATION & MANAGEMENT, Quality in health care < HEALTH SERVICES ADMINISTRATION & MANAGEMENT, QUALITATIVE RESEARCH

SCHOLARONE™
Manuscripts

I, the Submitting Author has the right to grant and does grant on behalf of all authors of the Work (as defined in the below author licence), an exclusive licence and/or a non-exclusive licence for contributions from authors who are: i) UK Crown employees; ii) where BMJ has agreed a CC-BY licence shall apply, and/or iii) in accordance with the terms applicable for US Federal Government officers or employees acting as part of their official duties; on a worldwide, perpetual, irrevocable, royalty-free basis to BMJ Publishing Group Ltd ("BMJ") its licensees and where the relevant Journal is co-owned by BMJ to the co-owners of the Journal, to publish the Work in this journal and any other BMJ products and to exploit all rights, as set out in our licence.

The Submitting Author accepts and understands that any supply made under these terms is made by BMJ to the Submitting Author unless you are acting as an employee on behalf of your employer or a postgraduate student of an affiliated institution which is paying any applicable article publishing charge ("APC") for Open Access articles. Where the Submitting Author wishes to make the Work available on an Open Access basis (and intends to pay the relevant APC), the terms of reuse of such Open Access shall be governed by a Creative Commons licence – details of these licences and which Creative Commons licence will apply to this Work are set out in our licence referred to above.

Other than as permitted in any relevant BMJ Author's Self Archiving Policies, I confirm this Work has not been accepted for publication elsewhere, is not being considered for publication elsewhere and does not duplicate material already published. I confirm all authors consent to publication of this Work and authorise the granting of this licence.

For peer review only

Understanding medication safety involving patient transfer from intensive care to hospital ward: A qualitative socio-technical factors study

Richard S Bourne[1,2,3*], Mark Jeffries[2,3], Denham L Phipps[2], Jennifer K Jennings[1], Emma Boxall[4], Franki Wilson[5], Helen March[6], Darren M Ashcroft[2,3]

[1] Departments of Pharmacy and Critical Care, Sheffield Teaching Hospitals NHS Foundation Trust, Sheffield, UK

[2] Division of Pharmacy and Optometry, School of Health Sciences, Faculty of Biology, Medicine and Health, The University of Manchester, Manchester, UK

[3] National Institute for Health and Care Research (NIHR) Greater Manchester Patient Safety Translational Research Centre (PSTRC), School of Health Sciences, Faculty of Biology, Medicine and Health, The University of Manchester, Manchester, UK

[4] Pharmacy Department, Salford Royal Hospital, Northern Care Alliance NHS Group, Salford, UK

[5] Pharmacy Department, Leeds Teaching Hospitals NHS Trust, Leeds, UK

[6] Pharmacy Department, Royal Oldham Hospital, Northern Care Alliance NHS Group, Oldham, UK

*Corresponding author

E-mail: richard.bourne1@nhs.net

Word count: 7146

Keywords: Critical Care, Medication Errors, Patient Safety, Systems Analysis, Transitions

For peer review only

Abstract

Objective

To understand the socio-technical factors affecting medication safety when intensive care patients are transferred to a hospital ward. Consideration of these medication safety factors would provide a theoretical basis, on which future interventions can be developed and evaluated to improve patient care.

Design

Qualitative study using semi-structured interviews of intensive care and hospital ward-based healthcare professionals. Transcripts were anonymised prior to thematic analysis using the London Protocol and Systems Engineering in Patient Safety 3.0 model frameworks.

Setting

Four north of England National Health Service hospitals. All hospitals used electronic prescribing in intensive care and hospital ward settings.

Participants

Intensive care and hospital ward healthcare professionals (intensive care medical staff, advanced practitioners, pharmacists and outreach team members; ward-based medical staff and clinical pharmacists).

Results

Twenty-two healthcare professionals were interviewed. We identified thirteen factors within five broad themes, describing the interactions that most strongly influenced the performance of the intensive care to hospital ward system interface. The themes were: Complexity of process performance and interactions; Time pressures and considerations; Communication processes and

For peer review only

For peer review only - http://bmjopen.bmj.com/site/about/guidelines.xhtml

challenges; Technology and systems; and Beliefs about consequences for the patient and organisation.

Conclusions

The complexity of the interactions on the system performance and time dependency was clear. We make several recommendations for policy change and further research based on improving: availability of hospital-wide integrated and functional electronic prescribing systems, patient flow systems, sufficient multiprofessional critical care staffing, knowledge and skills of staff, team performance, communication and collaboration, and patient and family engagement.

Strengths and limitations of this study

- Medication safety for intensive care patients transitioning to a hospital ward requires co-ordination of care within and across multiprofessional teams and environments with varying staff and technological resources.
- This is the first qualitative study focused on understanding the socio-technical factors of medication safety in patients transitioning from intensive care to a hospital ward.
- Study strengths include multicentre views of intensive care and hospital ward UK healthcare professionals, representing teaching and district general hospitals, all using electronic prescribing systems.
- Thematic analysis using the Systems Engineering Initiative for Patient Safety (SEIPS 3.0) model enabled evaluation of the socio-technical factors impacting on medication safety during transition from intensive care to a hospital ward.
- We report on findings from four trusts from England, and findings cannot be generalised to other settings outside the UK.

For peer review only

Background

Patients recovering from a critical illness requiring intensive care unit (ICU) treatment are generally transferred to a hospital ward before eventual hospital discharge to the community. However, this simplified summary underrepresents the complexity of the patient pathway, where full patient recovery may be protracted, traversing hospital and community care and involve several care transitions [1,2]. Compared to patients discharged from standard hospital care, ICU patients have worse short and medium-term outcomes, requiring additional ongoing healthcare resources [3]. Half of ICU patients require emergency hospital readmission within a year of hospital discharge [4]. This hospital readmission risk is predicted by pre-existing patient multimorbidity, frailty and polypharmacy [4,5]. There is a growing appreciation of the need to improve the recovery experience of ICU survivors, including reducing complications related to polypharmacy and care fragmentation [1,6]. Transitions in care are known to increase patient risks, including those related to medicines usage and care continuity [7]. The transition from ICU to a hospital ward is a marked interface in patient care, with contrasting resources in care team and technological support available in the respective clinical areas. Transition from ICU exposes patients to high rates of medication errors (MEs) [8–10], and adverse events are common, including those that are medication-related [11]. Extensive and frequent medication changes are made during a patient's ICU episode [12,13], increasing the risks for hazardous polypharmacy on care transitions [14]. The most common MEs centre around failure to discontinue inappropriate acute medication without an ongoing indication and not restarting clinically important long-term medication when safe to do so [8–10,15]. Such MEs may remain uncorrected beyond the acute hospital care episode [16–19], and be potentiated by pre-existing polypharmacy [20]. ICU care may therefore compound the known complexities and challenges for patient medication care created by care transitions [7,21].

[22] Organisational recommendations to support the continuity of care, including mitigation of these increased ME risks, have centred around structured staff handovers between ICU and the hospital

For peer review only - http://bmjopen.bmj.com/site/about/guidelines.xhtml

wardto support the continuity of care including a focus on medication [23]. Broader indicators of interventions that can improve medication safety on transitions in patient care are provided by the World Health Organization (WHO) and ICU expert views [7,24]. To date interventions to improve the safety and continuity of medication for ICU patients transitioning to a hospital ward have focused on education of staff, medication review, guidelines, electronic transfer/ handover tool or letter and medicines reconciliation [25]. Simple interventions, based on staff education and guidelines, have been demonstrated to reduce the risk of inappropriate continuation of acute medication no longer indicated [25]. The more complex, and higher clinic risk, of failure to restart clinically important long-term medication, will require a more complex intervention package [25–27].

For any intervention package to be effective in the ICU environment and improve patient care, it is important to understand how the ICU system works [28]. Furthermore, the care interfaces need to be examined in terms of their systems, processes and outcomes [29]. Error prevention in ICU care is dependent on effective inter and intra-multiprofessional team working and communication [30], that are particularly important on patient transition from ICU to the hospital ward [31,32]. Despite ICUs being technology rich environments, an over-reliance on technology to mitigate against medication errors underestimates the complexity of the systems and processes [33,34]. Like other parts of healthcare, ICU and its care transitions involve a complex interplay between technology, tasks and people [35]. Therefore, the importance of human factors engineering has been promoted to address the whole ICU medication management and patient safety pathway [30,36,37].

This study aimed to understand the socio-technical factors affecting medication safety when ICU patients are transferred to a hospital ward. Consideration of these medication safety factors would provide a theoretical basis, on which future interventions can be developed and evaluated to improve patient recovery.

For peer review only - http://bmjopen.bmj.com/site/about/guidelines.xhtml

Methods

Our study used a qualitative design, intended to solicit adult, general ICU and ward-based healthcare professionals directly involved in the prescribing and medication review of ICU patients on the interface of transition to the hospital ward. We recruited from a purposive sample of healthcare professionals across four NHS centres, chosen to represent both District General and Teaching Hospitals in the north of England with a representative range and combination of ICU and hospital ward e-prescribing systems (Supplementary File, Table S1). The sampling frame was ICU (medical staff, advanced critical care practitioners (ACCPs), critical care pharmacists and ICU outreach team members) and ward-based (medical staff, clinical pharmacists) healthcare professionals. Principal Investigators at each NHS hospital directed participant email invitations via departmental team leads or departmental email distribution lists. All participants provided informed written consent.

We undertook semi-structured interviews facilitated by a topic guide adapted from the London Protocol [38] and Systems Engineering Initiative for Patient Safety (SEIPS) 3.0 model [29] (Supplementary File, Topic guide). The London Protocol was developed from Reason's "Swiss Cheese" model, capturing the contributing factors and context around a specific patient safety incident [38]. The SEIPS models are based on the System-Process-Outcome description of approaches to measuring and improving the quality of health care. Version 3.0 of the SEIPS framework includes an expanded process component, focusing on the patient pathway to describe patient interactions with multiple care settings over time [29]. To prompt discussion, we selected three reported ICU medication error cases as vignettes, each representing a type of human error from Reason's (1990) generic error modelling system [39] (see Supplementary File, Table S2). The vignettes were shared with participants in advance of the scheduled interview. All interviews were undertaken via video telecommunication software (Microsoft Teams, Microsoft Corporation, USA), recorded and transcribed verbatim. A qualitative researcher (BBB) experienced in investigation of medicines optimisation practices in NHS healthcare environments conducted the interviews.

Transcripts were anonymised prior to deductive thematic analysis [40], using the London Protocol

For peer review only - http://bmjopen.bmj.com/site/about/guidelines.xhtml

[38], and SEIPS 3.0 model [29] frameworks. Coding was supported through qualitative research software (NVivo, Version 12, QSR International (UK) Limited) and undertaken in duplicate by the qualitative researcher (BBB) and Chief Investigator (AAA). All transcript content was coded using the thematic analysis. The coding was completed in three rounds with discussions between the coders after each round, and with the wider research team at different intervals. Data-saturation was considered to be achieved when we judged further interpretation of codes and themes would not provide further insights [41]. Finally, through multiple discussions amongst the research team, we identified main themes as being as those that provided the most meaningful pattern across the data set. We coalesced the main identified factors until they provided a stable and parsimonious account of the interactions that most strongly influence the performance of the ICU to hospital ward work system and communication pathways.

Patient and Public Involvement

We have actively engaged with patient and public involvement (PPI) in this wider research intervention development programme, to improve medication safety for intensive care patients transitioning to a hospital ward. In this study, PPI colleagues consulted on and contributed to the development of the topic guide, interpretation of the thematic findings and dissemination of results.

Ethical (The University of Manchester, Ref: 2020-10852-17342) and NHS Health Research Authority (HRA) (IRAS 292456) approval was granted for the study. We have adhered to the Consolidated Criteria for Reporting Qualitative Studies (COREQ) in reporting our findings [42].

For peer review only - http://bmjopen.bmj.com/site/about/guidelines.xhtml

Results

We recruited 22 participants representing each professional group and clinical location (see Table 1 for more details) [41].

Healthcare professional staff group	ICU Medical Staff	Advanced Critical Care Practitioners (ACCP)	Critical Care Pharmacists	Outreach Team Members	Ward Medical Staff	Ward Clinical Pharmacists
**n**	3*	4	4	4	3*	4
**Time in current role (years, mean (SD))**	2.7 (2.1)	6 (2.2)	11 (12.8)	12.3 (5.5)	2 (1)	1.5 (1.3)

Table 1: Healthcare professional participants (n=22)

* Participants from sites 1,3 and 4 only

We identified thirteen factors within five broad themes, providing a detailed description of the ICU-hospital work-system interface. This identified the complexity and interactions within the system and accounted for the significant impact of time pressures and considerations (Table 2, Fig 1). The vulnerability of the system to time constraints was evident (Fig 1). The communication pathways utilised are presented (Fig 2).

ICU-Hospital Ward Work System Interface
ICU Active Agents: ICU Consultant, ICU Junior medical staff, Advanced Critical Care Practitioner, Critical Care Pharmacist, ICU Nurse, Outreach Team, Microbiologist<br>*Hospital Ward Active Agents*: Junior medical staff, Specialist Teams (e.g. Diabetes team, Acute Pain Team), Clinical Pharmacist, Nurse, Outreach Team
**Performance is shaped most *strongly* by interactions of:**
Care Team Factor(s)<br>-CT1: Knowledge and skills of staff on medication review and safe prescribing for ICU patients on the interface of transition to the hospital ward* [Effect dependent on status]<br>-CT2: Beliefs about consequences, understanding of staff to the frequency and clinical impact of medication errors on patient recovery* [Positive effect]
Task Factor(s)<br>-TA1: Task and patient complexity affected by the number of long-term and acute medicines used in patients with a critical illness with unpredictable recovery and responses to treatment including medication [Negative effect]<br>-TA2: Task prioritisation, often focused on the new patient admission(s) or more unstable patient(s) in ICU, with medication use in ICU transfer patient deemed low priority* [Negative effect]<br>-TA3: Task allocation and delegation amongst the care team* [Effect dependent on status]
Tools and Technologies Factor(s)<br>-TT1: E-Prescribing system integration and functionality** [Effect dependent on status]<br>-TT2: Electronic patient notes accessibility and searchability** [Effect dependent on status]<br>-TT3: Medication transfer checklist completion and adherence* [Effect dependent on status]
Organisational Factor(s)

For peer review only - http://bmjopen.bmj.com/site/about/guidelines.xhtml

-O1: Team performance (organisation, leadership, collaboration and dependability)* [Effect dependent on status]<br>-O2: Multiprofessional ward rounds including a medication review element* [Positive effect]<br>-O3: Communication between ICU and Hospital Ward team members*/** [Effect dependent on status]<br>-O4: Time pressures in the need to complete the ICU patient transfer to the ward, staff workload and out of hours transfers*/** [Negative effect]<br>-O5: Safety culture in ICU informed by incident reviews, audit and feedback and follow up clinic experiences* [Positive effect]
Physical Environment Factor(s)<br>-PE1: ICU bed availability and pressure for beds** [Effect dependent on status]
External Environment Factor(s)<br>-EE1: Bed availability on the hospital ward and transfer times** [Effect dependent on status]<br>-EE2: Transfer time standards [Negative effect]
On ICU, Patient and Family co-agents*, also Dietitian, Nutrition support team, Visiting teams and Pharmacy Technician. On the Hospital Ward, the Rehabilitation Team were co-agents.

Table 2: ICU-Hospital Ward Work System Interface

*Potentially modifiable at a local ICU level; ** potentially modifiable at an organisational level

Fig 1: ICU patient transfer performance interaction

Fig 2: Medication continuity and safety communication across ICU to hospital ward interfaces

For peer review only

For peer review only - http://bmjopen.bmj.com/site/about/guidelines.xhtml

Theme 1. Complexity of process performance and interactions

Care Teams and Collaboration

A professionally diverse range of ICU and hospital ward-based healthcare staff undertook important tasks and processes to support medication safety for ICU patients transferring to a hospital ward (Table 2). In the ICU, participants emphasised the value of collaborative, multiprofessional team working that was driven by effective team leadership. ICU consultants led multiprofessional ward rounds, provided leadership and examples for more junior staff in the routine delivery of patient medication reviews. ACCP, medical and nursing staff had important roles in medication review. The role of the critical care pharmacist in medicines reconciliation on admission and undertaking medication reviews for patients, including during ward rounds and prior to hospital discharge was highlighted.

the MDT ward round is really useful, as the [..] consultant you're trying to pull a broad picture of everything together for discharge, and medications is one part of that, and I think we're very lucky in (name of hospital), the quality and the provision of our pharmacy support is excellent. [..] I don't see how we could do it without the pharmacists. **(P5 ICU Consultant)**

On the hospital ward, there was also evidence of multiprofessional team working in undertaking medication reviews in ICU patients transferred to the hospital ward. In some cases, these were facilitated by consultant-led ward rounds. Whilst direct collaboration between the ICU and hospital ward team appeared less clear, the ICU outreach team did provide an important link between the two clinical areas.

So I follow patients up post-discharge from intensive care […] to look at them physiologically, psychologically, and just follow the plan that's been put in place on a critical care area. […] Regarding medication, if there's something specific in the discharge plan, then I would review that aspect. **(P1 Outreach Team)**

On the hospital ward, referral to clinical pharmacists and specialist teams in undertaking medication reviews were highlighted. The diabetes team were seen as essential for patients receiving insulin therapy when transferred to a hospital ward and the acute pain team were often engaged with patients with ongoing pain issues.

But there's also other specialists that we get involved […] we should automatically make a diabetic team referral. So, it's also pulling in other specialist teams into the mix […] **(P3 ACCP)**

Tasks, processes and prioritisation

For peer review only - http://bmjopen.bmj.com/site/about/guidelines.xhtml

For peer review only

The key medication-related tasks undertaken by ICU healthcare professionals interviewed were medication review, prescribing and plan setting. These were supported by processes such as medicines reconciliation and, in some units, a transfer checklist that included medication elements. ICU transfer reports sometimes included medication information such as pre-admission medicines, current medication and medication plans. Transcribing and transcription checks were also undertaken in ICUs without integrated ICU and hospital ward electronic prescribing systems, which also provided a medication review opportunity.

I would say [the transcription process is] vitally important. So, part of the problem is […] it shouldn't really be seen as a transcription process, it should be more of a review process. […] it's not simply passing straight from one system to another including all that information, it's actually reviewing what's appropriate. **(P2 Critical Care Pharmacist)**

The complexity of tasks undertaken was qualified within the context of ICU patient care and regarded as impacting on medication safety throughout the patient journey. The acuity of the patient condition, and increasing number of medicines they received, contributed to medication review complexity.

we're now seeing patients who are more complex than ever before and on more medicines than ever before, and then, obviously, that becomes even more complex in itself. **(P9 ICU Consultant)**

Tasks were often prioritised, which sometimes meant that the medication-related tasks were seen as a low or lower priority and '*probably the least important thing you would do that day.'* **(P13 Ward Junior Doctor)**. The seriousness of the patient's condition determined the time spent on their care; more seriously ill patients were prioritised over the patient transferring from ICU.

And because it's quite a fast-paced unit those patients could be gone before I see them again because then I could be reviewing the critical unwell patients, the Level 3 patients, the ones that are intubated, and things. So, those patients that are then getting discharged are in the back of my mind because I'm dealing with the sicker ones. **(P14 ACCP)**

The priority for ward-based clinical pharmacists was often focused on undertaking medicines reconciliation for new hospital admissions and discharge prescriptions, over reviewing patients transferred from ICU. Whilst ward medical staff focused on critical medicines.

So I think when you're on the ward round and you go to review the medicines, you mainly look at things that are critical to the patient at the time first. So antibiotics, blood thinners and that sort of thing. And looking back to admission meds to see what's missing while they're still in hospital is not always top of your list. **(P20 Ward Junior Doctor)**

For peer review only - http://bmjopen.bmj.com/site/about/guidelines.xhtml

Task allocation and delegation

Task allocation and task delegation were important considerations. Participants had differed in how they rated the importance of medication-related tasks. Amongst medical staff, transfer planning around medication was often allocated to a junior team member and this was seen as a “low-level” task. Whereas critical care pharmacists and ACCPs considered these more complex tasks.

A lot of them [junior doctors] will just write, see [electronic prescription], and that's it. They aren't actually doing a formal review of the meds. […] it's their first job in critical care, and they can just get lumbered to do these discharge summaries because they are a straightforward thing to do […] They maybe don’t go into the complexities that they should do in some of them. **(P14 ACCP)**

Moreover, when it came to medication review on ICU patient transfer, staff groups didn’t always seem aware of the roles and responsibilities of other staff groups.

In terms of when they step down to the ward, I don't know what role our ICU pharmacists have there. I'm not sure I've actually known they've had any active role in that. I think they would flag if they're still on a medication that can't be given or they think their role is much more with new patients and new prescriptions in ICU. I'm sure ward pharmacists, when they go to the ward, …I'm much more imagining it's their role to review the new prescriptions coming to the ward. **(P15 ICU Junior Doctor)**

ICU participants suggested responsibility for medication review lay with the hospital ward team or general practitioner and that ‘*someone else will do it.’* **(P9 ICU Consultant)**

It was suggested that a specific staff group or groups needed to take ownership of the medication review prior to the patients ICU to ward transfer. The ACCP or critical care pharmacist were considered the most appropriate staff groups to do this. Both these staff groups had the benefit of service stability over the frequent rotation of junior medical staff.

Knowledge, Skills and professional diversity

Different staff groups were felt to have different knowledge and skills, for example medical staff diagnostic and treatment planning skills versus pharmacist medication planning and prescribing skills.

I've got to say that the pharmacists and doctors, I would say the pharmacists within critical care are really good at identifying drugs that’s been withheld and putting it in their plan. And then the doctors have got good at looking at the pharmacy plan and pulling it through into the final discharge report. **(P1 Outreach Team)**

For peer review only

For peer review only - http://bmjopen.bmj.com/site/about/guidelines.xhtml

ACCPs were thought to have knowledge and skills in medication reviews through having *'done the process hundreds of times'* **(P5 ICU Consultant)**, in comparison to consultants and junior doctors.

On the hospital ward, there appeared a hesitancy to review medication that had been commenced whilst the patient was in ICU. This seemed to stem from a lack of familiarity with ICU medication and a feeling that the ICU team knew what they were doing. This was compounded by lack of knowledge and skills of junior medical and pharmacy staff meaning that acute medications could continue longer term.

I think it's… to get an effective medication review, I think it depends on the pharmacist and their experience, which has been the key thing. So I think if you've got really junior staff doing a medication review on a patient that's stepped down from ICU, I would imagine they'd find it difficult, […]. And I feel like they would be more reluctant to make medication changes or recommendations to doctors. **(P19 Ward Clinical Pharmacist)**

Theme 2. Time pressures and considerations

Time pressures

Time pressures and considerations affected the system performance (Fig 1). Time pressures contributed to staff not always completing information such as the rationale for prescribing, pausing or de-prescribing medication.

…a doctor changes a medicine or stops a medicine, then when you do it on the [e-prescribing system], you're supposed to fill in a box to say what you've done […] (people are) basically doing this as quite a quick exercise, they'll just go, click, click, click and then they bypass that very often. And we all do it … **(P11 Critical Care Pharmacist)**

Time pressures around the ICU transfer process contributed to some patients being transferred without the opportunity to undertake a prior medication review. Time pressures could mean that staff handovers did not occur and transfer reports and medication plans were not completed.

I think there is often a lot of time pressure on the discharge planning process and that would definitely make a slip type error, because you're so focused on getting the task done, you do what is necessary and then this is probably not absolutely necessary to get the patient discharged and complete your task it's an additional thing isn't it? What you're trying to do is get the patient out onto the ward with everything they need, rather than what else? **(P5 ICU Consultant)**

Workload & staffing levels

For peer review only

One barrier to completing medication reviews was in the healthcare staff-patient ratio particularly on the hospital ward.

It's predominantly the number of patients to see. And also slightly lack of staff on the wards as well. Because if you just have one consultant and one junior, they'll be trying to look things up on the computer, get the meds up, talk to the consultant, see the patient, and then not always have time to then sit and do the prescription at the same time. **(P20 Ward Junior Doctor)**

The benefits of clinical pharmacist medication review were acknowledged to be hampered by reduced dependability borne out of insufficient staffing, lack of 7-day services and set daytime working patterns.

I think the biggest constraint in the system is provision of clinical pharmacy staff. […] actually having the pharmacists time available to undertake those medicines safety checks is the core part of business. And there's only a few of them, […] there's certain things that we as clinicians can do to mitigate that to a degree. […] well the root cause of the problem is […] insufficient pharmacy staff time. **(P9 ICU Consultant)**

Out of hours discharges

Out of hours transfer of patients from ICU to a hospital ward were regarded as particularly high-risk for medication errors.

…. particularly in out of hours, unfortunately, just because of bed availabilities and pressures, that can be quite challenging in terms of information being passed from one team to the next. So information is lost almost about new medications that are commenced for various reasons on the ICU. **(P21 ACCP)**

Discharges out of hours were seen as more problematic because of reduced ICU and hospital ward staffing levels, particularly with the unavailability of wider specialist teams and fewer senior healthcare staff available. It was felt that daytime discharges were conducted better, allowed '*the pharmacists (to) provide a review of the discharge'* **(P5 ICU Consultant)** and increased the likelihood of multiprofessional and senior medical staff review. Sometimes out of hours transfers were unavoidable when ICUs were full and acute admissions to the unit were required for patient flow.

There are a lot of our patients that are discharged out of hours, after 6:00 pm, and that shouldn't be happening. Our patients should be out in a timely manner, sat on a ward, safety, so people have time to make sure everything's documented and sorted for that patient before the nightshift […]. **(P14 ACCP)**

Bed availability and service standards

For peer review only - http://bmjopen.bmj.com/site/about/guidelines.xhtml

Patient flow challenges created pressures on ICU and hospital bed availability, which in turn created issues for patient transfer planning and review. The pressure to get a patient discharged within specified service standards was identified. Transfers could be rushed with short notice of ward bed availability.

…it could be that a patient's on ICU, they've been ready to go to the ward for four or five days, there's not been a bed available for them. And then we've got say three poorly patients out on the wards in A&E that urgently need a bed. So it's sort of a rapid discharge then to clear the beds. **(P7 Outreach Team)**

Theme 3. Communication processes and challenges

Effective communication was seen as a key safety component in transition of patient care '*working together with good communication'* **(P1 Outreach Team)**. Conveyance of accurate and complete medication information (e.g. medication changes) on patient transfer was felt important overall. Several challenges to effective communication were apparent. Firstly, the considerable number of healthcare staff involved in information transfer was seen as a barrier to effective communication. Pertinent information could get lost in the sheer volume of exhaustive patient notes after an ICU care episode, creating an accessibility barrier. Barriers to information accessibility, and in the case of clinical pharmacists meant they reviewed fewer patients. Finally, information was not always communicated, and staff would sometimes forget to include key details. Several systems were employed to improve communication of information including medication, principally handovers, transfer summaries and prescription annotations.

Handovers (ICU to hospital ward)

Staff utilised handover processes to convey information on transitions of patient care. Handovers were verbal, written or combinations thereof (Fig 2). ICU medical, nursing and pharmacy staff had handover processes to their respective hospital ward colleagues. Face-to-face handovers were seen by some as being the best method, whilst transfer of verbal information around shift changes or out of hours were considered higher risk for being lost in the system. Medical, nursing and pharmacy staff had limited awareness of what each other handed over and how this was undertaken. This contributed to assumptions or uncertainties that other staff groups were handing this information over. There was some uncertainty over what information to convey about medication on transitions of care. Medical staff tended to only handover information that they perceived to be important, that needed to be followed up related to the key patient problems e.g., a scan needs to be requested. It

For peer review only - http://bmjopen.bmj.com/site/about/guidelines.xhtml

was often unclear if medication details would be included or not, however, this information was often considered of low interest for medical staff.

I don't think the doctors, [...] have a verbal handover. [...] everything's documented on this transfer medication report and so it's at their discretion for them to read it and to make sure that everything's handed over. [..] But in terms of, like, a verbal handover I know that doesn't happen. ***(P18 Ward Clinical Pharmacist)***

Transfer summary

The transfer summary was considered the communication focus, providing an effective means of highlighting and collating essential information and care plans, and considered more dependable than verbal handovers. When completed well, the transfer summary was used to convey information and prompt medication reviews.

So within that there are fields specifying medication comments and actions, ongoing actions for the ward. So we try to put into that the information, so it doesn't get lost and that's the way to try and improve that safety and that communication around medications. But if they become well to step out and then there's bed pressures and they don't step for a day or two that information can almost get lost within all the notes. **(P21 ACCP)**

Furthermore, having the transfer summary easily accessible electronically by all healthcare professional was deemed important since *'everyone knows it's there [..] the receiving team can read that'.* **(P7 Outreach Team)**

Prescription annotations

Annotations on the electronic prescription was seen as an effective way of maintaining focused, easily accessible information regarding medication. Prescribing important chronic medication and then holding or suspending it on the electronic prescribing system was seen as a prompt for medication review. However, annotated information was often omitted, particularly if transcription was required.

So, I think when it's done properly and it's suspended and... Our [e-prescribing system] is great, you can write all sorts of qualifiers, indicators, you can write all sorts on there as a note for future teams. And I think that's when I think it makes most sense. And if I'm receiving a patient and someone's written a note to me, it's just pretty obvious what I've got to do. That all works well. I think when people just leave them off or stop them, but don't write reasons. When you stop any medication there is a comment to cease it and often that's not filled in either. **(P15 ICU Junior Doctor)**

For peer review only - http://bmjopen.bmj.com/site/about/guidelines.xhtml

Theme 4. Technology and systems

Technology was utilised to prescribe medication, record and store information about patients' medicines, prioritise workload and to facilitate communication with other healthcare professionals. Overall, electronic prescribing was felt to have safety benefits over paper prescription and charts with prescription defaults, order sets, improved decision support and prompts and accessibility to information such as medicines reconciliation. Electronic prescribing also made information retrieval about medication changes easier to undertake, aiding medication review.

Technology also supported the use of medication transfer checklists and had further potential application via automation of some processes such as information pooling and patient prioritisation.

There's also an inbuilt checklist into the critical care system, so when a patient's being discharged, the doctor is supposed to complete all the discharge information. And before they can do that there is a checklist which they are supposed to tick off which says, I have reviewed all the patient's usual medicines, I have reviewed all the current medicines to make sure that nothing's inappropriately being continued,... **(P2 Critical Care Pharmacist)**

However, it was noted that over time checklists became less effective as staff fatigue with the system and find workarounds. Although all ICUs used electronic prescribing, there was some requirement on specific ICUs to use paper prescriptions for some medicines (e.g. intravenous medicines and fluids). One centre had separate ICU and ward-based systems that did not communicate directly, creating an electronic transcription phase to ICU patient transitions. Participants were clear on the importance of full integration of e-prescribing systems, so that all medications were fully visible and auditable in a single format and transcription was not required.

It would say be better if everything was electronic and go to the ward electronic, because things get misfiled, don't they? […] Lost. If it was all electronic and got sent electronically, then that would be the safest way. I think often with prescribing problems that can be a problem, when some things are electronic and some things are paper, then it gets very confusing, doesn't it? **(P12 ACCP)**

System functionality was emphasised in minimising the number of phases or menus required in the e-prescribing system to prescribe, hold, and plan reviews of medication. Limitations in functionality disinclined staff to prescribe medication not immediately required, which compromised the identified communication benefits of prescribing and holding a potentially important long-term medication requiring regular review for restarting when clinically appropriate. The importance of accessibility was identified when it came to finding required information quickly, minimising loss of information within menu caches.

For peer review only - http://bmjopen.bmj.com/site/about/guidelines.xhtml

Over-reliance on the individual to undertake tasks increased the risk of them not being done.

I think, I think a lot of the errors occur because it's down to individuals to do things. And you can put systems in place, and you can put protocols in place, but people will always find a way around them, when you're busy you'll always take the easiest route.[…] Whereas, if you had ways of connecting up systems so that it's automatically done, then it's less reliant on the individuals. The individuals would still need to be involved in it and can still up on errors and add to the system, but you would probably get fewer errors, certainly less, sort of, information loss if the systems talked to each other. **(P2 Critical Care Pharmacist)**

Theme 5. Beliefs about consequences for the patient and organisation

Medication errors and adverse patient outcomes

Medication errors reported included inappropriate continuation of medication started in ICU without an ongoing indication, loss of important medication information (e.g. review plan), and failure to restart potentially important chronic medication. Although participants acknowledged such medication errors were common, the outcomes for patients and the impact upon them was not something that participants seemed to universally appreciate or take into consideration.

I've never really thought about that [potential impact on patients]*. I would hope not. I would hope by the point we get to discharge the wards have reviewed all the medications properly and done a decent* [discharge prescription] *[…]. So, they're generally on medication short term to try and help psychosis, which I would hope gets stopped before they get discharged because it's not something we ask to be on long term, but I don't know if I'm honest. It's not something that we follow up on.* (**P14 ACCP**)

Participants identified that the implications would vary depending on certain factors such as the specific medication error and timescale considered and that there was a *'very wide range of possible outcomes of omitting medicines, unintentionally or intentionally'*. **(P10 Ward Clinical Pharmacist)**

Some participants considered the potential for adverse effects as a longer-term risk and would not be apparent until sometime after the hospital discharge (e.g. unintentional discontinuation of secondary prevention medication post-myocardial infarction or in the case below of continuation of anti-psychotic medication).

We had […] a case about ten years ago where a patient was prescribed three different medicines for delirium, different psychiatric medicines while they were on unit, then discharged to the ward, went home and then eventually they went back to their GP, was asked to sign their repeat prescription three months later and he looked at it and said, I didn't know this patient was under psychiatric care. And

For peer review only - http://bmjopen.bmj.com/site/about/guidelines.xhtml

then looked into it and they'd basically never been stopped, the patient went home on anti-psychotic medicines, nobody stopped him. **(P11 Critical Care Pharmacist)**

Units that had undertaken a review on incidents, clinical audits or operated follow up clinics appeared more cognisant of potential medication issues after ICU patient transfer and implications for patient recovery. Some participants reported unit follow up of clinical incidents related to adverse drug events had been undertaken. Others had undertaken clinical audits or service evaluations of medication continuity and safety, which increased staff awareness of the high rates of medication errors on transition from ICU.

Yeah, 'cause we have had a couple of incidents and we've learned from them, and in fact our pharmacist did an audit on it, and presented that audit to the MDT, and there was a lot of learning points from that, and it has brought to us the importance, 'cause I think people just didn't, it just wasn't very high up in people's concerns about a patient. **(P12 ACCP)**

Feedback from follow up clinics were another way staff learnt about medication errors on transition and patient adverse drug events. These safety culture processes reduced the chance of siloed ICU perspectives on the patient care pathway.

Patient and family engagement

Participants identified that ICU patients frequently experienced delirium and memory loss which was a challenge to potential engagement in medication discussions and information whilst in ICU. Concerns about lack of patient recollection was a barrier to healthcare professional communication around medication handover on planned transfer to the hospital ward. It was unclear what perceived role family members had in this scenario. Whilst in ICU, the care team tended to take over all control of patient medication.

..we take complete control of their medications, nines time out of ten in hospital, so we are, you know, we are basically putting it all in our hands, and responsibility therefore. **(P9 ICU Consultant)**

Patient involvement in their medication through guiding discussions and questioning changes, was highlighted when their condition improved. Consequently, the role of the patient and family in medication safety and continuity whilst in ICU appeared passive, rather than being active agents.

And usually you find that a patient who's got long term illness, they're usually quite good at questioning medication. I can foresee that patients on intensive care, they've got poor concentration, attention, and they'll be coming to that remit of, the nurse [...] gave me six tablets, I'll just take them without asking any questions, et cetera. **(P1 Outreach Team)**

For peer review only - http://bmjopen.bmj.com/site/about/guidelines.xhtml

Discussion

This is the first qualitative study focused on understanding the socio-technical factors of medication safety in patients transitioning from ICU. We identified thirteen factors within five broad themes, providing an interactive description of the factors that most strongly influenced the system performance. By highlighting the important system factors at an ICU and wider organisational levels, we provide a focus for service improvement efforts. Notably, time pressure considerations (workload, staffing, shift work, out of hours transfers, bed availability) have a marked negative effect on the system performance. We now discuss how these factors impacted on team and perceived system performance in relation to the key socio-organisational and work system components identified [29].

Care team factors. We saw diverse multiprofessional staff groups, working in ICU and hospital-ward teams, were agents in the delivery of medication continuity and safety on ICU patient transfer. The performance of care teams was affected by staff knowledge and skills in medication review and prescribing, being perceived to vary across healthcare professional groups. Here, the core stability of some ICU staff groups, benefitted their practice and experience, over staff with regular clinical rotations. Importantly, limitations in understanding of the potential impact of medication errors on patient or organisation outcomes undermined the meaning of mitigating tasks or processes undertaken, particularly amongst rotational staff groups. These limitations in ICU and ward-based staff beliefs about consequences of medication errors on this care interface present a challenge to the success of future behaviour change interventions [43].

We identified that the patient and family were often considered co-agents by ICU staff, reducing their engagement. Barriers to agent status included delirium and memory loss for patients. These findings may explain some of the limited patient awareness of medication changes identified on follow up [18]. Nevertheless, lack of communication and family support may also be factors [2]. Engaging patients and family members around safety of medication in transitions in care is recommended [7], and there are opportunities, such as written communication [44], to support the resilience and adaptability exhibited by patients and family in recovery [2], and care satisfaction [32].

Task factors. The logistical challenges of patient flow on the transition from ICU to the hospital ward are well known [32,45]. We show that medication safety for ICU patients transitioning to a hospital ward is a complex process, involving complex patients, following an uncertain care pathway, involving diverse multiprofessional healthcare staff groups and personnel organised in multiple teams with varying resources. Process complexity was further increased by being time critical and dependent. Task prioritisation was focused on the more acutely ill patients in ICU, or new patients

For peer review only - http://bmjopen.bmj.com/site/about/guidelines.xhtml

on the hospital ward, potentially affected by staff resources and physical environment factors (ICU bed availability and pressures for beds) [46]. We found some medical staff considered the medication elements were a low priority to include in ICU patient transfer and handover plans, as previously reported by others [32]. In contrast, several medication-related elements (pre-admission medicines, active medications on transfer, rationale for medication regime and intravenous infusions) were considered essential information elements by ICU and ward-based Canadian medical staff [47].

Tools and technologies factors. We report that the full integration of e-prescribing systems across ICU and the hospital ward was clearly important in reducing medication transcription errors. Progression from non-integrated to integrated ICU and hospital ward e-prescribing systems, reduced the frequency and clinical severity of medication errors on transition in ICU patient care in an Australian report [48]. Although, it was also apparent that re-prescribing on transfer provided an opportunity to prompt a structured medication review, previously being associated with reduced risk of medication transfer errors [10]. Communication of information was viewed as supported when it was easily accessible and searchable within the numerous and complicated electronic system menus. The structure and content of the medication-related communication was aided by transfer checklists and summary reports system functionality.

Organisational factors. ICU leadership was provided by the ICU consultant and it was apparent from ICU participants that team performance, as exemplified in multiprofessional ward rounds was supported by psychological safety. This is reassuring given that ward round perception on quality of leadership predicts open multiprofessional communication within ICU [49]. Indeed, ICUs with a multiprofessional ward round are associated with a marked reduction in medication transfer errors [10]. However, ICU team performance is also informed by the composition and interaction of the multiprofessional healthcare team, providing diversity of specialism working on a co-ordinated and goal orientated manner [50]. Although we identified clear professional diversity, there were also indications of opportunities to further improve team effectiveness and performance by strengthening other components (dependability, structure and clarity, meaning, impact) [51].

Communication, particularly inter-team (ICU to Ward) communication, was considered a challenge to medication safety and continuity. These staff communication challenges around patient transfer from ICU to a hospital ward are well described elsewhere [32,52,53]. Limitations in written communication content are common, lacking decision-making information such as the rationale for medications that were started or stopped [53]. Lack of communication about medication may contribute to medication errors in ICU survivors on the hospital ward [52], and inadequate handover

For peer review only - http://bmjopen.bmj.com/site/about/guidelines.xhtml

492 can contribute to the distress patients experience on this transition in care interface [52]. We identified several factors that facilitated communication of medication related information. These included multiprofessional handovers, prescription annotations, outreach teams following up patients and plans, and referrals to specialist teams. Previous systematic reviews [31,54], have identified the importance of handover forms and liaison nurses, improving continuity or care but without any evidence of improved patient outcomes. However, it is not enough that a handover occurred, but that the right information is conveyed, acknowledged and actioned. Automation of facets of medication-related information via technological systems improve information transfer and delivery on ICU patient transitions [55].

Time pressures and considerations affected organisational conditions with time constraints having a notable effect on system performance. The important impact of such time considerations as facilitators and barriers to care during discharge from the ICU have been recently summarised [56]. Patients transferred out of hours from ICUs have worse outcomes, [57] and it is conceivable that medication safety issues contribute to this increased risk.

Our results share consistent themes previously identified in ICU transfers and medication or care continuity. Lack of staff knowledge, unclear task allocation and staff collaboration are a barrier to medication safety on transitions in patient care, as reported for medicines reconciliation processes [58]. Variation and lack of clarity of roles around inpatient medication reviews has previously been identified as an additional risk factor for medication errors, as reported for ward-based clinical pharmacists [59]. Patient severity (complexity) of illness, provider experience (knowledge and skills) and resource constraints (workload and staff availability), have previously been identified as themes affecting ICU patient discharge and readmission risk [60].

Strengths and Limitations

Our study had several strengths. Our findings are strengthened by the inclusion of views of UK healthcare professionals from both the ICU (transferring) and the hospital ward (receiving) multicentre teams, involved in the medication review and prescribing of ICU patients. Views represented teaching and district general hospitals, all of which use electronic prescribing systems. Thematic analysis was framed on the SEIPS 3.0 model, [29] which was developed specifically for evaluating the human factors of patient safety during transitions in care. We provide system interface interactions, including indications of modifiability to assist service improvement endeavours.

For peer review only - http://bmjopen.bmj.com/site/about/guidelines.xhtml

We did not include nursing staff as participants as the focus was medication review and prescribing activities, although ACCP and Outreach Teams are primarily from professional nursing backgrounds, making it likely we captured the main themes here as well. One interview of a hospital junior doctor failed to record properly. The potential impact was minimised by use of interviewer field notes (main points summary), and we benefitted from other participant interviews from the same profession. AAA is a critical care pharmacist and there is potential for bias and positionality to influence the interpretation of the results. We are confident that any risk of these were minimised by a non-ICU healthcare professional (BBB) leading the interviews, frequent reflexive evaluation, undertaking of dual coding of all interviews, and using an iterative process to identify the themes and sub-themes based on submersion in the data and continuous matching of extracts to the thematic analysis.

Recommendations for policy change and further research

Hospital-wide integration of e-prescribing systems across ICU and the hospital ward areas is imperative across this interface in patient care. These e-prescribing systems must have the required functionality (including automation when applicable), to support delivery of medication safety, being informed by an understanding of the information and actions required to effectively undertake component tasks [26]. Patient flow systems across the ICU to ward transfer interface should be reviewed and optimised, to identify and implement modifiable elements to maximise multiprofessional team processes and care continuity within existing time constraints. Inter team communication processes around medication-information need to be streamlined, automated (when possible), co-ordinated and accessible, with structured modes of delivery. Task organisation should include delegation to specific staff groups with the knowledge and skills to routinely undertake medication reviews prior to patient transfer to a hospital ward (e.g. critical care pharmacists and ACCPs). However, for clinical pharmacists to be able to take on routine delivery of these additional roles, their availability needs to be dependable and ensure sufficient staff are available 7-days per week. In terms of team organisation, all staff need to understand their responsibilities and those of colleagues in this facet of patient care to improve team performance. Further research is required to confirm the relationship between medication transfer errors and adverse drug events [8], with ICU patient outcomes. Such evidence would inform staff beliefs about consequences, underpinning the education of staff on the clinical importance and how systems can be used to minimise MEs in this interface of care. Furthermore, this education needs to translate into ICU junior medical staff competency assessments to further support improvements in system effectiveness. Further research is required to understand how ICU patients and/ or their family should be more engaged and involved in medication-related information, enabling more participation in their own treatment and recovery.

For peer review only

For peer review only - http://bmjopen.bmj.com/site/about/guidelines.xhtml

Conclusions

We identified crucial factors within five key themes that ICU and ward-healthcare professionals viewed were important in the safety and continuity of medication for ICU patients transitioning to a hospital ward. The complexity of the interactions on the performance and time dependency was clear. We make recommendations for policy change and further research based on improving: knowledge and skills of staff, sufficient critical care staffing, team performance, communication and collaboration, and patient and family engagement.

Acknowledgements

The authors would like to thank all the participants for taking the time to provide their insightful views that enriched the findings of this research. We also express our gratitude to our patient and public colleagues who contributed to our interpretation of the study findings and wider dissemination of the results.

Author Contributions

RSB, DP and DMA were responsible for conception of this qualitative study. MJ undertook the participant interviews. RSB and MJ coded the transcripts and undertook the thematic analysis. RSB, DP and DMA contributed to interpretating the results. RSB, MJ, DP, JKJ, EB, FW and HM developed the representations of the work-system performance interactions. RSB & MJ wrote the manuscript. All authors read and approved the final manuscript. RSB acts as the guarantor for the intellectual integrity of the data.

Ethics Approval

Ethical (The University of Manchester, Ref: 2020-10852-17342) and NHS Health Research Authority (HRA) (IRAS 292456) approval was granted for the study.

Funding

This report is independent research supported by the National Institute for Health and Care Research NIHR300444. BBB and GGG are funded by the National Institute for Health and Care Research XXXXXX (PSTRC-2016-003). The views expressed in this publication are those of the author(s) and not necessarily those of the NHS, the National Institute for Health and Care Research or the Department of Health and Social Care.

Competing interests

The authors declare that they have no competing interests.

For peer review only

Patient consent

Not applicable. No patients were involved in the study.

Provenance and peer review

Not commissioned; externally peer reviewed

Data availability statement

The datasets used and/or analysed during the current study are available from the corresponding author on reasonable request.

Supporting Information

Supplementary file

Table S1. Electronic prescribing system configurations in ICU and hospital ward at each site

Table S2. Behavioural ICU transition medication error case vignettes

Interview Topic Guide

Figure Abbreviations

Figure 1: CT: Care Team factor; TA: Task factor; TT: Tools and Technology factor; O: Organisational factor; PE: Physical Environment factor; EE: External Environment factor.

For peer review only

For peer review only - http://bmjopen.bmj.com/site/about/guidelines.xhtml

References

1 Rousseau AF, Prescott HC, Brett SJ, *et al.* Long-term outcomes after critical illness: recent insights. *Crit Care* 2021;**25**:1–7. doi:10.1186/s13054-021-03535-3

2 Haines KJ, Hibbert E, Leggett N, *et al.* Transitions of Care After Critical Illness-Challenges to Recovery and Adaptive Problem Solving. *Crit Care Med* 2021;**49**:1923–31. doi:10.1097/CCM.0000000000005095

3 Lone NI, Gillies MA, Haddow C, *et al.* Five-year mortality and hospital costs associated with surviving intensive care. *Am J Respir Crit Care Med* 2016;**194**:198–208. doi:10.1164/rccm.201511-2234OC

4 McPeake J, Bateson M, Christie F, *et al.* Hospital re-admission after critical care survival: a systematic review and meta-analysis. *Anaesthesia* 2022;**77**:475–85. doi:10.1111/anae.15644

5 Turnbull AJ, Donaghy E, Salisbury L, *et al.* Polypharmacy and emergency readmission to hospital after critical illness: a population-level cohort study. *Br J Anaesth* 2021;**126**:415–22. doi:10.1016/j.bja.2020.09.035

6 Brown SM, Bose S, Banner-Goodspeed V, *et al.* Approaches to addressing post-intensive care syndrome among intensive care unit survivors A Narrative Review. *Ann Am Thorac Soc* 2019;**16**:947–56. doi:10.1513/AnnalsATS.201812-913FR

7 World Health Organization. Medication Safety in Transitions of Care. 2019. https://www.who.int/publications/i/item/WHO-UHC-SDS-2019.9

8 Bosma LBE, Hunfeld NGM, Quax RAM, *et al.* The effect of a medication reconciliation program in two intensive care units in the Netherlands: a prospective intervention study with a before and after design. *Ann Intensive Care* 2018;**8**:19. doi:10.1186/s13613-018-0361-2

9 Heselmans A, van Krieken J, Cootjans S, *et al.* Medication review by a clinical pharmacist at the transfer point from ICU to ward: a randomized controlled trial. *J Clin Pharm Ther* 2015;**40**:578–83. doi:10.1111/jcpt.12314

10 Tully AP, Hammond DA, Li C, *et al.* Evaluation of medication errors at the transition of care From an ICU to non-ICU location. *Crit Care Med* 2019;**47**:543–9. doi:10.1097/CCM.0000000000003633

11 Sauro KM, Soo A, de Grood C, *et al.* Adverse events after transition from ICU to hospital ward. *Crit Care Med* 2020;**48**:946–53. doi:10.1097/CCM.0000000000004327

12 Campbell AJ, Bloomfield R, Noble DW. An observational study of changes to long-term medication after admission to an intensive care unit. *Anaesthesia* 2006;**61**:1087–92. doi:10.1111/j.1365-2044.2006.04831.x

13 Bourne RS, Ioannides CP, Gillies CS, *et al.* Clinical frailty and polypharmacy in older emergency critical care patients: A single-centre retrospective case series. *Eur J Hosp Pharm* 2021;:ejhpharm-2020-002618. doi:10.1136/ejhpharm-2020-002618

14 Royal Pharmaceutical Society. Polypharmacy: Getting our medicines right. London: 2019. https://www.rpharms.com/recognition/setting-professional-standards/polypharmacy-getting-our-medicines-right

15 Wang Y, Zhang X, Hu X, *et al.* Evaluation of medication risk at the transition of care: a cross-sectional study of patients from the ICU to the non-ICU setting. *BMJ Open* 2022;**12**:e049695. doi:10.1136/bmjopen-2021-049695

For peer review only - http://bmjopen.bmj.com/site/about/guidelines.xhtml

16 Scales DC, Fischer HD, Li P, *et al.* Unintentional Continuation of Medications Intended for Acute Illness After Hospital Discharge: A population-based cohort study. *J Gen Intern Med* 2016;**31**:196–202.

17 Bell CM, Brener SS, Gunraj N, *et al.* Association of ICU or hospital admission with unintentional discontinuation of medications for chronic diseases. *JAMA J Am Med Assoc* 2011;**306**:840–7.

18 MacTavish P, Quasim T, Purdie C, *et al.* Medication-related Problems in Intensive Care Unit Survivors: Learning from a Multicenter Program. *Ann Am Thorac Soc* 2020;**17**:1326–9. doi:10.1513/AnnalsATS.202005-444RL

19 Stollings JL, Bloom SL, Wang L, *et al.* Critical Care Pharmacists and Medication Management in an ICU Recovery Center. *Ann Pharmacother* 2018;**52**:713–23. doi:10.1177/1060028018759343

20 Bosma LBE, van Rein N, Hunfeld NGM, *et al.* Development of a multivariable prediction model for identification of patients at risk for medication transfer errors at ICU discharge. *PLoS One* 2019;**14**:e0215459. doi:10.1371/journal.pone.0215459

21 Fray M, Waterson P, Carman E. Care transitions in healthcare: The 'high-hanging fruit'. *Appl Ergon* 2021;**95**:103437. doi:10.1016/j.apergo.2021.103437

22 Co I, Hyzy RC. Lost in Transition: A call to arms for better transition from ICU to hospital Ward. *Crit Care Med* 2020;**48**:1075–6. doi:10.1097/CCM.0000000000004381

23 Rhodes A, Moreno RP, Azoulay E, *et al.* Prospectively defined indicators to improve the safety and quality of care for critically ill patients: a report from the Task Force on Safety and Quality of the European Society of Intensive Care Medicine (ESICM). *Intensive Care Med* 2012;**38**:598–605.

24 Prescott HC, Angus DC. Enhancing recovery from sepsis: A review. *JAMA - J Am Med Assoc* 2018;**319**:62–75. doi:10.1001/jama.2017.17687

25 Bourne RS, Jennings JK, Panagioti M, *et al.* Medication-related interventions to improve medication safety and patient outcomes on transition from adult intensive care settings: a systematic review and meta-analysis. *BMJ Qual Saf* 2022;**31**:609–22. doi:10.1136/bmjqs-2021-013760

26 Bourne RS, Phipps DL, Jennings JK, *et al.* Medication safety for intensive care patients transferring to a hospital ward: A Hierarchical Task Analysis. *Hum Factors Healthc* 2022;**2**:100030. doi:10.1016/j.hfh.2022.100030

27 Bourne RS, Jennings JK, Ashcroft DM. A Delphi consensus study to identify priorities for improving and measuring medication safety for intensive care patients on transfer to a hospital ward. *Int J Qual Heal Care* 2022;**34**. doi:10.1093/intqhc/mzac082

28 Sevdalis N, Brett SJ. Improving care by understanding the way we work: human factors and behavioural science in the context of intensive care. *Crit Care* 2009;**13**:139. doi:10.1186/cc7787

29 Carayon P, Wooldridge A, Hoonakker P, *et al.* SEIPS 3.0: Human-centered design of the patient journey for patient safety. *Appl Ergon* 2020;**84**:103033. doi:10.1016/j.apergo.2019.103033

30 Donchin Y, Gopher D, Olin M, *et al.* A look into the nature and causes of human errors in the intensive care unit. *Qual Saf Health Care* 2003;**12**:294–300. doi:10.1136/qhc.12.2.143

For peer review only

For peer review only - http://bmjopen.bmj.com/site/about/guidelines.xhtml

31 van Sluisveld N, Hesselink G, van der Hoeven JG, *et al.* Improving clinical handover between intensive care unit and general ward professionals at intensive care unit discharge. *Intensive Care Med* 2015;**41**:589–604. doi:10.1007/s00134-015-3666-8

32 Stelfox HT, Leigh JP, Dodek PM, *et al.* A multi-center prospective cohort study of patient transfers from the intensive care unit to the hospital ward. *Intensive Care Med* 2017;**43**:1485–94. doi:10.1007/s00134-017-4910-1

33 Manias E, Williams A, Liew D. Interventions to reduce medication errors in adult intensive care: a systematic review. *Br J Clin Pharmacol* 2012;**74**:411–23.

34 Bueno WP, Saurin TA, Wachs P, *et al.* Coping with complexity in intensive care units: A systematic literature review of improvement interventions. *Saf Sci* 2019;**118**:814–25. doi:10.1016/j.ssci.2019.06.023

35 McNab D, McKay J, Shorrock S, *et al.* Development and application of systems thinking' principles for quality improvement. *BMJ Open Qual* 2020;**9**:e000714. doi:10.1136/bmjoq-2019-000714

36 Scanlon MC, Karsh BT. Value of human factors to medication and patient safety in the intensive care unit. *Crit Care Med* 2010;**38**:90–6. doi:10.1097/CCM.0b013e3181dd8de2

37 Carayon P, Wetterneck TB, Cartmill R, *et al.* Characterising the complexity of medication safety using a human factors approach: An observational study in two intensive care units. *BMJ Qual Saf* 2014;**23**:56–65. doi:10.1136/bmjqs-2013-001828

38 Taylor-Adams S, Vincent C. Systems analysis of clinical incidents: the London protocol. *Clin Risk* 2004;**10**:211–20. doi:10.1258/1356262042368255

39 Reason J. *Human error*. Cambridge: Cambridge University Press 1990. doi:doi:10.1017/CBO9781139062367

40 Braun V, Clarke V. Using thematic analysis in psychology. *Qual Res Psychol* 2006;**3**:77–101. doi:10.1191/1478088706qp063oa

41 Braun V, Clarke V. To saturate or not to saturate? Questioning data saturation as a useful concept for thematic analysis and sample-size rationales. *Qual Res Sport Exerc Heal* 2021;**13**:201–16. doi:10.1080/2159676X.2019.1704846

42 Tong A, Sainsbury P, Craig J. Consolidated criteria for reporting qualitative research (COREQ): a 32-item checklist for interviews and focus groups. *Int J Qual Heal Care* 2007;**19**:349–57. doi:10.1093/intqhc/mzm042

43 Carey RN, Connell LE, Johnston M, *et al.* Behavior Change Techniques and Their Mechanisms of Action: A Synthesis of Links Described in Published Intervention Literature. *Ann Behav Med* 2019;**53**:693–707. doi:10.1093/abm/kay078

44 Tobiano G, Chaboyer W, Teasdale T, *et al.* Older patient and family discharge medication communication: A mixed-methods study. *J Eval Clin Pract* 2021;**27**:898–906. doi:10.1111/jep.13494

45 Hua M, Wunsch H. The complexities of ICU discharge. *Chest* 2015;**147**:281–2. doi:10.1378/chest.14-2377

46 Batchelor A. Adult Critical Care GIRFT Programme National Specialty Report. 2021. https://www.gettingitrightfirsttime.co.uk/wp-content/uploads/2021/08/Adult-Critical-Care-Aug21L.pdf

For peer review only

For peer review only - http://bmjopen.bmj.com/site/about/guidelines.xhtml

47 de Grood C, Job McIntosh C, Boyd JM, *et al.* Identifying essential elements to include in Intensive Care Unit to hospital ward transfer summaries: A consensus methodology. *J Crit Care* 2019;**49**:27–32. doi:10.1016/j.jcrc.2018.10.001

48 Dabliz R, Poon SK, Fairbrother G, *et al.* Medication safety improvements during care transitions in an Australian intensive care unit following implementation of an electronic medication management system. *Int J Med Inform* 2021;**145**:104325. doi:10.1016/j.ijmedinf.2020.104325

49 Reader TW, Flin R, Mearns K, *et al.* Interdisciplinary communication in the intensive care unit. *Br J Anaesth* 2007;**98**:347–52. doi:10.1093/bja/ael372

50 Ervin JN, Kahn JM, Cohen TR, *et al.* Teamwork in the intensive care unit. *Am Psychol* 2018;**73**:468–77. doi:10.1037/amp0000247

51 Google. Project aristotle. https://rework.withgoogle.com/print/guides/5721312655835136/

52 Field K, Prinjha S, Rowan K. 'One patient amongst many': A qualitative analysis of intensive care unit patients' experiences of transferring to the general ward. *Crit Care* 2008;**12**:21. doi:10.1186/cc6795

53 Brown KN, Leigh JP, Kamran H, *et al.* Transfers from intensive care unit to hospital ward: A multicentre textual analysis of physician progress notes. *Crit Care* 2018;**22**. doi:10.1186/s13054-018-1941-0

54 Wibrandt I, Lippert A. Improving Patient Safety in Handover From Intensive Care Unit to General Ward: A Systematic Review. *J Patient Saf* 2020;**16**:199–210. doi:10.1097/PTS.0000000000000266

55 Parsons Leigh J, Brundin-Mather R, Zjadewicz K, *et al.* Improving transitions in care from intensive care units: Development and pilot testing of an electronic communication tool for healthcare providers. *J Crit Care* 2020;**56**:265–72. doi:10.1016/j.jcrc.2020.01.019

56 Plotnikoff KM, Krewulak KD, Hernández L, *et al.* Patient discharge from intensive care: an updated scoping review to identify tools and practices to inform high-quality care. *Crit Care* 2021;**25**:1–13. doi:10.1186/s13054-021-03857-2

57 Vollam S, Dutton S, Lamb S, *et al.* Out-of-hours discharge from intensive care, in-hospital mortality and intensive care readmission rates: a systematic review and meta-analysis. *Intensive Care Med* 2018;**44**:1115–29. doi:10.1007/s00134-018-5245-2

58 van Sluisveld N, Zegers M, Natsch S, *et al.* Medication reconciliation at hospital admission and discharge: insufficient knowledge, unclear task reallocation and lack of collaboration as major barriers to medication safety. *BMC Health Serv Res* 2012;**12**:170. doi:10.1186/1472-6963-12-170

59 Helathcare Safety Investigation Branch. The role of clinical pharmacy services in helping to identify and reduce high-risk prescribing errors in hospital. 2020. https://www.hsib.org.uk/documents/249/hsib-report-high-risk-prescribing-errors-hospital.pdf

60 Ofoma UR, Dong Y, Gajic O, *et al.* A qualitative exploration of the discharge process and factors predisposing to readmissions to the intensive care unit. *BMC Health Serv Res* 2018;**18**:6. doi:10.1186/s12913-017-2821-z

For peer review only

For peer review only - http://bmjopen.bmj.com/site/about/guidelines.xhtml

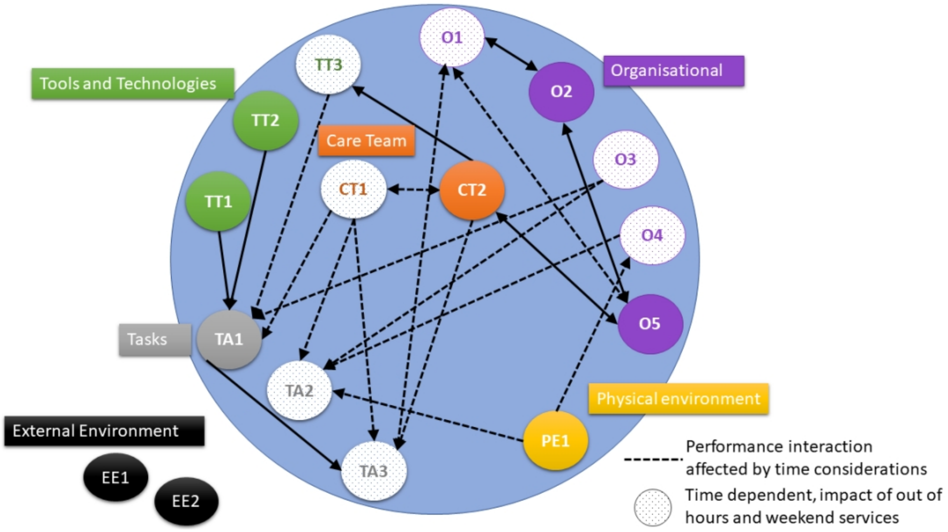

Fig 1: ICU patient transfer performance interaction

338x190mm (96 x 96 DPI)

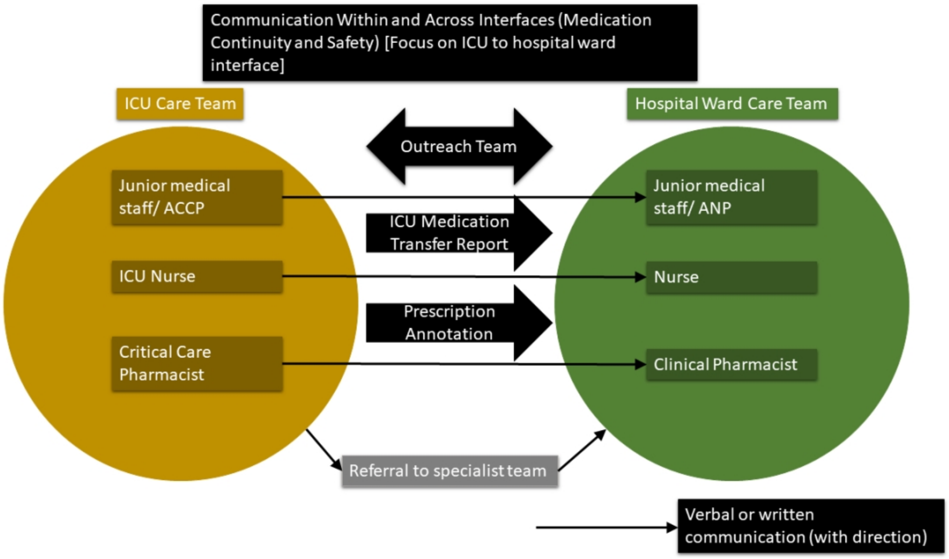

Fig 2: Medication continuity and safety communication across ICU to hospital ward interfaces

338x190mm (96 x 96 DPI)

Supplementary File

The multicentre study was conducted in 4 general intensive care units (ICUs) across 3 Trusts in the north of England (XXX (XXX NHSFT), YYY (YYY NHST), The AAA and BBB Hospital (both ZZZ NHS Trust)).

Centre	Hospital Type	ICU	Ward	Comment
XXX	Teaching	e-prescribing (MetaVision, iMDsoft)	e-prescribing (Lorenzo, DXC Technology)	No inter-system electronic communication. All ward-based infusion prescriptions on paper
YYY	Teaching	e-prescribing (MedChart)	e-prescribing (MedChart)	Inter-system communication. Complex and variable rate infusions are prescribed on paper
AAA	District General	e-prescribing (MedChart)	e-prescribing (MedChart)	Inter-system communication. Complex and variable rate infusions are prescribed on paper
BBB	Teaching	e-prescribing (Allscripts)	e-prescribing (Allscripts)	Inter-system communication. Total parenteral nutrition (TPN) prescribed on paper

Table S1. Electronic prescribing system configurations in ICU and hospital ward at each site

**Case 1 (slip and lapse type mistake)**
Error: Missed prescription for an antihypertensive drug (losartan) on ICU and hospital discharge, with that patient being hypertensive on return to GP practice.
Contextual factors: The losartan had not been prescribed in ICU as the patient had initially been hypotensive. On ICU discharge the patient was normotensive. The ICU was busy and only the medicines the patient was currently receiving whilst on ICU were transferred to the ward electronic prescription system. Losartan was on the ICU pre-admission

For peer review only - http://bmjopen.bmj.com/site/about/guidelines.xhtml

medication record, but this list was not transferred to the ward electronic system and the medicines reconciliation paper report was not reviewed on the ward.
**Case 2 (rules-based mistake)**
Error: ICU patient with Type 1 diabetes had their blood glucose controlled by a variable-rate insulin infusion. Their long-acting basal insulin was not prescribed whilst they were on a high-dose metaraminol infusion (as per ICU guidelines). The metaraminol was weaned off and the patient transitioned to the ward still without their basal SC insulin.
Contextual factors: Hospital guidelines for all Type 1 diabetic patients on a variable-rate insulin infusion is to continue their usual subcutaneous basal insulin to reduce the risk of diabetic ketoacidosis if the infusion is interrupted. ICU medical and pharmacy staff did not start the basal insulin after the metaraminol infusion was discontinued and this error was not identified prior to the ward transition. Only active medicines were transferred to the ward prescribing system. The Diabetes Specialist Team identified the error when they reviewed the patient on the ward.
**Case 3 (knowledge-based mistake)**
Error: An ICU patient receiving TPN was discharged to a ward without TPN prescribed on the paper fluid chart and didn't receive any TPN over the weekend (2 days).
Contextual factors: The trainee medical staff didn't know they needed to transfer the electronic TPN prescription from the ICU system to the paper fluid chart for the ward. The discharge was at the weekend and wasn't checked by a clinical pharmacist. Ward staff did not identify the patient was on TPN until the Nutrition Support Team reviewed the patient on Monday.

Table S2. Behavioural ICU transition medication error case vignettes

For peer review only

Interview Topic Guide

Interview questions:

Designation

1. What is your specific role at the hospital?

- How long have you been in this post?

Prior understanding of ICU patient transfer medication errors and local systems

2. Can you tell me about any prior awareness/knowledge/experience you have of any ICU patient transfer medication errors?

- If so, were these due to an error with medication review, or prescribing or both?
- In your opinion, do such errors significantly impact on patient care or not?
- Is that in the hospital or the community or both?
  (Explore if this is primarily an ICU, or hospital word, or GP issue or does it traverse all of these areas?)
  How are these errors usually detected?

3. Can you describe any procedures or safety systems are in place in your hospital to reduce medication errors on the interface of ICU patient transfers to the ward?
   (Is there an ICU discharge procedure? If so, please tell me about that discharge procedure? Do outreach services routinely review all patient's medicines on the ward?

4. How well are these procedures and safety systems implemented?

Who is responsible for the delivering the various safety systems mentioned?

Is there clear task allocation by profession or group?

How well are they adhered to in practice?

For peer review only

Case vignette discussions

- Case 1 (slip and lapse type mistake). Error and Contextual factors

So Case 1 is an error in which somebody forgets to do something they should or accidentally do something they shouldn't have done

5. Is this type of error common in your opinion?

How common? *[Rare to very common etc?]*

What do you think contributes most to slips and lapse type errors?

- Can signpost – London Protocol contributory influencing factors e.g. communication, workload, knowledge or skills if asked)

6. What things would help ro reduce errors like these?

   Then, if need be, prompt the participant to talk specifically about procedural/system/staff role contributions as required.

- Case 2 (rules-based mistake). Error and Contextual factors

So Case 2 is an error in which somebody incorrectly applies or doesn't apply a rule to a situation

What do you think contributes most to rule-based errors?

- Can signpost – London Protocol contributory influencing factors e.g. communication, workload, knowledge or skills if asked)

7. Is this type of error common in your opinion?
8. How common? *[Rare to very common etc?]*
9. What things would help to reduce errors like these?"

   Then, if need be, prompt the participant to talk specifically about procedural/system/staff role contributions as required.

- Case 3 (knowledge-based mistake). Error and Contextual factors

So case 3 is an error in which somebody misunderstands or misinterprets what is happening

What do you think contributes most to knowledge-based errors?

- Can signpost – London Protocol contributory influencing factors e.g. communication, workload, knowledge or skills if asked)

10. Is this type of error common in your opinion?
11. How common? *[Rare to very common etc?]*
12. What things would help ro reduce errors like these?"

For peer review only - http://bmjopen.bmj.com/site/about/guidelines.xhtml

Then, if need be, prompt the participant to talk specifically about procedural/system/staff role contributions as required.

Which error(s) do you most frequently encounter?

(Thinking about these errors in general; what is your role in mitigating the risk of these happening?

What could your role be?)

Facilitators and barriers to medication review (ICU patients on ward transfer)

Thinking about medication review for ICU patients during transfer to a ward:

How well does this process work?

What helps you to do it effectively?

What problems do you encounter?"

[*You can use the SEIPS framework to probe the participant for specific areas as required*]

Facilitators and barriers to safe prescribing and monitoring (ICU patients on ward transfer)

Thinking about prescribing and monitoring of medicines on the interface of ICU ward transfer:

How well does this process work?

What helps you to do it effectively?

What problems do you encounter?"

[You can use the SEIPS framework to probe the participant for specific areas as required]

Reducing medication review and/or medication errors on the interface of ICU patient transfer to a ward

13. If you could change one thing to improve the safety and continuity of medication on the ICU to ward interface – what would that be?
14. Have you any other comments or observations on the topic of medication continuity or safety in ICU patients on the interface of ward transfer?
15. Thank you for your time and helpful views.

For peer review only - http://bmjopen.bmj.com/site/about/guidelines.xhtml

Factor Types	Contributory influencing factor
Patient factors	Condition (complexity and seriousness)<br>Language and communication<br>Personality and social factors
Task and technology factors	Task design and clarity of structure<br>Availability and use of protocols<br>Availability and accuracy of test results<br>Decision-making aids
Individual (staff) factors	Knowledge and skills<br>Competence<br>Physical and mental health
Team factors	Verbal communication<br>Written communication<br>Supervision and seeking help<br>Team structure (congruence, consistency, leadership etc.)
Work environmental factors	Staffing levels and skills mix<br>Workload and shift patterns factors<br>Design, availability and maintenance of equipment<br>Administrative and managerial support<br>Environment<br>Physical
Organisation & management factors	Financial resources and constraints<br>Organisational structure factors<br>Policy, standards and goals<br>Safety culture and priorities
Institutional context factors	Economic and regulatory context<br>National health service executive<br>Links with external organisations

London Protocol: Framework of contributory factors influencing clinical practice

For peer review only

For peer review only - http://bmjopen.bmj.com/site/about/guidelines.xhtml

**Work System**	Team members
	Tasks
	Technologies and tools
	Organisation
	Physical environment
	External environment
**Process (“Medication Review & prescribing”)**	Information gathering
	Information integration & Interpretation
	Medication plan
	Communication of the plan
	Treatment (Medication received & monitoring)
**Outcomes**	Patient (Adverse events, increased length of hospital stay, readmission to ICU or hospital, death)
	System (Effects on quality, safety, cost, efficiency, morale, public confidence in the health system)
	Learning from medication errors

SEIPS 3.0 Framework

For peer review only

Understanding medication safety involving patient transfer from intensive care to hospital ward: A qualitative socio-technical factors study

COREQ checklist

Note: in order to minimize the length of the manuscript, some of the details on the checklist (marked '*') are not included in the manuscript.

For peer review only

Guide question		*Response*	*Page number in manuscript*
1	Interviewer/facilitator	MJ conducted the interviews.	Methods Page 5
2	Credentials	MJ holds an PhD in Pharmacy Practice	Title page
3	Occupation	MJ: Research Associate in medication safety; RSB is a consultant pharmacist	Title page
4	Experience and training	MJ has extensive previous experience of undertaking qualitative research related to medication optimisation and safety in NHS healthcare environments	Methods Page 5
5	Relationship established	MJ was not known to the participants prior to the study. RB knew some of the researchers through professional networks or as co-workers	*
6	Participant knowledge of the research	Participants were made aware of the reasons for doing the research via the information which was given to the participant prior to the interview	Methods Page 4
7	Research characteristics	The researchers had identified the study topic based on a systematic review and meta-analysis they had undertaken of medication-related interventions to impact on medication safety for ICU patients transitioning to the hospital ward. The results of the systematic review identified the lack of theory informed interventions. The human factors interviews were part of a series of studies designed to identify and develop a theory informed intervention package suitable for testing in a future randomised controlled trial.	*
8	Methodological orientation and theory	London Protocol; Systems Engineering for Patient Safety (SEIPS) 3.0 Model	Methods Page 4
9	Sampling	We recruited from a purposive sample of healthcare professionals across four NHS centres chosen to represent both District General and Teaching Hospitals in the north of England. The sampling frame was Intensive Care Units (ICU) and ward-based healthcare professionals. These included medical staff, advanced critical care practitioners (ACCP), critical care	Methods Page 4

		pharmacists, ward-based clinical pharmacists and ICU outreach staff.	
10	Method of approach	Potential participants were identified via the Principal Investigators at each NHS hospital. Potential participants were invited to participate by email. Emails were directed by departmental leads or departmental email distribution lists.	Methods Page 4
12	Sample size	22 participants.	Results page 5 and Table 1
13	Non-participation	A small number of possible participants were approached by the PIs but declined to participate. This was predominantly this was for reasons of time, workload or lack of use of the system. Twenty-four participants consented, of which it was not possible to arrange an interview for 2 of these in a timely manner. Twenty-two participants were interviewed.	*
14	Setting of data collection	All interviews were conducted by MJ and took place via video telecommunication software (Microsoft Teams, Microsoft Corporation, USA)	Methods Page 4
15	Presence of non-participants	No non-participants were present	*
16	Description of sample	Twenty-two ICU and ward-based healthcare professionals were interviewed (3 ICU medical staff, 4 ACCP, 4 Critical Care Pharmacists, 4 Outreach Team Members, 3 Ward Medical Staff, 4 Ward Clinical Pharmacists.	Results Page 5 and Table 1
17	Interview guide	The semi-structured interview schedule was informed by London Protocol and Systems Engineering for Patient Safety (SEIPS) 3.0 Model. These facilitated capture the process, contributing factors and context of medication safety at ICU to ward transition. In order to prompt discussion, we created three medication safety error case vignettes each representing a type of error. The vignettes were shared with participants in advance of the interview.	Methods Page 4
18	Repeat interviews	No follow-up interviews were conducted	
19	Audio/visual recording	Interviews took place via video telecommunication software (Microsoft Teams, Microsoft Corporation, USA) with consent from the participant.	Methods Page 4
20	Field notes	None	Strengths and Limitations Page 21
21	Duration	Interviews ranged in duration from 30-67 minutes with a mean length of 49 minutes.	*
22	Data saturation	Data collection continued until saturation was reached and no new themes emerged from the interviews.	*
23	Transcripts returned	No transcripts were returned to participants	*
24	Number of data coders	Coding was undertaken in duplicate by the qualitative researcher (MJ) and Chief Investigator (RSB)	Methods Page 5
25	Description of the coding tree	Coding was informed by London Protocol and SEIPS 3.0 model frameworks. Coding was completed in three rounds with discussions between the coders after each round	Methods Page 5
26	Derivation of themes	We identified 13 factors in five broad themes. The thematic analysis was then developed to provide a detailed description of the ICU-hospital work-system interface	Results Page 5 Results page 16, Table 2,

For peer review only

			Figures 1 &2
27	Software	QSRNvivo 12 software was utilised to manage the data	Methods Page 5
28	Participant checking	No	*
29	Quotations presented	Please see the results section of the manuscript	Results Page 5-16, Table 2, Figures 1 &2
30	Data and findings consistent		
31	Clarity of major themes		
32	Clarity of minor themes		

For peer review only

BMJ Open

Understanding medication safety involving patient transfer from intensive care to hospital ward: A qualitative socio-technical factors study

Journal:	*BMJ Open*
Manuscript ID	bmjopen-2022-066757.R2
Article Type:	Original research
Date Submitted by the Author:	03-Apr-2023
Complete List of Authors:	Bourne, Richard; Sheffield Teaching Hospitals NHS Foundation Trust, Departments of Pharmacy and Critical Care; The University of Manchester, Division of Pharmacy and Optometry, School of Health Sciences<br>Jeffries, Mark; University of Manchester, Division of Pharmacy and Optometry, School of Health Sciences; NIHR Greater Manchester Patient Safety Translational Research Centre<br>Phipps, Denham; The University of Manchester, Division of Pharmacy and Optometry, School of Health Sciences<br>Jennings, Jennifer K; Sheffield Teaching Hospitals NHS Foundation Trust, Departments of Pharmacy and Critical Care<br>Boxall, Emma; Northern Care Alliance NHS Foundation Trust, Salford Royal Hospital, Pharmacy Department<br>Wilson, Franki; Leeds Teaching Hospitals NHS Trust, Pharmacy Department<br>March, Helen; Northern Care Alliance NHS Foundation Trust, Royal Oldham Hospital, Pharmacy Department<br>Ashcroft, Darren; The University of Manchester, Division of Pharmacy and Optometry, School of Health Sciences; NIHR Greater Manchester Patient Safety Translational Research Centre
**Primary Subject Heading**:	Qualitative research
Secondary Subject Heading:	Intensive care, Health services research, Evidence based practice
Keywords:	Adult intensive & critical care < INTENSIVE & CRITICAL CARE, Health & safety < HEALTH SERVICES ADMINISTRATION & MANAGEMENT, Quality in health care < HEALTH SERVICES ADMINISTRATION & MANAGEMENT, QUALITATIVE RESEARCH

SCHOLARONE™
Manuscripts

I, the Submitting Author has the right to grant and does grant on behalf of all authors of the Work (as defined in the below author licence), an exclusive licence and/or a non-exclusive licence for contributions from authors who are: i) UK Crown employees; ii) where BMJ has agreed a CC-BY licence shall apply, and/or iii) in accordance with the terms applicable for US Federal Government officers or employees acting as part of their official duties; on a worldwide, perpetual, irrevocable, royalty-free basis to BMJ Publishing Group Ltd ("BMJ") its licensees and where the relevant Journal is co-owned by BMJ to the co-owners of the Journal, to publish the Work in this journal and any other BMJ products and to exploit all rights, as set out in our licence.

The Submitting Author accepts and understands that any supply made under these terms is made by BMJ to the Submitting Author unless you are acting as an employee on behalf of your employer or a postgraduate student of an affiliated institution which is paying any applicable article publishing charge ("APC") for Open Access articles. Where the Submitting Author wishes to make the Work available on an Open Access basis (and intends to pay the relevant APC), the terms of reuse of such Open Access shall be governed by a Creative Commons licence – details of these licences and which Creative Commons licence will apply to this Work are set out in our licence referred to above.

Other than as permitted in any relevant BMJ Author's Self Archiving Policies, I confirm this Work has not been accepted for publication elsewhere, is not being considered for publication elsewhere and does not duplicate material already published. I confirm all authors consent to publication of this Work and authorise the granting of this licence.

For peer review only

Understanding medication safety involving patient transfer from intensive care to hospital ward: A qualitative socio-technical factors study

Richard S Bourne[1,2,3*], Mark Jeffries[2,3], Denham L Phipps[2], Jennifer K Jennings[1], Emma Boxall[4], Franki Wilson[5], Helen March[6], Darren M Ashcroft[2,3]

[1] Departments of Pharmacy and Critical Care, Sheffield Teaching Hospitals NHS Foundation Trust, Sheffield, UK

[2] Division of Pharmacy and Optometry, School of Health Sciences, Faculty of Biology, Medicine and Health, The University of Manchester, Manchester, UK

[3] National Institute for Health and Care Research (NIHR) Greater Manchester Patient Safety Translational Research Centre (PSTRC), School of Health Sciences, Faculty of Biology, Medicine and Health, The University of Manchester, Manchester, UK

[4] Pharmacy Department, Salford Royal Hospital, Northern Care Alliance NHS Group, Salford, UK

[5] Pharmacy Department, Leeds Teaching Hospitals NHS Trust, Leeds, UK

[6] Pharmacy Department, Royal Oldham Hospital, Northern Care Alliance NHS Group, Oldham, UK

*Corresponding author

E-mail: richard.bourne1@nhs.net

Word count: 7146

Keywords: Critical Care, Medication Errors, Patient Safety, Systems Analysis, Transitions

For peer review only

For peer review only - http://bmjopen.bmj.com/site/about/guidelines.xhtml

Abstract

Objective

To understand the socio-technical factors affecting medication safety when intensive care patients are transferred to a hospital ward. Consideration of these medication safety factors would provide a theoretical basis, on which future interventions can be developed and evaluated to improve patient care.

Design

Qualitative study using semi-structured interviews of intensive care and hospital ward-based healthcare professionals. Transcripts were anonymised prior to thematic analysis using the London Protocol and Systems Engineering in Patient Safety 3.0 model frameworks.

Setting

Four north of England National Health Service hospitals. All hospitals used electronic prescribing in intensive care and hospital ward settings.

Participants

Intensive care and hospital ward healthcare professionals (intensive care medical staff, advanced practitioners, pharmacists and outreach team members; ward-based medical staff and clinical pharmacists).

Results

Twenty-two healthcare professionals were interviewed. We identified thirteen factors within five broad themes, describing the interactions that most strongly influenced the performance of the intensive care to hospital ward system interface. The themes were: Complexity of process performance and interactions; Time pressures and considerations; Communication processes and

For peer review only

challenges; Technology and systems; and Beliefs about consequences for the patient and organisation.

Conclusions

The complexity of the interactions on the system performance and time dependency was clear. We make several recommendations for policy change and further research based on improving: availability of hospital-wide integrated and functional electronic prescribing systems, patient flow systems, sufficient multiprofessional critical care staffing, knowledge and skills of staff, team performance, communication and collaboration, and patient and family engagement.

Strengths and limitations of this study

- Medication safety for intensive care patients transitioning to a hospital ward requires co-ordination of care within and across multiprofessional teams and environments with varying staff and technological resources.
- This is the first qualitative study focused on understanding the socio-technical factors of medication safety in patients transitioning from intensive care to a hospital ward.
- Study strengths include multicentre views of intensive care and hospital ward UK healthcare professionals, representing teaching and district general hospitals, all using electronic prescribing systems.
- Thematic analysis using the Systems Engineering Initiative for Patient Safety (SEIPS 3.0) model enabled evaluation of the socio-technical factors impacting on medication safety during transition from intensive care to a hospital ward.
- We report on findings from four trusts from England, and findings cannot be generalised to other settings outside the UK.

For peer review only - http://bmjopen.bmj.com/site/about/guidelines.xhtml

Background

Patients recovering from a critical illness requiring intensive care unit (ICU) treatment are generally transferred to a hospital ward before eventual hospital discharge to the community. However, this simplified summary underrepresents the complexity of the patient pathway, where full patient recovery may be protracted, traversing hospital and community care and involve several care transitions [1,2]. Compared to patients discharged from standard hospital care, ICU patients have worse short and medium-term outcomes, requiring additional ongoing healthcare resources [3]. Half of ICU patients require emergency hospital readmission within a year of hospital discharge [4]. This hospital readmission risk is predicted by pre-existing patient multimorbidity, frailty and polypharmacy [4,5]. There is a growing appreciation of the need to improve the recovery experience of ICU survivors, including reducing complications related to polypharmacy and care fragmentation [1,6]. Transitions in care are known to increase patient risks, including those related to medicines usage and care continuity [7]. The transition from ICU to a hospital ward is a marked interface in patient care, with contrasting resources in care team and technological support available in the respective clinical areas. Transition from ICU exposes patients to high rates of medication errors (MEs) [8–10], and adverse events are common, including those that are medication-related [11]. Extensive and frequent medication changes are made during a patient's ICU episode [12,13], increasing the risks for hazardous polypharmacy on care transitions [14]. The most common MEs centre around failure to discontinue inappropriate acute medication without an ongoing indication and not restarting clinically important long-term medication when safe to do so [8–10,15]. Such MEs may remain uncorrected beyond the acute hospital care episode [16–19], and be potentiated by pre-existing polypharmacy [20]. ICU care may therefore compound the known complexities and challenges for patient medication care created by care transitions [7,21].

[22] Organisational recommendations to support the continuity of care, including mitigation of these increased ME risks, have centred around structured staff handovers between ICU and the hospital

For peer review only - http://bmjopen.bmj.com/site/about/guidelines.xhtml

ward to support the continuity of care including a focus on medication [23]. Broader indicators of interventions that can improve medication safety on transitions in patient care are provided by the World Health Organization (WHO) and ICU expert views [7,24]. To date interventions to improve the safety and continuity of medication for ICU patients transitioning to a hospital ward have focused on education of staff, medication review, guidelines, electronic transfer/ handover tool or letter and medicines reconciliation [25]. Simple interventions, based on staff education and guidelines, have been demonstrated to reduce the risk of inappropriate continuation of acute medication no longer indicated [25]. The more complex, and higher clinic risk, of failure to restart clinically important long-term medication, will require a more complex intervention package [25–27].

For any intervention package to be effective in the ICU environment and improve patient care, it is important to understand how the ICU system works [28]. Furthermore, the care interfaces need to be examined in terms of their systems, processes and outcomes [29]. Error prevention in ICU care is dependent on effective inter and intra-multiprofessional team working and communication [30], that are particularly important on patient transition from ICU to the hospital ward [31,32]. Despite ICUs being technology rich environments, an over-reliance on technology to mitigate against medication errors underestimates the complexity of the systems and processes [33,34]. Like other parts of healthcare, ICU and its care transitions involve a complex interplay between technology, tasks and people [35]. Therefore, the importance of human factors engineering has been promoted to address the whole ICU medication management and patient safety pathway [30,36,37].

This study aimed to understand the socio-technical factors affecting medication safety when ICU patients are transferred to a hospital ward. Consideration of these medication safety factors would provide a theoretical basis, on which future interventions can be developed and evaluated to improve patient recovery.

For peer review only

For peer review only - http://bmjopen.bmj.com/site/about/guidelines.xhtml

Methods

Our study used a qualitative design, intended to solicit adult, general ICU and ward-based healthcare professionals directly involved in the prescribing and medication review of ICU patients on the interface of transition to the hospital ward. We recruited from a purposive sample of healthcare professionals across four NHS centres, chosen to represent both District General and Teaching Hospitals in the north of England with a representative range and combination of ICU and hospital ward e-prescribing systems (Supplementary File, Table S1). The sampling frame was ICU (medical staff, advanced critical care practitioners (ACCPs), critical care pharmacists and ICU outreach team members) and ward-based (medical staff, clinical pharmacists) healthcare professionals. Principal Investigators at each NHS hospital directed participant email invitations via departmental team leads or departmental email distribution lists. All participants provided informed written consent.

We undertook semi-structured interviews facilitated by a topic guide adapted from the London Protocol [38] and Systems Engineering Initiative for Patient Safety (SEIPS) 3.0 model [29] (Supplementary File, Topic guide). The London Protocol was developed from Reason's "Swiss Cheese" model, capturing the contributing factors and context around a specific patient safety incident [38]. The SEIPS models are based on the System-Process-Outcome description of approaches to measuring and improving the quality of health care. Version 3.0 of the SEIPS framework includes an expanded process component, focusing on the patient pathway to describe patient interactions with multiple care settings over time [29]. To prompt discussion, we selected three reported ICU medication error cases as vignettes, each representing a type of human error from Reason's (1990) generic error modelling system [39] (see Supplementary File, Table S2). The vignettes were shared with participants in advance of the scheduled interview. All interviews were undertaken via video telecommunication software (Microsoft Teams, Microsoft Corporation, USA), recorded and transcribed verbatim. A qualitative researcher (BBB) experienced in investigation of medicines optimisation practices in NHS healthcare environments conducted the interviews. Transcripts were anonymised prior to deductive thematic analysis [40], using the London Protocol

For peer review only - http://bmjopen.bmj.com/site/about/guidelines.xhtml

[38], and SEIPS 3.0 model [29] frameworks. Coding was supported through qualitative research software (NVivo, Version 12, QSR International (UK) Limited) and undertaken in duplicate by the qualitative researcher (BBB) and Chief Investigator (AAA). All transcript content was coded using the thematic analysis. The coding was completed in three rounds with discussions between the coders after each round, and with the wider research team at different intervals. Data-saturation was considered to be achieved when we judged further interpretation of codes and themes would not provide further insights [41]. Finally, through multiple discussions amongst the research team, we coalesced the main identified factors until they provided a table and parsimonious account of the interactions that most strongly influence the performance of the ICU to hospital ward work system and communication pathways.

We have actively engaged with patient and public involvement (PPI) in this wider research intervention development programme, to improve medication safety for intensive care patients transitioning to a hospital ward. In this study, PPI colleagues consulted on and contributed to the development of the topic guide, interpretation of the thematic findings and dissemination of results.

Ethics

Ethical (The University of Manchester, Ref: 2020-10852-17342) and NHS Health Research Authority (HRA) (IRAS 292456) approval was granted for the study. We have adhered to the Consolidated Criteria for Reporting Qualitative Studies (COREQ) in reporting our findings [42].

For peer review only - http://bmjopen.bmj.com/site/about/guidelines.xhtml

Results

We recruited 22 participants representing each professional group and clinical location (see Table 1 for more details) [41].

Healthcare professional staff group	ICU Medical Staff	Advanced Critical Care Practitioners (ACCP)	Critical Care Pharmacists	Outreach Team Members	Ward Medical Staff	Ward Clinical Pharmacists
**n**	3*	4	4	4	3*	4
**Time in current role (years, mean (SD))**	2.7 (2.1)	6 (2.2)	11 (12.8)	12.3 (5.5)	2 (1)	1.5 (1.3)

Table 1: Healthcare professional participants (n=22)

* Participants from sites 1,3 and 4 only

We identified thirteen factors within five broad themes, providing a detailed description of the ICU-hospital work-system interface. This identified the complexity and interactions within the system and accounted for the significant impact of time pressures and considerations (Table 2, Fig 1). The vulnerability of the system to time constraints was evident (Fig 1). The communication pathways utilised are presented (Fig 2).

ICU-Hospital Ward Work System Interface
ICU Active Agents: ICU Consultant, ICU Junior medical staff, Advanced Critical Care Practitioner, Critical Care Pharmacist, ICU Nurse, Outreach Team, Microbiologist<br>*Hospital Ward Active Agents*: Junior medical staff, Specialist Teams (e.g. Diabetes team, Acute Pain Team), Clinical Pharmacist, Nurse, Outreach Team
**Performance is shaped most *strongly* by interactions of:**
Care Team Factor(s)<br>-CT1: Knowledge and skills of staff on medication review and safe prescribing for ICU patients on the interface of transition to the hospital ward* [Effect dependent on status]<br>-CT2: Beliefs about consequences, understanding of staff to the frequency and clinical impact of medication errors on patient recovery* [Positive effect]
Task Factor(s)<br>-TA1: Task and patient complexity affected by the number of long-term and acute medicines used in patients with a critical illness with unpredictable recovery and responses to treatment including medication [Negative effect]<br>-TA2: Task prioritisation, often focused on the new patient admission(s) or more unstable patient(s) in ICU, with medication use in ICU transfer patient deemed low priority* [Negative effect]<br>-TA3: Task allocation and delegation amongst the care team* [Effect dependent on status]
Tools and Technologies Factor(s)<br>-TT1: E-Prescribing system integration and functionality** [Effect dependent on status]<br>-TT2: Electronic patient notes accessibility and searchability** [Effect dependent on status]<br>-TT3: Medication transfer checklist completion and adherence* [Effect dependent on status]
Organisational Factor(s)

For peer review only - http://bmjopen.bmj.com/site/about/guidelines.xhtml

-O1: Team performance (organisation, leadership, collaboration and dependability)* [Effect dependent on status]<br>-O2: Multiprofessional ward rounds including a medication review element* [Positive effect]<br>-O3: Communication between ICU and Hospital Ward team members*/** [Effect dependent on status]<br>-O4: Time pressures in the need to complete the ICU patient transfer to the ward, staff workload and out of hours transfers*/** [Negative effect]<br>-O5: Safety culture in ICU informed by incident reviews, audit and feedback and follow up clinic experiences* [Positive effect]
Physical Environment Factor(s)<br>-PE1: ICU bed availability and pressure for beds** [Effect dependent on status]
External Environment Factor(s)<br>-EE1: Bed availability on the hospital ward and transfer times** [Effect dependent on status]<br>-EE2: Transfer time standards [Negative effect]
On ICU, Patient and Family co-agents*, also Dietitian, Nutrition support team, Visiting teams and Pharmacy Technician. On the Hospital Ward, the Rehabilitation Team were co-agents.

Table 2: ICU-Hospital Ward Work System Interface

*Potentially modifiable at a local ICU level; ** potentially modifiable at an organisational level

Fig 1: ICU patient transfer performance interaction

Fig 2: Medication continuity and safety communication across ICU to hospital ward interfaces

For peer review only

For peer review only - http://bmjopen.bmj.com/site/about/guidelines.xhtml

Theme 1. Complexity of process performance and interactions

Care Teams and Collaboration

A professionally diverse range of ICU and hospital ward-based healthcare staff undertook important tasks and processes to support medication safety for ICU patients transferring to a hospital ward (Table 2). In the ICU, participants emphasised the value of collaborative, multiprofessional team working that was driven by effective team leadership. ICU consultants led multiprofessional ward rounds, provided leadership and examples for more junior staff in the routine delivery of patient medication reviews. ACCP, medical and nursing staff had important roles in medication review. The role of the critical care pharmacist in medicines reconciliation on admission and undertaking medication reviews for patients, including during ward rounds and prior to hospital discharge was highlighted.

the MDT ward round is really useful, as the [..] consultant you're trying to pull a broad picture of everything together for discharge, and medications is one part of that, and I think we're very lucky in (name of hospital), the quality and the provision of our pharmacy support is excellent. [..] I don't see how we could do it without the pharmacists. **(P5 ICU Consultant)**

On the hospital ward, there was also evidence of multiprofessional team working in undertaking medication reviews in ICU patients transferred to the hospital ward. In some cases, these were facilitated by consultant-led ward rounds. Whilst direct collaboration between the ICU and hospital ward team appeared less clear, the ICU outreach team did provide an important link between the two clinical areas.

So I follow patients up post-discharge from intensive care [...] to look at them physiologically, psychologically, and just follow the plan that's been put in place on a critical care area. [...] Regarding medication, if there's something specific in the discharge plan, then I would review that aspect. **(P1 Outreach Team)**

On the hospital ward, referral to clinical pharmacists and specialist teams in undertaking medication reviews were highlighted. The diabetes team were seen as essential for patients receiving insulin therapy when transferred to a hospital ward and the acute pain team were often engaged with patients with ongoing pain issues.

But there's also other specialists that we get involved [...] we should automatically make a diabetic team referral. So, it's also pulling in other specialist teams into the mix [...] **(P3 ACCP)**

Tasks, processes and prioritisation

For peer review only - http://bmjopen.bmj.com/site/about/guidelines.xhtml

The key medication-related tasks undertaken by ICU healthcare professionals interviewed were medication review, prescribing and plan setting. These were supported by processes such as medicines reconciliation and, in some units, a transfer checklist that included medication elements. ICU transfer reports sometimes included medication information such as pre-admission medicines, current medication and medication plans. Transcribing and transcription checks were also undertaken in ICUs without integrated ICU and hospital ward electronic prescribing systems, which also provided a medication review opportunity.

I would say [the transcription process is] vitally important. So, part of the problem is […] it shouldn't really be seen as a transcription process, it should be more of a review process. […] it's not simply passing straight from one system to another including all that information, it's actually reviewing what's appropriate. **(P2 Critical Care Pharmacist)**

The complexity of tasks undertaken was qualified within the context of ICU patient care and regarded as impacting on medication safety throughout the patient journey. The acuity of the patient condition, and increasing number of medicines they received, contributed to medication review complexity.

we're now seeing patients who are more complex than ever before and on more medicines than ever before, and then, obviously, that becomes even more complex in itself. **(P9 ICU Consultant)**

Tasks were often prioritised, which sometimes meant that the medication-related tasks were seen as a low or lower priority and '*probably the least important thing you would do that day.'* **(P13 Ward Junior Doctor)**. The seriousness of the patient's condition determined the time spent on their care; more seriously ill patients were prioritised over the patient transferring from ICU.

And because it's quite a fast-paced unit those patients could be gone before I see them again because then I could be reviewing the critical unwell patients, the Level 3 patients, the ones that are intubated, and things. So, those patients that are then getting discharged are in the back of my mind because I'm dealing with the sicker ones. **(P14 ACCP)**

The priority for ward-based clinical pharmacists was often focused on undertaking medicines reconciliation for new hospital admissions and discharge prescriptions, over reviewing patients transferred from ICU. Whilst ward medical staff focused on critical medicines.

So I think when you're on the ward round and you go to review the medicines, you mainly look at things that are critical to the patient at the time first. So antibiotics, blood thinners and that sort of thing. And looking back to admission meds to see what's missing while they're still in hospital is not always top of your list. **(P20 Ward Junior Doctor)**

For peer review only - http://bmjopen.bmj.com/site/about/guidelines.xhtml

Task allocation and delegation

Task allocation and task delegation were important considerations. Participants had differed in how they rated the importance of medication-related tasks. Amongst medical staff, transfer planning around medication was often allocated to a junior team member and this was seen as a “low-level” task. Whereas critical care pharmacists and ACCPs considered these more complex tasks.

A lot of them [junior doctors] will just write, see [electronic prescription], and that's it. They aren't actually doing a formal review of the meds. […] it's their first job in critical care, and they can just get lumbered to do these discharge summaries because they are a straightforward thing to do […] They maybe don’t go into the complexities that they should do in some of them. **(P14 ACCP)**

Moreover, when it came to medication review on ICU patient transfer, staff groups didn’t always seem aware of the roles and responsibilities of other staff groups.

In terms of when they step down to the ward, I don't know what role our ICU pharmacists have there. I'm not sure I've actually known they've had any active role in that. I think they would flag if they're still on a medication that can't be given or they think their role is much more with new patients and new prescriptions in ICU. I'm sure ward pharmacists, when they go to the ward, …I'm much more imagining it's their role to review the new prescriptions coming to the ward. **(P15 ICU Junior Doctor)**

ICU participants suggested responsibility for medication review lay with the hospital ward team or general practitioner and that ‘*someone else will do it.’* **(P9 ICU Consultant)**

It was suggested that a specific staff group or groups needed to take ownership of the medication review prior to the patients ICU to ward transfer. The ACCP or critical care pharmacist were considered the most appropriate staff groups to do this. Both these staff groups had the benefit of service stability over the frequent rotation of junior medical staff.

Knowledge, Skills and professional diversity

Different staff groups were felt to have different knowledge and skills, for example medical staff diagnostic and treatment planning skills versus pharmacist medication planning and prescribing skills.

I've got to say that the pharmacists and doctors, I would say the pharmacists within critical care are really good at identifying drugs that’s been withheld and putting it in their plan. And then the doctors have got good at looking at the pharmacy plan and pulling it through into the final discharge report. **(P1 Outreach Team)**

For peer review only - http://bmjopen.bmj.com/site/about/guidelines.xhtml

ACCPs were thought to have knowledge and skills in medication reviews through having *'done the process hundreds of times'* **(P5 ICU Consultant)**, in comparison to consultants and junior doctors.

On the hospital ward, there appeared a hesitancy to review medication that had been commenced whilst the patient was in ICU. This seemed to stem from a lack of familiarity with ICU medication and a feeling that the ICU team knew what they were doing. This was compounded by lack of knowledge and skills of junior medical and pharmacy staff meaning that acute medications could continue longer term.

I think it's… to get an effective medication review, I think it depends on the pharmacist and their experience, which has been the key thing. So I think if you've got really junior staff doing a medication review on a patient that's stepped down from ICU, I would imagine they'd find it difficult, […]. And I feel like they would be more reluctant to make medication changes or recommendations to doctors. **(P19 Ward Clinical Pharmacist)**

Theme 2. Time pressures and considerations

Time pressures

Time pressures and considerations affected the system performance (Fig 1). Time pressures contributed to staff not always completing information such as the rationale for prescribing, pausing or de-prescribing medication.

…a doctor changes a medicine or stops a medicine, then when you do it on the [e-prescribing system], you're supposed to fill in a box to say what you've done […] (people are) basically doing this as quite a quick exercise, they'll just go, click, click, click and then they bypass that very often. And we all do it … **(P11 Critical Care Pharmacist)**

Time pressures around the ICU transfer process contributed to some patients being transferred without the opportunity to undertake a prior medication review. Time pressures could mean that staff handovers did not occur and transfer reports and medication plans were not completed.

I think there is often a lot of time pressure on the discharge planning process and that would definitely make a slip type error, because you're so focused on getting the task done, you do what is necessary and then this is probably not absolutely necessary to get the patient discharged and complete your task it's an additional thing isn't it? What you're trying to do is get the patient out onto the ward with everything they need, rather than what else? **(P5 ICU Consultant)**

Workload & staffing levels

For peer review only - http://bmjopen.bmj.com/site/about/guidelines.xhtml

One barrier to completing medication reviews was in the healthcare staff-patient ratio particularly on the hospital ward.

It's predominantly the number of patients to see. And also slightly lack of staff on the wards as well. Because if you just have one consultant and one junior, they'll be trying to look things up on the computer, get the meds up, talk to the consultant, see the patient, and then not always have time to then sit and do the prescription at the same time. **(P20 Ward Junior Doctor)**

The benefits of clinical pharmacist medication review were acknowledged to be hampered by reduced dependability borne out of insufficient staffing, lack of 7-day services and set daytime working patterns.

I think the biggest constraint in the system is provision of clinical pharmacy staff. […] actually having the pharmacists time available to undertake those medicines safety checks is the core part of business. And there's only a few of them, […] there's certain things that we as clinicians can do to mitigate that to a degree. […] well the root cause of the problem is […] insufficient pharmacy staff time. **(P9 ICU Consultant)**

Out of hours discharges

Out of hours transfer of patients from ICU to a hospital ward were regarded as particularly high-risk for medication errors.

…. particularly in out of hours, unfortunately, just because of bed availabilities and pressures, that can be quite challenging in terms of information being passed from one team to the next. So information is lost almost about new medications that are commenced for various reasons on the ICU. **(P21 ACCP)**

Discharges out of hours were seen as more problematic because of reduced ICU and hospital ward staffing levels, particularly with the unavailability of wider specialist teams and fewer senior healthcare staff available. It was felt that daytime discharges were conducted better, allowed '*the pharmacists (to) provide a review of the discharge*' **(P5 ICU Consultant)** and increased the likelihood of multiprofessional and senior medical staff review. Sometimes out of hours transfers were unavoidable when ICUs were full and acute admissions to the unit were required for patient flow.

There are a lot of our patients that are discharged out of hours, after 6:00 pm, and that shouldn't be happening. Our patients should be out in a timely manner, sat on a ward, safety, so people have time to make sure everything's documented and sorted for that patient before the nightshift […]. **(P14 ACCP)**

Bed availability and service standards

For peer review only - http://bmjopen.bmj.com/site/about/guidelines.xhtml

Patient flow challenges created pressures on ICU and hospital bed availability, which in turn created issues for patient transfer planning and review. The pressure to get a patient discharged within specified service standards was identified. Transfers could be rushed with short notice of ward bed availability.

…it could be that a patient's on ICU, they've been ready to go to the ward for four or five days, there's not been a bed available for them. And then we've got say three poorly patients out on the wards in A&E that urgently need a bed. So it's sort of a rapid discharge then to clear the beds. **(P7 Outreach Team)**

Theme 3. Communication processes and challenges

Effective communication was seen as a key safety component in transition of patient care '*working together with good communication'* **(P1 Outreach Team)**. Conveyance of accurate and complete medication information (e.g. medication changes) on patient transfer was felt important overall. Several challenges to effective communication were apparent. Firstly, the considerable number of healthcare staff involved in information transfer was seen as a barrier to effective communication. Pertinent information could get lost in the sheer volume of exhaustive patient notes after an ICU care episode, creating an accessibility barrier. Barriers to information accessibility, and in the case of clinical pharmacists meant they reviewed fewer patients. Finally, information was not always communicated, and staff would sometimes forget to include key details. Several systems were employed to improve communication of information including medication, principally handovers, transfer summaries and prescription annotations.

Handovers (ICU to hospital ward)

Staff utilised handover processes to convey information on transitions of patient care. Handovers were verbal, written or combinations thereof (Fig 2). ICU medical, nursing and pharmacy staff had handover processes to their respective hospital ward colleagues. Face-to-face handovers were seen by some as being the best method, whilst transfer of verbal information around shift changes or out of hours were considered higher risk for being lost in the system. Medical, nursing and pharmacy staff had limited awareness of what each other handed over and how this was undertaken. This contributed to assumptions or uncertainties that other staff groups were handing this information over. There was some uncertainty over what information to convey about medication on transitions of care. Medical staff tended to only handover information that they perceived to be important, that needed to be followed up related to the key patient problems e.g., a scan needs to be requested. It

For peer review only

For peer review only - http://bmjopen.bmj.com/site/about/guidelines.xhtml

was often unclear if medication details would be included or not, however, this information was often considered of low interest for medical staff.

I don't think the doctors, […] have a verbal handover. […] everything's documented on this transfer medication report and so it's at their discretion for them to read it and to make sure that everything's handed over. [..] But in terms of, like, a verbal handover I know that doesn't happen. ***(P18 Ward Clinical Pharmacist)***

Transfer summary

The transfer summary was considered the communication focus, providing an effective means of highlighting and collating essential information and care plans, and considered more dependable than verbal handovers. When completed well, the transfer summary was used to convey information and prompt medication reviews.

So within that there are fields specifying medication comments and actions, ongoing actions for the ward. So we try to put into that the information, so it doesn't get lost and that's the way to try and improve that safety and that communication around medications. But if they become well to step out and then there's bed pressures and they don't step for a day or two that information can almost get lost within all the notes. **(P21 ACCP)**

Furthermore, having the transfer summary easily accessible electronically by all healthcare professional was deemed important since *'everyone knows it's there [..] the receiving team can read that'.* **(P7 Outreach Team)**

Prescription annotations

Annotations on the electronic prescription was seen as an effective way of maintaining focused, easily accessible information regarding medication. Prescribing important chronic medication and then holding or suspending it on the electronic prescribing system was seen as a prompt for medication review. However, annotated information was often omitted, particularly if transcription was required.

So, I think when it's done properly and it's suspended and… Our [e-prescribing system] is great, you can write all sorts of qualifiers, indicators, you can write all sorts on there as a note for future teams. And I think that's when I think it makes most sense. And if I'm receiving a patient and someone's written a note to me, it's just pretty obvious what I've got to do. That all works well. I think when people just leave them off or stop them, but don't write reasons. When you stop any medication there is a comment to cease it and often that's not filled in either. **(P15 ICU Junior Doctor)**

For peer review only - http://bmjopen.bmj.com/site/about/guidelines.xhtml

Theme 4. Technology and systems

Technology was utilised to prescribe medication, record and store information about patients' medicines, prioritise workload and to facilitate communication with other healthcare professionals. Overall, electronic prescribing was felt to have safety benefits over paper prescription and charts with prescription defaults, order sets, improved decision support and prompts and accessibility to information such as medicines reconciliation. Electronic prescribing also made information retrieval about medication changes easier to undertake, aiding medication review.

Technology also supported the use of medication transfer checklists and had further potential application via automation of some processes such as information pooling and patient prioritisation.

There's also an inbuilt checklist into the critical care system, so when a patient's being discharged, the doctor is supposed to complete all the discharge information. And before they can do that there is a checklist which they are supposed to tick off which says, I have reviewed all the patient's usual medicines, I have reviewed all the current medicines to make sure that nothing's inappropriately being continued,... **(P2 Critical Care Pharmacist)**

However, it was noted that over time checklists became less effective as staff fatigue with the system and find workarounds. Although all ICUs used electronic prescribing, there was some requirement on specific ICUs to use paper prescriptions for some medicines (e.g. intravenous medicines and fluids). One centre had separate ICU and ward-based systems that did not communicate directly, creating an electronic transcription phase to ICU patient transitions. Participants were clear on the importance of full integration of e-prescribing systems, so that all medications were fully visible and auditable in a single format and transcription was not required.

It would say be better if everything was electronic and go to the ward electronic, because things get misfiled, don't they? [...] Lost. If it was all electronic and got sent electronically, then that would be the safest way. I think often with prescribing problems that can be a problem, when some things are electronic and some things are paper, then it gets very confusing, doesn't it? **(P12 ACCP)**

System functionality was emphasised in minimising the number of phases or menus required in the e-prescribing system to prescribe, hold, and plan reviews of medication. Limitations in functionality disinclined staff to prescribe medication not immediately required, which compromised the identified communication benefits of prescribing and holding a potentially important long-term medication requiring regular review for restarting when clinically appropriate. The importance of accessibility was identified when it came to finding required information quickly, minimising loss of information within menu caches.

For peer review only - http://bmjopen.bmj.com/site/about/guidelines.xhtml

Over-reliance on the individual to undertake tasks increased the risk of them not being done.

I think, I think a lot of the errors occur because it's down to individuals to do things. And you can put systems in place, and you can put protocols in place, but people will always find a way around them, when you're busy you'll always take the easiest route.[…] Whereas, if you had ways of connecting up systems so that it's automatically done, then it's less reliant on the individuals. The individuals would still need to be involved in it and can still up on errors and add to the system, but you would probably get fewer errors, certainly less, sort of, information loss if the systems talked to each other. **(P2 Critical Care Pharmacist)**

Theme 5. Beliefs about consequences for the patient and organisation

Medication errors and adverse patient outcomes

Medication errors reported included inappropriate continuation of medication started in ICU without an ongoing indication, loss of important medication information (e.g. review plan), and failure to restart potentially important chronic medication. Although participants acknowledged such medication errors were common, the outcomes for patients and the impact upon them was not something that participants seemed to universally appreciate or take into consideration.

I've never really thought about that [potential impact on patients]*. I would hope not. I would hope by the point we get to discharge the wards have reviewed all the medications properly and done a decent* [discharge prescription] *[…]. So, they're generally on medication short term to try and help psychosis, which I would hope gets stopped before they get discharged because it's not something we ask to be on long term, but I don't know if I'm honest. It's not something that we follow up on.* (**P14 ACCP**)

Participants identified that the implications would vary depending on certain factors such as the specific medication error and timescale considered and that there was a *'very wide range of possible outcomes of omitting medicines, unintentionally or intentionally'*. **(P10 Ward Clinical Pharmacist)**

Some participants considered the potential for adverse effects as a longer-term risk and would not be apparent until sometime after the hospital discharge (e.g. unintentional discontinuation of secondary prevention medication post-myocardial infarction or in the case below of continuation of anti-psychotic medication).

We had […] a case about ten years ago where a patient was prescribed three different medicines for delirium, different psychiatric medicines while they were on unit, then discharged to the ward, went home and then eventually they went back to their GP, was asked to sign their repeat prescription three months later and he looked at it and said, I didn't know this patient was under psychiatric care. And

For peer review only - http://bmjopen.bmj.com/site/about/guidelines.xhtml

then looked into it and they'd basically never been stopped, the patient went home on anti-psychotic medicines, nobody stopped him. **(P11 Critical Care Pharmacist)**

Units that had undertaken a review on incidents, clinical audits or operated follow up clinics appeared more cognisant of potential medication issues after ICU patient transfer and implications for patient recovery. Some participants reported unit follow up of clinical incidents related to adverse drug events had been undertaken. Others had undertaken clinical audits or service evaluations of medication continuity and safety, which increased staff awareness of the high rates of medication errors on transition from ICU.

Yeah, 'cause we have had a couple of incidents and we've learned from them, and in fact our pharmacist did an audit on it, and presented that audit to the MDT, and there was a lot of learning points from that, and it has brought to us the importance, 'cause I think people just didn't, it just wasn't very high up in people's concerns about a patient. **(P12 ACCP)**

Feedback from follow up clinics were another way staff learnt about medication errors on transition and patient adverse drug events. These safety culture processes reduced the chance of siloed ICU perspectives on the patient care pathway.

Patient and family engagement

Participants identified that ICU patients frequently experienced delirium and memory loss which was a challenge to potential engagement in medication discussions and information whilst in ICU. Concerns about lack of patient recollection was a barrier to healthcare professional communication around medication handover on planned transfer to the hospital ward. It was unclear what perceived role family members had in this scenario. Whilst in ICU, the care team tended to take over all control of patient medication.

..we take complete control of their medications, nines time out of ten in hospital, so we are, you know, we are basically putting it all in our hands, and responsibility therefore. **(P9 ICU Consultant)**

Patient involvement in their medication through guiding discussions and questioning changes, was highlighted when their condition improved. Consequently, the role of the patient and family in medication safety and continuity whilst in ICU appeared passive, rather than being active agents.

And usually you find that a patient who's got long term illness, they're usually quite good at questioning medication. I can foresee that patients on intensive care, they've got poor concentration, attention, and they'll be coming to that remit of, the nurse […] gave me six tablets, I'll just take them without asking any questions, et cetera. **(P1 Outreach Team)**

For peer review only - http://bmjopen.bmj.com/site/about/guidelines.xhtml

Discussion

This is the first qualitative study focused on understanding the socio-technical factors of medication safety in patients transitioning from ICU. We identified thirteen factors within five broad themes, providing an interactive description of the factors that most strongly influenced the system performance. By highlighting the important system factors at an ICU and wider organisational levels, we provide a focus for service improvement efforts. Notably, time pressure considerations (workload, staffing, shift work, out of hours transfers, bed availability) have a marked negative effect on the system performance. We now discuss how these factors impacted on team and perceived system performance in relation to the key socio-organisational and work system components identified [29].

Care team factors. We saw diverse multiprofessional staff groups, working in ICU and hospital-ward teams, were agents in the delivery of medication continuity and safety on ICU patient transfer. The performance of care teams was affected by staff knowledge and skills in medication review and prescribing, being perceived to vary across healthcare professional groups. Here, the core stability of some ICU staff groups, benefitted their practice and experience, over staff with regular clinical rotations. Importantly, limitations in understanding of the potential impact of medication errors on patient or organisation outcomes undermined the meaning of mitigating tasks or processes undertaken, particularly amongst rotational staff groups. These limitations in ICU and ward-based staff beliefs about consequences of medication errors on this care interface present a challenge to the success of future behaviour change interventions [43].

We identified that the patient and family were often considered co-agents by ICU staff, reducing their engagement. Barriers to agent status included delirium and memory loss for patients. These findings may explain some of the limited patient awareness of medication changes identified on follow up [18]. Nevertheless, lack of communication and family support may also be factors [2]. Engaging patients and family members around safety of medication in transitions in care is recommended [7], and there are opportunities, such as written communication [44], to support the resilience and adaptability exhibited by patients and family in recovery [2], and care satisfaction [32].

Task factors. The logistical challenges of patient flow on the transition from ICU to the hospital ward are well known [32,45]. We show that medication safety for ICU patients transitioning to a hospital ward is a complex process, involving complex patients, following an uncertain care pathway, involving diverse multiprofessional healthcare staff groups and personnel organised in multiple teams with varying resources. Process complexity was further increased by being time critical and dependent. Task prioritisation was focused on the more acutely ill patients in ICU, or new patients

For peer review only - http://bmjopen.bmj.com/site/about/guidelines.xhtml

on the hospital ward, potentially affected by staff resources and physical environment factors (ICU bed availability and pressures for beds) [46]. We found some medical staff considered the medication elements were a low priority to include in ICU patient transfer and handover plans, as previously reported by others [32]. In contrast, several medication-related elements (pre-admission medicines, active medications on transfer, rationale for medication regime and intravenous infusions) were considered essential information elements by ICU and ward-based Canadian medical staff [47].

Tools and technologies factors. We report that the full integration of e-prescribing systems across ICU and the hospital ward was clearly important in reducing medication transcription errors. Progression from non-integrated to integrated ICU and hospital ward e-prescribing systems, reduced the frequency and clinical severity of medication errors on transition in ICU patient care in an Australian report [48]. Although, it was also apparent that re-prescribing on transfer provided an opportunity to prompt a structured medication review, previously being associated with reduced risk of medication transfer errors [10]. Communication of information was viewed as supported when it was easily accessible and searchable within the numerous and complicated electronic system menus. The structure and content of the medication-related communication was aided by transfer checklists and summary reports system functionality.

Organisational factors. ICU leadership was provided by the ICU consultant and it was apparent from ICU participants that team performance, as exemplified in multiprofessional ward rounds was supported by psychological safety. This is reassuring given that ward round perception on quality of leadership predicts open multiprofessional communication within ICU [49]. Indeed, ICUs with a multiprofessional ward round are associated with a marked reduction in medication transfer errors [10]. However, ICU team performance is also informed by the composition and interaction of the multiprofessional healthcare team, providing diversity of specialism working on a co-ordinated and goal orientated manner [50]. Although we identified clear professional diversity, there were also indications of opportunities to further improve team effectiveness and performance by strengthening other components (dependability, structure and clarity, meaning, impact) [51].

Communication, particularly inter-team (ICU to Ward) communication, was considered a challenge to medication safety and continuity. These staff communication challenges around patient transfer from ICU to a hospital ward are well described elsewhere [32,52,53]. Limitations in written communication content are common, lacking decision-making information such as the rationale for medications that were started or stopped [53]. Lack of communication about medication may contribute to medication errors in ICU survivors on the hospital ward [52], and inadequate handover

For peer review only - http://bmjopen.bmj.com/site/about/guidelines.xhtml

can contribute to the distress patients experience on this transition in care interface [52]. We identified several factors that facilitated communication of medication related information. These included multiprofessional handovers, prescription annotations, outreach teams following up patients and plans, and referrals to specialist teams. Previous systematic reviews [31,54], have identified the importance of handover forms and liaison nurses, improving continuity or care but without any evidence of improved patient outcomes. However, it is not enough that a handover occurred, but that the right information is conveyed, acknowledged and actioned. Automation of facets of medication-related information via technological systems improve information transfer and delivery on ICU patient transitions [55].

Time pressures and considerations affected organisational conditions with time constraints having a notable effect on system performance. The important impact of such time considerations as facilitators and barriers to care during discharge from the ICU have been recently summarised [56]. Patients transferred out of hours from ICUs have worse outcomes, [57] and it is conceivable that medication safety issues contribute to this increased risk.

Our results share consistent themes previously identified in ICU transfers and medication or care continuity. Lack of staff knowledge, unclear task allocation and staff collaboration are a barrier to medication safety on transitions in patient care, as reported for medicines reconciliation processes [58]. Variation and lack of clarity of roles around inpatient medication reviews has previously been identified as an additional risk factor for medication errors, as reported for ward-based clinical pharmacists [59]. Patient severity (complexity) of illness, provider experience (knowledge and skills) and resource constraints (workload and staff availability), have previously been identified as themes affecting ICU patient discharge and readmission risk [60].

Strengths and Limitations

Our study had several strengths. Our findings are strengthened by the inclusion of views of UK healthcare professionals from both the ICU (transferring) and the hospital ward (receiving) multicentre teams, involved in the medication review and prescribing of ICU patients. Views represented teaching and district general hospitals, all of which use electronic prescribing systems. Thematic analysis was framed on the SEIPS 3.0 model, [29] which was developed specifically for evaluating the human factors of patient safety during transitions in care. We provide system interface interactions, including indications of modifiability to assist service improvement endeavours.

For peer review only

We did not include nursing staff as participants as the focus was medication review and prescribing activities, although ACCP and Outreach Teams are primarily from professional nursing backgrounds, making it likely we captured the main themes here as well. One interview of a hospital junior doctor failed to record properly. The potential impact was minimised by use of interviewer field notes (main points summary), and we benefitted from other participant interviews from the same profession. AAA is a critical care pharmacist and there is potential for bias and positionality to influence the interpretation of the results. We are confident that any risk of these were minimised by a non-ICU healthcare professional (BBB) leading the interviews, frequent reflexive evaluation, undertaking of dual coding of all interviews, and using an iterative process to identify the themes and sub-themes based on submersion in the data and continuous matching of extracts to the thematic analysis.

Recommendations for policy change and further research

Hospital-wide integration of e-prescribing systems across ICU and the hospital ward areas is imperative across this interface in patient care. These e-prescribing systems must have the required functionality (including automation when applicable), to support delivery of medication safety, being informed by an understanding of the information and actions required to effectively undertake component tasks [26]. Patient flow systems across the ICU to ward transfer interface should be reviewed and optimised, to identify and implement modifiable elements to maximise multiprofessional team processes and care continuity within existing time constraints. Inter team communication processes around medication-information need to be streamlined, automated (when possible), co-ordinated and accessible, with structured modes of delivery. Task organisation should include delegation to specific staff groups with the knowledge and skills to routinely undertake medication reviews prior to patient transfer to a hospital ward (e.g. critical care pharmacists and ACCPs). However, for clinical pharmacists to be able to take on routine delivery of these additional roles, their availability needs to be dependable and ensure sufficient staff are available 7-days per week. In terms of team organisation, all staff need to understand their responsibilities and those of colleagues in this facet of patient care to improve team performance. Further research is required to confirm the relationship between medication transfer errors and adverse drug events [8], with ICU patient outcomes. Such evidence would inform staff beliefs about consequences, underpinning the education of staff on the clinical importance and how systems can be used to minimise MEs in this interface of care. Furthermore, this education needs to translate into ICU junior medical staff competency assessments to further support improvements in system effectiveness. Further research is required to understand how ICU patients and/ or their family should be more engaged and involved in medication-related information, enabling more participation in their own treatment and recovery.

For peer review only - http://bmjopen.bmj.com/site/about/guidelines.xhtml

Conclusions

We identified crucial factors within five key themes that ICU and ward-healthcare professionals viewed were important in the safety and continuity of medication for ICU patients transitioning to a hospital ward. The complexity of the interactions on the performance and time dependency was clear. We make recommendations for policy change and further research based on improving: knowledge and skills of staff, sufficient critical care staffing, team performance, communication and collaboration, and patient and family engagement.

Acknowledgements

The authors would like to thank all the participants for taking the time to provide their insightful views that enriched the findings of this research. We also express our gratitude to our patient and public colleagues who contributed to our interpretation of the study findings and wider dissemination of the results.

Author Contributions

RSB, DP and DMA were responsible for conception of this qualitative study. MJ undertook the participant interviews. RSB and MJ coded the transcripts and undertook the thematic analysis. RSB, DP and DMA contributed to interpretating the results. RSB, MJ, DP, JKJ, EB, FW and HM developed the representations of the work-system performance interactions. RSB & MJ wrote the manuscript. All authors read and approved the final manuscript. RSB acts as the guarantor for the intellectual integrity of the data.

Ethics Approval

Ethical (The University of Manchester, Ref: 2020-10852-17342) and NHS Health Research Authority (HRA) (IRAS 292456) approval was granted for the study.

Funding

This report is independent research supported by the National Institute for Health and Care Research NIHR300444. BBB and GGG are funded by the National Institute for Health and Care Research XXXXXX (PSTRC-2016-003). The views expressed in this publication are those of the author(s) and not necessarily those of the NHS, the National Institute for Health and Care Research or the Department of Health and Social Care.

Competing interests

The authors declare that they have no competing interests.

For peer review only

For peer review only - http://bmjopen.bmj.com/site/about/guidelines.xhtml

Patient consent

Not applicable

Provenance and peer review

Not commissioned; externally peer reviewed

Data availability statement

The datasets used and/or analysed during the current study are available from the corresponding author on reasonable request.

Supporting Information

Supplementary file

Table S1. Electronic prescribing system configurations in ICU and hospital ward at each site

Table S2. Behavioural ICU transition medication error case vignettes

Interview Topic Guide

Figure Abbreviations

Figure 1: CT: Care Team factor; TA: Task factor; TT: Tools and Technology factor; O: Organisational factor; PE: Physical Environment factor; EE: External Environment factor.

For peer review only

References

1 Rousseau AF, Prescott HC, Brett SJ, *et al.* Long-term outcomes after critical illness: recent insights. *Crit Care* 2021;**25**:1–7. doi:10.1186/s13054-021-03535-3

2 Haines KJ, Hibbert E, Leggett N, *et al.* Transitions of Care After Critical Illness-Challenges to Recovery and Adaptive Problem Solving. *Crit Care Med* 2021;**49**:1923–31. doi:10.1097/CCM.0000000000005095

3 Lone NI, Gillies MA, Haddow C, *et al.* Five-year mortality and hospital costs associated with surviving intensive care. *Am J Respir Crit Care Med* 2016;**194**:198–208. doi:10.1164/rccm.201511-2234OC

4 McPeake J, Bateson M, Christie F, *et al.* Hospital re-admission after critical care survival: a systematic review and meta-analysis. *Anaesthesia* 2022;**77**:475–85. doi:10.1111/anae.15644

5 Turnbull AJ, Donaghy E, Salisbury L, *et al.* Polypharmacy and emergency readmission to hospital after critical illness: a population-level cohort study. *Br J Anaesth* 2021;**126**:415–22. doi:10.1016/j.bja.2020.09.035

6 Brown SM, Bose S, Banner-Goodspeed V, *et al.* Approaches to addressing post-intensive care syndrome among intensive care unit survivors A Narrative Review. *Ann Am Thorac Soc* 2019;**16**:947–56. doi:10.1513/AnnalsATS.201812-913FR

7 World Health Organization. Medication Safety in Transitions of Care. 2019. https://www.who.int/publications/i/item/WHO-UHC-SDS-2019.9

8 Bosma LBE, Hunfeld NGM, Quax RAM, *et al.* The effect of a medication reconciliation program in two intensive care units in the Netherlands: a prospective intervention study with a before and after design. *Ann Intensive Care* 2018;**8**:19. doi:10.1186/s13613-018-0361-2

9 Heselmans A, van Krieken J, Cootjans S, *et al.* Medication review by a clinical pharmacist at the transfer point from ICU to ward: a randomized controlled trial. *J Clin Pharm Ther* 2015;**40**:578–83. doi:10.1111/jcpt.12314

10 Tully AP, Hammond DA, Li C, *et al.* Evaluation of medication errors at the transition of care From an ICU to non-ICU location. *Crit Care Med* 2019;**47**:543–9. doi:10.1097/CCM.0000000000003633

11 Sauro KM, Soo A, de Grood C, *et al.* Adverse events after transition from ICU to hospital ward. *Crit Care Med* 2020;**48**:946–53. doi:10.1097/CCM.0000000000004327

12 Campbell AJ, Bloomfield R, Noble DW. An observational study of changes to long-term medication after admission to an intensive care unit. *Anaesthesia* 2006;**61**:1087–92. doi:10.1111/j.1365-2044.2006.04831.x

13 Bourne RS, Ioannides CP, Gillies CS, *et al.* Clinical frailty and polypharmacy in older emergency critical care patients: A single-centre retrospective case series. *Eur J Hosp Pharm* 2021;:ejhpharm-2020-002618. doi:10.1136/ejhpharm-2020-002618

14 Royal Pharmaceutical Society. Polypharmacy: Getting our medicines right. London: 2019. https://www.rpharms.com/recognition/setting-professional-standards/polypharmacy-getting-our-medicines-right

15 Wang Y, Zhang X, Hu X, *et al.* Evaluation of medication risk at the transition of care: a cross-sectional study of patients from the ICU to the non-ICU setting. *BMJ Open* 2022;**12**:e049695. doi:10.1136/bmjopen-2021-049695

For peer review only - http://bmjopen.bmj.com/site/about/guidelines.xhtml

16 Scales DC, Fischer HD, Li P, *et al.* Unintentional Continuation of Medications Intended for Acute Illness After Hospital Discharge: A population-based cohort study. *J Gen Intern Med* 2016;**31**:196–202.

17 Bell CM, Brener SS, Gunraj N, *et al.* Association of ICU or hospital admission with unintentional discontinuation of medications for chronic diseases. *JAMA J Am Med Assoc* 2011;**306**:840–7.

18 MacTavish P, Quasim T, Purdie C, *et al.* Medication-related Problems in Intensive Care Unit Survivors: Learning from a Multicenter Program. *Ann Am Thorac Soc* 2020;**17**:1326–9. doi:10.1513/AnnalsATS.202005-444RL

19 Stollings JL, Bloom SL, Wang L, *et al.* Critical Care Pharmacists and Medication Management in an ICU Recovery Center. *Ann Pharmacother* 2018;**52**:713–23. doi:10.1177/1060028018759343

20 Bosma LBE, van Rein N, Hunfeld NGM, *et al.* Development of a multivariable prediction model for identification of patients at risk for medication transfer errors at ICU discharge. *PLoS One* 2019;**14**:e0215459. doi:10.1371/journal.pone.0215459

21 Fray M, Waterson P, Carman E. Care transitions in healthcare: The 'high-hanging fruit'. *Appl Ergon* 2021;**95**:103437. doi:10.1016/j.apergo.2021.103437

22 Co I, Hyzy RC. Lost in Transition: A call to arms for better transition from ICU to hospital Ward. *Crit Care Med* 2020;**48**:1075–6. doi:10.1097/CCM.0000000000004381

23 Rhodes A, Moreno RP, Azoulay E, *et al.* Prospectively defined indicators to improve the safety and quality of care for critically ill patients: a report from the Task Force on Safety and Quality of the European Society of Intensive Care Medicine (ESICM). *Intensive Care Med* 2012;**38**:598–605.

24 Prescott HC, Angus DC. Enhancing recovery from sepsis: A review. *JAMA - J Am Med Assoc* 2018;**319**:62–75. doi:10.1001/jama.2017.17687

25 Bourne RS, Jennings JK, Panagioti M, *et al.* Medication-related interventions to improve medication safety and patient outcomes on transition from adult intensive care settings: a systematic review and meta-analysis. *BMJ Qual Saf* 2022;**31**:609–22. doi:10.1136/bmjqs-2021-013760

26 Bourne RS, Phipps DL, Jennings JK, *et al.* Medication safety for intensive care patients transferring to a hospital ward: A Hierarchical Task Analysis. *Hum Factors Healthc* 2022;**2**:100030. doi:10.1016/j.hfh.2022.100030

27 Bourne RS, Jennings JK, Ashcroft DM. A Delphi consensus study to identify priorities for improving and measuring medication safety for intensive care patients on transfer to a hospital ward. *Int J Qual Heal Care* 2022;**34**. doi:10.1093/intqhc/mzac082

28 Sevdalis N, Brett SJ. Improving care by understanding the way we work: human factors and behavioural science in the context of intensive care. *Crit Care* 2009;**13**:139. doi:10.1186/cc7787

29 Carayon P, Wooldridge A, Hoonakker P, *et al.* SEIPS 3.0: Human-centered design of the patient journey for patient safety. *Appl Ergon* 2020;**84**:103033. doi:10.1016/j.apergo.2019.103033

30 Donchin Y, Gopher D, Olin M, *et al.* A look into the nature and causes of human errors in the intensive care unit. *Qual Saf Health Care* 2003;**12**:294–300. doi:10.1136/qhc.12.2.143

For peer review only

31 van Sluisveld N, Hesselink G, van der Hoeven JG, *et al.* Improving clinical handover between intensive care unit and general ward professionals at intensive care unit discharge. *Intensive Care Med* 2015;**41**:589–604. doi:10.1007/s00134-015-3666-8

32 Stelfox HT, Leigh JP, Dodek PM, *et al.* A multi-center prospective cohort study of patient transfers from the intensive care unit to the hospital ward. *Intensive Care Med* 2017;**43**:1485–94. doi:10.1007/s00134-017-4910-1

33 Manias E, Williams A, Liew D. Interventions to reduce medication errors in adult intensive care: a systematic review. *Br J Clin Pharmacol* 2012;**74**:411–23.

34 Bueno WP, Saurin TA, Wachs P, *et al.* Coping with complexity in intensive care units: A systematic literature review of improvement interventions. *Saf Sci* 2019;**118**:814–25. doi:10.1016/j.ssci.2019.06.023

35 McNab D, McKay J, Shorrock S, *et al.* Development and application of systems thinking' principles for quality improvement. *BMJ Open Qual* 2020;**9**:e000714. doi:10.1136/bmjoq-2019-000714

36 Scanlon MC, Karsh BT. Value of human factors to medication and patient safety in the intensive care unit. *Crit Care Med* 2010;**38**:90–6. doi:10.1097/CCM.0b013e3181dd8de2

37 Carayon P, Wetterneck TB, Cartmill R, *et al.* Characterising the complexity of medication safety using a human factors approach: An observational study in two intensive care units. *BMJ Qual Saf* 2014;**23**:56–65. doi:10.1136/bmjqs-2013-001828

38 Taylor-Adams S, Vincent C. Systems analysis of clinical incidents: the London protocol. *Clin Risk* 2004;**10**:211–20. doi:10.1258/1356262042368255

39 Reason J. *Human error*. Cambridge: Cambridge University Press 1990. doi:doi:10.1017/CBO9781139062367

40 Braun V, Clarke V. Using thematic analysis in psychology. *Qual Res Psychol* 2006;**3**:77–101. doi:10.1191/1478088706qp063oa

41 Braun V, Clarke V. To saturate or not to saturate? Questioning data saturation as a useful concept for thematic analysis and sample-size rationales. *Qual Res Sport Exerc Heal* 2021;**13**:201–16. doi:10.1080/2159676X.2019.1704846

42 Tong A, Sainsbury P, Craig J. Consolidated criteria for reporting qualitative research (COREQ): a 32-item checklist for interviews and focus groups. *Int J Qual Heal Care* 2007;**19**:349–57. doi:10.1093/intqhc/mzm042

43 Carey RN, Connell LE, Johnston M, *et al.* Behavior Change Techniques and Their Mechanisms of Action: A Synthesis of Links Described in Published Intervention Literature. *Ann Behav Med* 2019;**53**:693–707. doi:10.1093/abm/kay078

44 Tobiano G, Chaboyer W, Teasdale T, *et al.* Older patient and family discharge medication communication: A mixed-methods study. *J Eval Clin Pract* 2021;**27**:898–906. doi:10.1111/jep.13494

45 Hua M, Wunsch H. The complexities of ICU discharge. *Chest* 2015;**147**:281–2. doi:10.1378/chest.14-2377

46 Batchelor A. Adult Critical Care GIRFT Programme National Specialty Report. 2021. https://www.gettingitrightfirsttime.co.uk/wp-content/uploads/2021/08/Adult-Critical-Care-Aug21L.pdf

For peer review only

For peer review only - http://bmjopen.bmj.com/site/about/guidelines.xhtml

47 de Grood C, Job McIntosh C, Boyd JM, *et al.* Identifying essential elements to include in Intensive Care Unit to hospital ward transfer summaries: A consensus methodology. *J Crit Care* 2019;**49**:27–32. doi:10.1016/j.jcrc.2018.10.001

48 Dabliz R, Poon SK, Fairbrother G, *et al.* Medication safety improvements during care transitions in an Australian intensive care unit following implementation of an electronic medication management system. *Int J Med Inform* 2021;**145**:104325. doi:10.1016/j.ijmedinf.2020.104325

49 Reader TW, Flin R, Mearns K, *et al.* Interdisciplinary communication in the intensive care unit. *Br J Anaesth* 2007;**98**:347–52. doi:10.1093/bja/ael372

50 Ervin JN, Kahn JM, Cohen TR, *et al.* Teamwork in the intensive care unit. *Am Psychol* 2018;**73**:468–77. doi:10.1037/amp0000247

51 Google. Project aristotle. https://rework.withgoogle.com/print/guides/5721312655835136/

52 Field K, Prinjha S, Rowan K. 'One patient amongst many': A qualitative analysis of intensive care unit patients' experiences of transferring to the general ward. *Crit Care* 2008;**12**:21. doi:10.1186/cc6795

53 Brown KN, Leigh JP, Kamran H, *et al.* Transfers from intensive care unit to hospital ward: A multicentre textual analysis of physician progress notes. *Crit Care* 2018;**22**. doi:10.1186/s13054-018-1941-0

54 Wibrandt I, Lippert A. Improving Patient Safety in Handover From Intensive Care Unit to General Ward: A Systematic Review. *J Patient Saf* 2020;**16**:199–210. doi:10.1097/PTS.0000000000000266

55 Parsons Leigh J, Brundin-Mather R, Zjadewicz K, *et al.* Improving transitions in care from intensive care units: Development and pilot testing of an electronic communication tool for healthcare providers. *J Crit Care* 2020;**56**:265–72. doi:10.1016/j.jcrc.2020.01.019

56 Plotnikoff KM, Krewulak KD, Hernández L, *et al.* Patient discharge from intensive care: an updated scoping review to identify tools and practices to inform high-quality care. *Crit Care* 2021;**25**:1–13. doi:10.1186/s13054-021-03857-2

57 Vollam S, Dutton S, Lamb S, *et al.* Out-of-hours discharge from intensive care, in-hospital mortality and intensive care readmission rates: a systematic review and meta-analysis. *Intensive Care Med* 2018;**44**:1115–29. doi:10.1007/s00134-018-5245-2

58 van Sluisveld N, Zegers M, Natsch S, *et al.* Medication reconciliation at hospital admission and discharge: insufficient knowledge, unclear task reallocation and lack of collaboration as major barriers to medication safety. *BMC Health Serv Res* 2012;**12**:170. doi:10.1186/1472-6963-12-170

59 Helathcare Safety Investigation Branch. The role of clinical pharmacy services in helping to identify and reduce high-risk prescribing errors in hospital. 2020. https://www.hsib.org.uk/documents/249/hsib-report-high-risk-prescribing-errors-hospital.pdf

60 Ofoma UR, Dong Y, Gajic O, *et al.* A qualitative exploration of the discharge process and factors predisposing to readmissions to the intensive care unit. *BMC Health Serv Res* 2018;**18**:6. doi:10.1186/s12913-017-2821-z

For peer review only

For peer review only - http://bmjopen.bmj.com/site/about/guidelines.xhtml

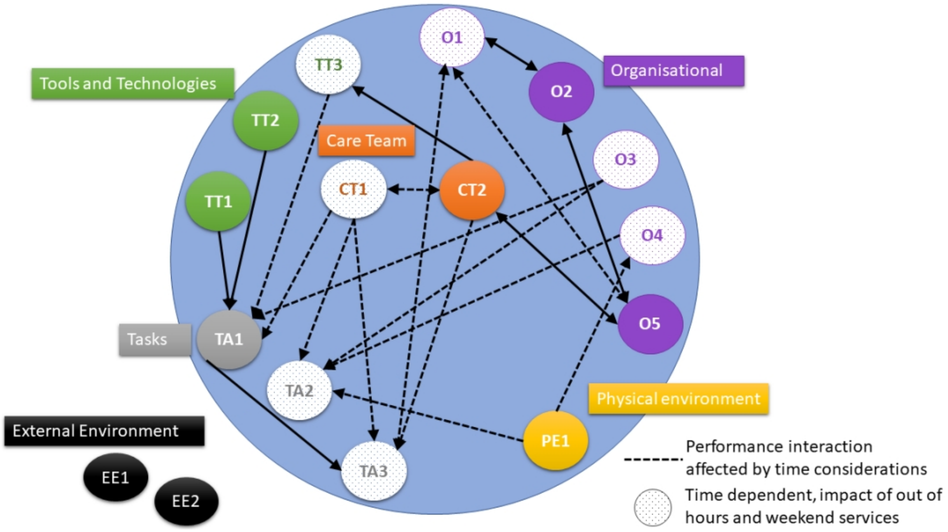

Fig 1: ICU patient transfer performance interaction

338x190mm (96 x 96 DPI)

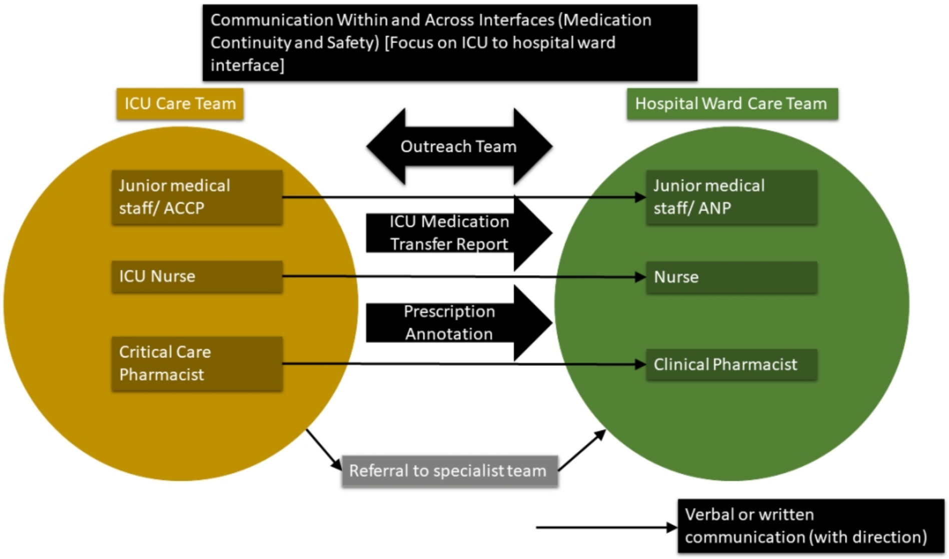

Fig 2: Medication continuity and safety communication across ICU to hospital ward interfaces

338x190mm (96 x 96 DPI)

Supplementary File

The multicentre study was conducted in 4 general intensive care units (ICUs) across 3 Trusts in the north of England (XXX (XXX NHSFT), YYY (YYY NHST), The AAA and BBB Hospital (both ZZZ NHS Trust)).

Centre	Hospital Type	ICU	Ward	Comment
XXX	Teaching	e-prescribing (MetaVision, iMDsoft)	e-prescribing (Lorenzo, DXC Technology)	No inter-system electronic communication. All ward-based infusion prescriptions on paper
YYY	Teaching	e-prescribing (MedChart)	e-prescribing (MedChart)	Inter-system communication. Complex and variable rate infusions are prescribed on paper
AAA	District General	e-prescribing (MedChart)	e-prescribing (MedChart)	Inter-system communication. Complex and variable rate infusions are prescribed on paper
BBB	Teaching	e-prescribing (Allscripts)	e-prescribing (Allscripts)	Inter-system communication. Total parenteral nutrition (TPN) prescribed on paper

Table S1. Electronic prescribing system configurations in ICU and hospital ward at each site

**Case 1 (slip and lapse type mistake)**
Error: Missed prescription for an antihypertensive drug (losartan) on ICU and hospital discharge, with that patient being hypertensive on return to GP practice.
Contextual factors: The losartan had not been prescribed in ICU as the patient had initially been hypotensive. On ICU discharge the patient was normotensive. The ICU was busy and only the medicines the patient was currently receiving whilst on ICU were transferred to the ward electronic prescription system. Losartan was on the ICU pre-admission

For peer review only - http://bmjopen.bmj.com/site/about/guidelines.xhtml

medication record, but this list was not transferred to the ward electronic system and the medicines reconciliation paper report was not reviewed on the ward.
**Case 2 (rules-based mistake)**
Error: ICU patient with Type 1 diabetes had their blood glucose controlled by a variable-rate insulin infusion. Their long-acting basal insulin was not prescribed whilst they were on a high-dose metaraminol infusion (as per ICU guidelines). The metaraminol was weaned off and the patient transitioned to the ward still without their basal SC insulin.
Contextual factors: Hospital guidelines for all Type 1 diabetic patients on a variable-rate insulin infusion is to continue their usual subcutaneous basal insulin to reduce the risk of diabetic ketoacidosis if the infusion is interrupted. ICU medical and pharmacy staff did not start the basal insulin after the metaraminol infusion was discontinued and this error was not identified prior to the ward transition. Only active medicines were transferred to the ward prescribing system. The Diabetes Specialist Team identified the error when they reviewed the patient on the ward.
**Case 3 (knowledge-based mistake)**
Error: An ICU patient receiving TPN was discharged to a ward without TPN prescribed on the paper fluid chart and didn't receive any TPN over the weekend (2 days).
Contextual factors: The trainee medical staff didn't know they needed to transfer the electronic TPN prescription from the ICU system to the paper fluid chart for the ward. The discharge was at the weekend and wasn't checked by a clinical pharmacist. Ward staff did not identify the patient was on TPN until the Nutrition Support Team reviewed the patient on Monday.

Table S2. Behavioural ICU transition medication error case vignettes

For peer review only

Interview Topic Guide

Interview questions:

Designation

1. What is your specific role at the hospital?

- How long have you been in this post?

Prior understanding of ICU patient transfer medication errors and local systems

2. Can you tell me about any prior awareness/knowledge/experience you have of any ICU patient transfer medication errors?

- If so, were these due to an error with medication review, or prescribing or both?
- In your opinion, do such errors significantly impact on patient care or not?
- Is that in the hospital or the community or both?

  (Explore if this is primarily an ICU, or hospital word, or GP issue or does it traverse all of these areas?)

  How are these errors usually detected?

3. Can you describe any procedures or safety systems are in place in your hospital to reduce medication errors on the interface of ICU patient transfers to the ward?

   *(*Is there an ICU discharge procedure? If so, please tell me about that discharge procedure? Do outreach services routinely review all patient's medicines on the ward?

4. How well are these procedures and safety systems implemented?

Who is responsible for the delivering the various safety systems mentioned?

Is there clear task allocation by profession or group?

How well are they adhered to in practice?

For peer review only - http://bmjopen.bmj.com/site/about/guidelines.xhtml

Case vignette discussions

- Case 1 (slip and lapse type mistake). Error and Contextual factors

So Case 1 is an error in which somebody forgets to do something they should or accidentally do something they shouldn't have done

5. Is this type of error common in your opinion?

How common? *[Rare to very common etc?]*

What do you think contributes most to slips and lapse type errors?

- Can signpost – London Protocol contributory influencing factors e.g. communication, workload, knowledge or skills if asked)

6. What things would help ro reduce errors like these?

   Then, if need be, prompt the participant to talk specifically about procedural/system/staff role contributions as required.

- Case 2 (rules-based mistake). Error and Contextual factors

So Case 2 is an error in which somebody incorrectly applies or doesn't apply a rule to a situation

What do you think contributes most to rule-based errors?

- Can signpost – London Protocol contributory influencing factors e.g. communication, workload, knowledge or skills if asked)

7. Is this type of error common in your opinion?
8. How common? *[Rare to very common etc?]*
9. What things would help to reduce errors like these?"

   Then, if need be, prompt the participant to talk specifically about procedural/system/staff role contributions as required.

- Case 3 (knowledge-based mistake). Error and Contextual factors

So case 3 is an error in which somebody misunderstands or misinterprets what is happening

What do you think contributes most to knowledge-based errors?

- Can signpost – London Protocol contributory influencing factors e.g. communication, workload, knowledge or skills if asked)

10. Is this type of error common in your opinion?
11. How common? *[Rare to very common etc?]*
12. What things would help ro reduce errors like these?"

For peer review only - http://bmjopen.bmj.com/site/about/guidelines.xhtml

Then, if need be, prompt the participant to talk specifically about procedural/system/staff role contributions as required.

Which error(s) do you most frequently encounter?

(Thinking about these errors in general; what is your role in mitigating the risk of these happening?

What could your role be?)

Facilitators and barriers to medication review (ICU patients on ward transfer)

Thinking about medication review for ICU patients during transfer to a ward:

How well does this process work?

What helps you to do it effectively?

What problems do you encounter?"

[*You can use the SEIPS framework to probe the participant for specific areas as required*]

Facilitators and barriers to safe prescribing and monitoring (ICU patients on ward transfer)

Thinking about prescribing and monitoring of medicines on the interface of ICU ward transfer:

How well does this process work?

What helps you to do it effectively?

What problems do you encounter?"

[You can use the SEIPS framework to probe the participant for specific areas as required]

Reducing medication review and/or medication errors on the interface of ICU patient transfer to a ward

13. If you could change one thing to improve the safety and continuity of medication on the ICU to ward interface – what would that be?
14. Have you any other comments or observations on the topic of medication continuity or safety in ICU patients on the interface of ward transfer?
15. Thank you for your time and helpful views.

For peer review only - http://bmjopen.bmj.com/site/about/guidelines.xhtml

Factor Types	Contributory influencing factor
Patient factors	Condition (complexity and seriousness)<br>Language and communication<br>Personality and social factors
Task and technology factors	Task design and clarity of structure<br>Availability and use of protocols<br>Availability and accuracy of test results<br>Decision-making aids
Individual (staff) factors	Knowledge and skills<br>Competence<br>Physical and mental health
Team factors	Verbal communication<br>Written communication<br>Supervision and seeking help<br>Team structure (congruence, consistency, leadership etc.)
Work environmental factors	Staffing levels and skills mix<br>Workload and shift patterns factors<br>Design, availability and maintenance of equipment<br>Administrative and managerial support<br>Environment<br>Physical
Organisation & management factors	Financial resources and constraints<br>Organisational structure factors<br>Policy, standards and goals<br>Safety culture and priorities
Institutional context factors	Economic and regulatory context<br>National health service executive<br>Links with external organisations

London Protocol: Framework of contributory factors influencing clinical practice

For peer review only

For peer review only - http://bmjopen.bmj.com/site/about/guidelines.xhtml

**Work System**	Team members
	Tasks
	Technologies and tools
	Organisation
	Physical environment
	External environment
**Process ("Medication Review & prescribing")**	Information gathering
	Information integration & Interpretation
	Medication plan
	Communication of the plan
	Treatment (Medication received & monitoring)
**Outcomes**	Patient (Adverse events, increased length of hospital stay, readmission to ICU or hospital, death)
	System (Effects on quality, safety, cost, efficiency, morale, public confidence in the health system)
	Learning from medication errors

SEIPS 3.0 Framework

For peer review only

Understanding medication safety involving patient transfer from intensive care to hospital ward: A qualitative socio-technical factors study

COREQ checklist

Note: in order to minimize the length of the manuscript, some of the details on the checklist (marked '*') are not included in the manuscript.

For peer review only

Guide question		*Response*	*Page number in manuscript*
1	Interviewer/facilitator	MJ conducted the interviews.	Methods Page 5
2	Credentials	MJ holds an PhD in Pharmacy Practice	Title page
3	Occupation	MJ: Research Associate in medication safety; RSB is a consultant pharmacist	Title page
4	Experience and training	MJ has extensive previous experience of undertaking qualitative research related to medication optimisation and safety in NHS healthcare environments	Methods Page 5
5	Relationship established	MJ was not known to the participants prior to the study. RB knew some of the researchers through professional networks or as co-workers	*
6	Participant knowledge of the research	Participants were made aware of the reasons for doing the research via the information which was given to the participant prior to the interview	Methods Page 4
7	Research characteristics	The researchers had identified the study topic based on a systematic review and meta-analysis they had undertaken of medication-related interventions to impact on medication safety for ICU patients transitioning to the hospital ward. The results of the systematic review identified the lack of theory informed interventions. The human factors interviews were part of a series of studies designed to identify and develop a theory informed intervention package suitable for testing in a future randomised controlled trial.	*
8	Methodological orientation and theory	London Protocol; Systems Engineering for Patient Safety (SEIPS) 3.0 Model	Methods Page 4
9	Sampling	We recruited from a purposive sample of healthcare professionals across four NHS centres chosen to represent both District General and Teaching Hospitals in the north of England. The sampling frame was Intensive Care Units (ICU) and ward-based healthcare professionals. These included medical staff, advanced critical care practitioners (ACCP), critical care	Methods Page 4

		pharmacists, ward-based clinical pharmacists and ICU outreach staff.	
10	Method of approach	Potential participants were identified via the Principal Investigators at each NHS hospital. Potential participants were invited to participate by email. Emails were directed by departmental leads or departmental email distribution lists.	Methods Page 4
12	Sample size	22 participants.	Results page 5 and Table 1
13	Non-participation	A small number of possible participants were approached by the PIs but declined to participate. This was predominantly this was for reasons of time, workload or lack of use of the system. Twenty-four participants consented, of which it was not possible to arrange an interview for 2 of these in a timely manner. Twenty-two participants were interviewed.	*
14	Setting of data collection	All interviews were conducted by MJ and took place via video telecommunication software (Microsoft Teams, Microsoft Corporation, USA)	Methods Page 4
15	Presence of non-participants	No non-participants were present	*
16	Description of sample	Twenty-two ICU and ward-based healthcare professionals were interviewed (3 ICU medical staff, 4 ACCP, 4 Critical Care Pharmacists, 4 Outreach Team Members, 3 Ward Medical Staff, 4 Ward Clinical Pharmacists.	Results Page 5 and Table 1
17	Interview guide	The semi-structured interview schedule was informed by London Protocol and Systems Engineering for Patient Safety (SEIPS) 3.0 Model. These facilitated capture the process, contributing factors and context of medication safety at ICU to ward transition. In order to prompt discussion, we created three medication safety error case vignettes each representing a type of error. The vignettes were shared with participants in advance of the interview.	Methods Page 4
18	Repeat interviews	No follow-up interviews were conducted	
19	Audio/visual recording	Interviews took place via video telecommunication software (Microsoft Teams, Microsoft Corporation, USA) with consent from the participant.	Methods Page 4
20	Field notes	None	Strengths and Limitations Page 21
21	Duration	Interviews ranged in duration from 30-67 minutes with a mean length of 49 minutes.	*
22	Data saturation	Data collection continued until saturation was reached and no new themes emerged from the interviews.	*
23	Transcripts returned	No transcripts were returned to participants	*
24	Number of data coders	Coding was undertaken in duplicate by the qualitative researcher (MJ) and Chief Investigator (RSB)	Methods Page 5
25	Description of the coding tree	Coding was informed by London Protocol and SEIPS 3.0 model frameworks. Coding was completed in three rounds with discussions between the coders after each round	Methods Page 5
26	Derivation of themes	We identified 13 factors in five broad themes. The thematic analysis was then developed to provide a detailed description of the ICU-hospital work-system interface	Results Page 5 Results page 16, Table 2,

For peer review only

			Figures 1 &2
27	Software	QSRNvivo 12 software was utilised to manage the data	Methods Page 5
28	Participant checking	No	*
29	Quotations presented	Please see the results section of the manuscript	Results Page 5-16, Table 2, Figures 1 &2
30	Data and findings consistent		
31	Clarity of major themes		
32	Clarity of minor themes		

For peer review only
