# Supplementary material for: Understanding medication safety involving patient transfer from intensive care to hospital ward: a qualitative sociotechnical factor study
Source: BMJ Open. 2023 May 2;13(5):e066757. doi: 10.1136/bmjopen-2022-066757 (PMC10163459; doi:10.1136/bmjopen-2022-066757)
Supplement: Reviewer comments [file bmjopen-2022-066757.reviewer_comments.pdf]

PEER REVIEW HISTORY

BMJ Open publishes all reviews undertaken for accepted manuscripts. Reviewers are asked to complete a checklist review form (**http://bmjopen.bmj.com/site/about/resources/checklist.pdf**) and are provided with free text boxes to elaborate on their assessment. These free text comments are reproduced below.

ARTICLE DETAILS

**TITLE (PROVISIONAL)**	Understanding medication safety involving patient transfer from intensive care to hospital ward: A qualitative socio-technical factors study
**AUTHORS**	Bourne, Richard; Jeffries, Mark; Phipps, Denham; Jennings, Jennifer K; Boxall, Emma; Wilson, Franki; March, Helen; Ashcroft, Darren

VERSION 1 – REVIEW

**REVIEWER**	Danielis, Matteo<br>Università degli Studi di Udine
**REVIEW RETURNED**	18-Aug-2022

**GENERAL COMMENTS**	Thank you for the opportunity to review the manuscript entitled “Understanding medication safety involving patient transfer from intensive care to hospital ward: A qualitative sociotechnical factors study” which aims to understand the socio-technical factors affecting medication safety when ICU patients are transferred to a hospital ward.<br>Page 3. An abstract has been reported. Page 5. Another (and longer) abstract has been reported.<br>Background<br>The first impression is that it is definitely redundant. It is very long, unnecessarily long. I would have preferred to read more information about medication safety, which is the focus of the manuscript.<br>Which medications are commonly used in the transfer phase?<br>What are the ICU settings mostly explored (e.g. general vs. specialist ICU) and the discharging wards?<br>Which types of medication errors have been reported to date?<br>Have been adopted strategies or policies to overcome these issues?<br>Methods<br>Information of the ethical and institutional approvals should be reported at the end of the methods section.<br>“Our study used a qualitative design” the authors should be put a reference and describe with more details the qualitative approach used (e.g. descriptive, thematic analysis).<br>In addition, please declare that you have followed the COREQ checklist.<br>“Patient and Public Involvement” there is no need to put this part in a sub-heading.<br>Results<br>The presentation of results processed with N-vivo is nice. However, some redundant information are reported in Fig.1 and Table1. Perhaps the graph may be improved (e.g., solve the

	acronyms as CT1, or insert a legend). In this case, Table 1 can be added as supplementary material.<br>The quotes reported in the presentation of the themes are too long. I think it is not necessary to report such long sentences from the interviewed healthcare professionals.<br>Discussion<br>The discussion retraces the themes emerged from the results by integrating them with the literature.<br>Overall, the manuscript is well written.

**REVIEWER**	Baysari, Melissa<br>The University of Sydney Faculty of Medicine and Health
**REVIEW RETURNED**	17-Jan-2023

**GENERAL COMMENTS**	This is a well-executed and written study examining the ICU-ward transition using a HF approach. I enjoyed reading this paper, but have some suggestions for improvement:<br>- Can the participant details, including Table 1, be included in the results, rather than method section?<br>- In your methods section, can you provide more detail on how you determined the factors that most strongly impacted this work system? Was it frequency of reporting? And how did you combine the results from the interviews and clinical vignettes? Were they all coded in the same way?<br>- In Table 2, is it possible to somehow indicate which of the performance shaping factors were positive and which were negative?<br>- Did you find any differences across sites? I would imagine the work systems to be impacted by culture, staff resources, IT systems etc. For example, were ICU outreach teams in place in all hospitals? Was it possible to compare across sites? If not, why not?<br>- In the same way, are you able to describe any contrasting views between participants working in the ICU and those on the wards? Were they consistent in their experiences and views of patient transitions?<br>- it was not clear to me why you highlight in your abstract and introductory paragraph of your discussion the time pressure factors, when this was just 1 of many barriers identified. Why mention only this?<br>- I was a little disappointed by the recommendation section of this paper. As a paper that adopted a HF approach to data collection, it would have been nice to see that HF approach follow through more into the recommendations made. That is, instead of focusing on the people performing the work (i.e. education and beliefs, more engagement, 'dependable' staff) why not spend more of this section outlining work-system redesign to support the work (e.g. integrated IT systems, automation, more staff to relieve time pressure, etc)?<br>- Given the role outlined in the paper of patients and the public (in the methods section), does this warrant co-authorship or at least an acknowledgment?

VERSION 1 – AUTHOR RESPONSE

**Reviewer: 1**	

Page 3. An abstract has been reported. Page 5. Another (and longer) abstract has been reported.	We have now rectified this and only enclose the recommended structured abstract. We have also updated the Abstract Conclusion to be consistent with the focus on system aspects requested by Reviewer #2
Background<br>The first impression is that it is definitely redundant. It is very long, unnecessarily long. I would have preferred to read more information about medication safety, which is the focus of the manuscript.<br>Which medications are commonly used in the transfer phase?<br>What are the ICU settings mostly explored (e.g. general vs. specialist ICU) and the discharging wards?<br>Which types of medication errors have been reported to date?<br>Have been adopted strategies or policies to overcome these issues?	We have revised and shortened the Background to make it more focussed on the medication safety aspects whilst still identifying the importance of the socio-technical aspects of the system performance. We provide more details on types of medication errors and strategies employed to date, including impact on reducing the risks of potentially inappropriate acute medication continuing. We have also acknowledged our recent systematic review which provides answers to these questions in much more detail (including study settings and specialties) than we could include in a paper's Background section.
Methods<br>Information of the ethical and institutional approvals should be reported at the end of the methods section.	We have moved the ethical approval information to the end of the Methods section
"Our study used a qualitative design" the authors should be put a reference and describe with more details the qualitative approach used (e.g. descriptive, thematic analysis).	We have added a reference here to thematic analysis, which adds to the previous description to the thematic analysis undertaken. [Page 11, Line 98 (Note: Page no.s refer to marked copy)]: "Transcripts were anonymised prior to deductive thematic analysis [40], using the London Protocol [38], and SEIPS 3.0 model [29] frameworks."
In addition, please declare that you have followed the COREQ checklist.	We followed the COREQ checklist and had enclosed with the submitted manuscript. We now also highlight this approach in the Methods.<br><br>[Page 12 Line 115]: "We have adhered to the Consolidated Criteria for Reporting Qualitative Studies (COREQ) in reporting our findings [42]."
"Patient and Public Involvement" there is no need to put this part in a sub-heading.	We had removed this subheading on resubmission but then we were asked to add it back in by the BMJ Open editorial office. "Authors must include a statement in the methods section of the manuscript under the sub-heading 'Patient and Public Involvement'"
Results<br>The presentation of results processed with N-vivo is nice. However, some redundant	Thank you for this suggestion. We have already included factor group labels in close proximity to the individual factors (e.g. "Care Team" label

information are reported in Fig.1 and Table1. Perhaps the graph may be improved (e.g., solve the acronyms as CT1, or insert a legend). In this case, Table 1 can be added as supplementary material.	beside CT1 and CT2) We have further improved Figure 1 by adding an explanation of factor abbreviations [Page 31, Line 635] to the Figure 1 legend. However, we believe that Table 2 complements and adds important understanding to the factors, their interaction and the complexity of the system represented in Figure 1. For example, CT1 (Care Team factor 1) is explained in Table 2 as "CT1: Knowledge and skills of staff on medication review and safe prescribing for ICU patients on the interface of transition to the hospital ward*"<br><br>Reviewer 2 has requested additional information in Table 2 identifying the direction/ effect identified impact factors had on system performance.<br><br>We have therefore kept Table 2 in the main manuscript. To meet the suggestions made by Reviewer 2
The quotes reported in the presentation of the themes are too long. I think it is not necessary to report such long sentences from the interviewed healthcare professionals.	We have reviewed all quotes included, reducing where possible the length whilst remaining true to the participants', view, context and content.
Discussion<br>The discussion retraces the themes emerged from the results by integrating them with the literature.	Thank you
Overall, the manuscript is well written.	Thank you, we appreciate this positive feedback
**Reviewer: 2**	
This is a well-executed and written study examining the ICU-ward transition using a HF approach. I enjoyed reading this paper, but have some suggestions for improvement:	Thank you. We are grateful for the comments to improve our manuscript
Can the participant details, including Table 1, be included in the results, rather than method section?	We have now moved Table 1 to the Results section
In your methods section, can you provide more detail on how you determined the factors that most strongly impacted this work system? Was it frequency of reporting?	We had already included in the Methods section - "Finally, through multiple discussions amongst the research team, we coalesced the identified factors until they provided a stable and parsimonious account of the interactions that most strongly influence the performance of the ICU to hospital ward work system and communication pathways."

	We have now added in the Methods section that we derived the work system factors and interactions from the thematic analysis of the interviews and these were then confirmed by and expert input from the research team.<br><br>Page 11, Line 101]: "All transcript content was coded using the thematic analysis." and expanded the explanation in [Page 11, Line 105]: "Finally, through multiple discussions amongst the research team, we identified main themes as being as those that provided the most meaningful pattern across the data set. We coalesced the identified "main" factors until they provided a stable and parsimonious account of the interactions that most strongly influence the performance of the ICU to hospital ward work system and communication pathways."
And how did you combine the results from the interviews and clinical vignettes? Were they all coded in the same way?	The case vignettes provided an opportunity to engage the participants in the subject matter and prompt further discussion as indicated in the Topic guide. [Page 11; Line 91]: "To prompt discussion, we selected three reported ICU medication error cases as vignettes, each representing a type of human error from Reason's (1990) generic error modelling system [37]."<br><br>We have now clarified that all transcript content was coded the same way.<br><br>[Page 11 Line 101]: "All transcript content was coded using the thematic analysis."
In Table 2, is it possible to somehow indicate which of the performance shaping factors were positive and which were negative?	Many of the performance shaping factors were bi-directional. That is if "knowledge" was good then performance was higher and conversely if it was more limited, then performance reduced. However, to make this explicit we have now added an indicator after each factor in Table 1 as [Effect dependent on status] [Positive effect] or [Negative effect].
Did you find any differences across sites? I would imagine the work systems to be impacted by culture, staff resources, IT systems etc. For example, were ICU outreach teams in place in all hospitals? Was it possible to compare across sites? If not, why not?	You are quite right that variability in resources could impact on system performance. The integrated e-prescribing system is the most notable example of this, along with critical care pharmacy staff availability. We describe the differences in IT configuration between the hospitals in Table S1 (supplementary file). However, all hospitals had all staff groups described "The sampling frame was ICU

	(medical staff, advanced critical care practitioners (ACCPs), critical care pharmacists and ICU outreach team members) and ward-based (medical staff, clinical pharmacists) healthcare professionals.”. So, all had critical care outreach teams and advanced critical care practitioners. The themes and factors identified were consistent across all hospitals. We now highlight both in our Discussion policy recommendations “Hospital-wide integration of e-prescribing systems across ICU and the hospital ward areas, with the functionality (including automation when applicable), capable of supporting medication review and communication, is imperative. Patient flow systems across the ICU to ward transfer interface should be reviewed and optimised to identify and implement modifiable elements to maximise multiprofessional team processes and care continuity within existing time constraints. Inter team communication processes around medication-information need to be streamlined, automated (when possible), co-ordinated and accessible, with structured modes of delivery. Task organisation should include delegation to specific staff groups with the knowledge and skills to routinely undertake medication reviews prior patient transfer to a hospital ward (e.g. critical care pharmacists and ACCPs). However, for clinical pharmacists to be able to take on routine delivery of these additional roles, their availability needs to be dependable and ensure sufficient staff are available 7-days per week.”<br><br>Finally, we have now included in Table 2 a direction of effect for all factors we identified as strongly impacting on system performance (I.e. “Effect dependent on status, negative or positive effect”), which we feel captures any differences in factor resources e.g. “-O2: Multiprofessional ward rounds including a medication review element* [Positive effect]”
In the same way, are you able to describe any contrasting views between participants working in the ICU and those on the wards? Were they consistent in their experiences and views of patient transitions?	Participant views were remarkably consistent across the hospitals despite providing a mixture of teaching and non-teaching hospitals with varying e-prescribing resources.<br><br>We do highlight in the text when staff groups differed in views or priorities e.g. “Amongst medical staff, transfer planning around medication was often allocated to a junior team member and this was seen as a “low-level” task. Whereas critical care pharmacists and ACCPs considered these more complex tasks.” These

	were professional differences rather than clinical area differences.
- it was not clear to me why you highlight in your abstract and introductory paragraph of your discussion the time pressure factors, when this was just 1 of many barriers identified. Why mention only this	We highlighted the various forms of time pressure that dramatically negatively impacted on the performance of the system and increased its vulnerability to medication errors. We illustrate this in Figure 1 showing how many of the system interactions were negatively impacted by time pressures. We have now clarified this finding by stating in the Results "The vulnerability of the system to time constraints was evident (Fig 1)." in Page 13 line 126.<br><br>Also, by indicating the impact direction of identified factors on the system performance in Table 2, for the 4 key factors with negative impact (TA1, TA2, O4 & EE2), all have time considerations, and for three of these (TA2, O4 & EE2), time constraints are a focus.
I was a little disappointed by the recommendation section of this paper. As a paper that adopted a HF approach to data collection, it would have been nice to see that HF approach follow through more into the recommendations made. That is, instead of focusing on the people performing the work (i.e. education and beliefs, more engagement, 'dependable' staff) why not spend more of this section outlining work-system redesign to support the work (e.g. integrated IT systems, automation, more staff to relieve time pressure, etc)?	We agree and have restructured this section so that a systems-based approach is more fully emphasised to improve care continuity and safety. We now start this section with a call for integrated and functional e-prescribing systems to be employed hospital-wide. "Hospital-wide integration of e-prescribing systems across ICU and the hospital ward areas, with the functionality to support medication review and communication, is imperative" and include system mitigation of modifiable time-pressures centred around flow of patient transfer to the wards. Finally, we identify the importance of staff availability to deliver tasks and processes related to medication safety. Similarly, we have focused on beliefs about consequences to guide staff education, understanding and prioritisation of medication continuity and safety on ICU patient transfer.<br><br>We have also updated the Abstract Conclusion to be consistent with the above changes.
Given the role outlined in the paper of patients and the public (in the methods section), does this warrant co-authorship or at least an acknowledgment?	We agree this is an oversight and have added an acknowledgement to our PPI colleagues for their contribution to the interpretation of the findings outlined in this paper.<br><br>Page "We also express our gratitude to our patient and public colleagues who contributed to

	our interpretation of the study findings and wider dissemination of the results.”

VERSION 2 – REVIEW

**REVIEWER**	Danielis, Matteo<br>Università degli Studi di Udine
**REVIEW RETURNED**	25-Mar-2023

**GENERAL COMMENTS**	I re-evalaute this manuscript entitled " Understanding medication safety involving patient transfer from intensive care to hospital ward: A qualitative sociotechnical factors study". I have to declare no potential biases in reviewing this manuscript. The manuscript addresses a relevant problem as medication safety.<br>The authors amended most the issues I've addressed to them.<br>The authors should put a reference and describe with more details the qualitative approach used (e.g. descriptive, thematic analysis).

**REVIEWER**	Baysari, Melissa<br>The University of Sydney Faculty of Medicine and Health
**REVIEW RETURNED**	19-Mar-2023

**GENERAL COMMENTS**	The authors have done well in addressing reviewer suggestions and the paper is good shape.

VERSION 2 – AUTHOR RESPONSE

**Reviewer: 1**	
The authors should put a reference and describe with more details the qualitative approach used (e.g. descriptive, thematic analysis).	We believe we have already responded to this request in our previous revision. We identify that we did use a descriptive, thematic analysis approach and provide a supporting reference to the work done by Braun and Clarke in this area of thematic analysis in qualitative research.<br><br>We include (Page 6; Line 75):<br><br>“Transcripts were anonymised prior to deductive thematic analysis [40], using the London Protocol [38], and SEIPS 3.0 model [29] frameworks.”
**Reviewer: 2**	
The authors have done well in addressing reviewer suggestions and the paper is good shape	Thank you. We appreciate this positive feedback
